# Clinical outcomes in various coronary artery bypass grafting techniques: A systematic review and network meta-analysis

**DOI:** 10.1371/journal.pone.0360757

**Published:** 2026-11-05

**Authors:** Dudy Arman Hanafy, Theresia Feline Husen, Ananda Pipphali Vidya, Diski Saisa, Derren David Christian Homenta Rempengan, Defin Allevia Yumnanisha, Ruth Angelica, Muhammad Fahd Abdurrahman

**Affiliations:** 1 Faculty of Medicine, University of Indonesia, Salemba, Jakarta, Indonesia; 2 Division of Cardiothoracic and Vascular Surgery, Department of Surgery, Faculty of Medicine, University of Indonesia, Jakarta, Indonesia; 3 Division of Adult Cardiac Surgery, Department of Surgery, National Cardiovascular Center, Harapan Kita, Jakarta, Indonesia; 4 Faculty of Medicine, Universitas Sam Ratulangi, Manado, Indonesia; Azienda Ospedaliero Universitaria Careggi, ITALY

## Abstract

**Introduction:**

There are different coronary artery bypass grafting (CABG) techniques, conventional (C-CABG), minimally invasive (MI-CABG), and robotic (R-CABG), each with its respective advantages and drawbacks. This study aimed to compare all three techniques and their nine sub-technique variations to provide a reference for the clinical selection of the most suitable surgical approach.

**Methods:**

A systematic search was applied to seven databases to find observational studies or randomized controlled trials (RCTs) that compared outcomes between CABG techniques in patients. Critical appraisal of observational studies (Newcastle–Ottawa Scale) and RCTs (Cochrane Risk of Bias 2.0) was performed. We used frequentist network meta-analysis to rank and analyze the pooled odds ratios and mean differences of each intervention. Primary outcomes were mortality and complete revascularization, while the rest were secondary outcomes. The protocol was registered with the ID CRD42023480325.

**Results:**

A total of 132 studies with 779,773 patients were included. All three techniques were associated with comparable revascularization rates. However, in the comparison of the nine sub-techniques port access (PA)-CABG was associated with a significantly higher rate compared to C-ONCAB. R-CABG demonstrated the most favorable outcomes in terms of ICU stay, overall hospital stay, mortality, and ventilation time compared to MI-CABG and C-CABG. Additionally, R-CABG was associated with lower rates of wound infection, cerebrovascular accident, and renal failure. Both R-CABG and MI-CABG were associated with lower rates of non-sinus rhythm compared to C-CABG. While MI-CABG was associated with the highest reoperation rate.

**Conclusion:**

R-CABG suggested promising outcomes over MI-CABG and C-CABG. However, as this exploratory NMA was predominantly based on observational data, these findings should be regarded as a hypothesis-generating framework for future research. Clinicians should interpret results cautiously, prioritizing patient-specific factors and institutional experience until higher-level evidence emerges.

## 1. Introduction

Coronary artery disease (CAD) is one of the most prevalent cardiovascular disease [1,2] which often requires revascularization through percutaneous coronary intervention (PCI) or coronary artery bypass grafting (CABG). Currently, a variety of CABG techniques are available to treat CAD. These are mainly categorized into conventional (C-CABG), minimally invasive (MI-CABG), and robotic CABG (R-CABG). C-CABG is still frequently performed due to lower technical demands and a shorter learning curve. However, it is associated with inferior aesthetic outcomes along with longer recovery times and an increased risk of complications such as bleeding [3,4]. These disadvantages are addressed by less invasive techniques such as MI-CABG and R-CABG, which offer shorter hospital stays, less surgical scar, and lower complication rates in wound infection and bleeding. However, these less invasive techniques require a steeper learning curve for the surgeon [3,5]. Furthermore, R-CABG is also associated with other challenges such as higher costs compared to C-CABG and MI-CABG [6]. The conflicting benefits and drawbacks associated with each technique result in the choice of technique being based on the surgeon’s evaluation of each patient’s case and individual preferences [7–9]. Hence, this study aimed to compare all the three main techniques (R-CABG, MI-CABG, C-CABG) using various patient outcomes. In addition, this study also compared a total of nine sub-techniques derived from the three main techniques (R-CABG: robotic-assisted direct (RADCAB) and totally endoscopic (TECAB); MI-CABG: minimally invasive direct (MIDCAB), minimally invasive cardiac surgery (MICS), port-access (PA-CABG), and endoscopic (Endo-CABG)); and C-CABG (conventional on-pump (C-ONCAB), beating-heart (BH-ONCAB), and off-pump (OPCAB)).

## 2. Methods

### 2.1. Materials and methods

Our review was conducted in accordance with the guidelines outlined in the Cochrane Handbook for Systematic Reviews of Interventions version 6.2 and was reported using the Preferred Reporting Items for Systematic Review and Meta-Analysis (PRISMA) for NMA [10]. Subsequently, we registered the protocol with the International Prospective Register of Systematic Reviews (PROSPERO), under the ID CRD42023480325 (accepted on November 6^th^, 2023; updated May 20^th^, 2025).

### 2.2. Search strategy

A comprehensive literature search was performed by five authors in Medline, Scopus, Cochrane, Google Scholar, Embase, Wiley, and ProQuest, up to March 18^th^, 2025. The keywords used in the search were CABG, robotic, minimally invasive, and conventional, along with their synonyms and sub-technique names (RADCAB, TECAB, MIDCAB, MICS, PA-CABG, Endo-CABG, C-ONCAB, BH-ONCAB, OPCAB) (appendix A.1 in S1 Appendix). When applicable and available in each database, advanced search techniques were used to increase search precision. All findings were imported into Rayyan automatic tools for further screening. All duplicate studies were automatically removed, while further screening was performed manually.

### 2.3. Study eligibility criteria

Studies were screened according to the following inclusion and exclusion criteria: The inclusion criteria were: (1) Observational (prospective (collected in real time) or retrospective (previously recorded medical records) or randomized controlled trial (RCT); (2) studies comparing the results between robotic vs. conventional and/or minimally invasive surgery along with their sub-techniques; and (3) coronary artery bypass graft surgery. Exclusion criteria were: (1) studies with incomplete data (absence of necessary statistical measure), or only qualitative outcome data, and (2) studies lacking a control group or involving only a single-arm design. The planned procedure is illustrated in Fig 1.

**Fig 1:**
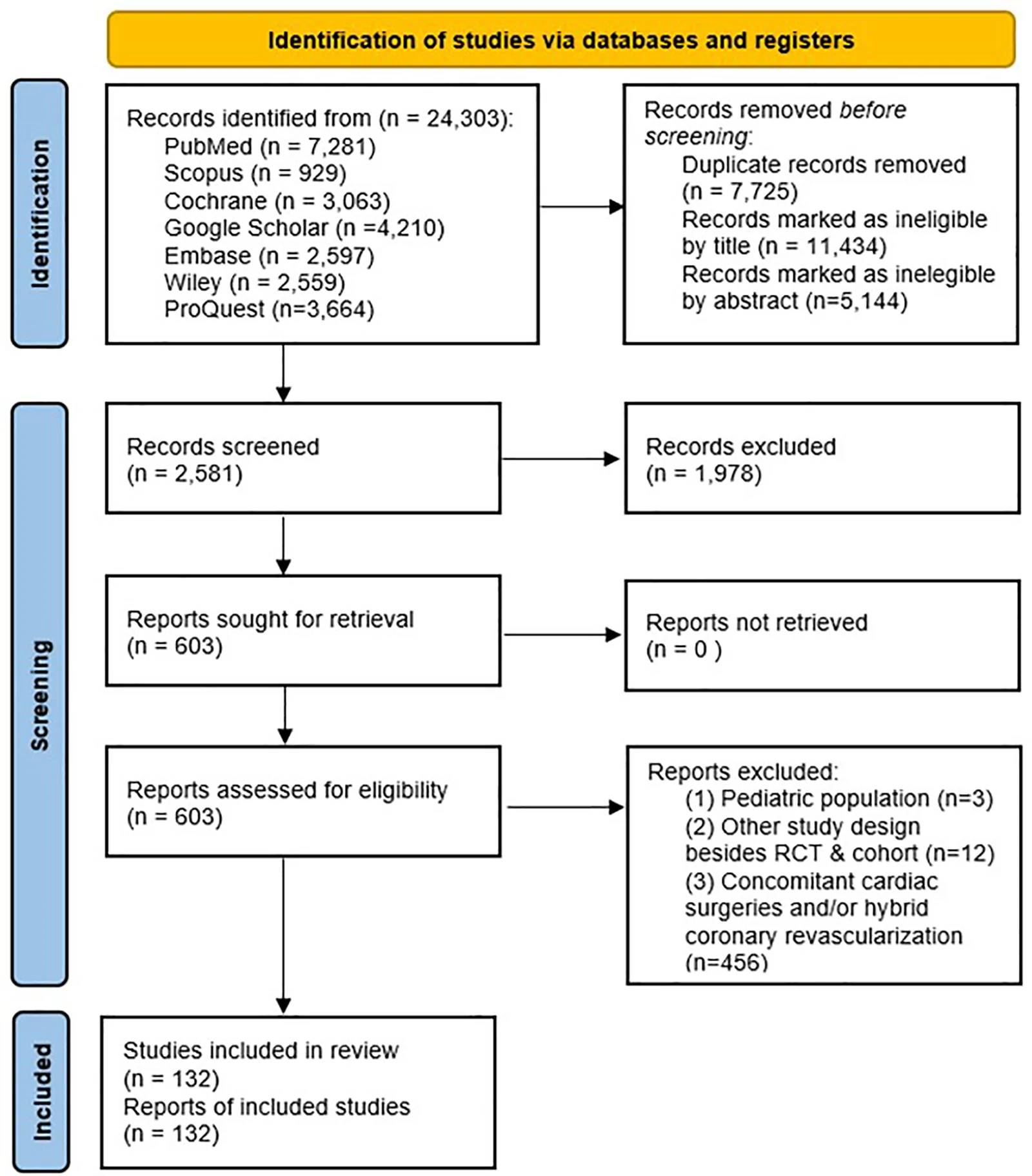
PRISMA Flow.

### 2.4. Data synthesis

We predetermined the outcome sheet in tabular form (MS Excel® for Mac; Microsoft Corporation, Redmond, WA, 2018) to include the following data to be extracted: (1) author and year of publication; (2) study characteristics, including study design, interval, location, and follow up duration during the study; (3) study population (sample size, age, follow-up period, gender, comorbidities, form of intervention, and preoperative data); and (4) study outcomes. Our study outcomes were categorized into three main sections: hospital stay, operative procedure, and postoperative outcomes. The hospital stay section consists of the length of stay in the ICU and the overall hospital stay. The operative procedure section included operative time. The final section addressed postoperative outcomes, which included mortality, complete revascularization (functionally defined as revascularization of all segments with significant ischemia or the surgeon’s intraoperative assessment of adequacy), cerebrovascular accident (CVA) (a new focal neurological deficit lasting >24 hours), myocardial infarction (MI) (new electrocardiographic changes or biomarker elevations), non-sinus rhythm (non-SR), reoperation, ventilation time, wound infection, and renal failure (new requirement for dialysis or a significant rise in creatinine level (doubling of baseline)). The primary outcomes were mortality and complete revascularization. A qualitative assessment of transitivity was performed by reviewing key study characteristics.

In this data extraction, five authors (TFH, APV, DAY, MFA, RA) extracted both qualitative and quantitative characteristics. In addition, three authors (TFH, DS, DDCR) verified the accuracy of the extracted data while performing the statistical analysis.

For RCTs, outcome data were analyzed according to the intention-to-treat (ITT) principle when reported. If ITT data were unavailable, as-treated results were used as specified in the original study. For observational studies, outcomes were analyzed based on as-treated principles, reflecting the treatment the patients actually received.

### 2.5. Statistical analysis

We applied the "meta" package for single-arm meta-analyses and frequentist NMA using the "netmeta" package version 2.8-2 of R software (version 3.4.0; R Foundation, Vienna, Austria) [11,12]. The transitivity assumption was evaluated by statistically comparing the distribution of potential effect modifiers, including age, gender, urgency of the procedure, operative risk (EuroSCORE II), number of diseased vessels, left ventricular ejection fraction, and study enrollment start year, across studies using single-arm meta-analyses. Furthermore, to address potential heterogeneity among studies, we employed a random-effect consistency model. Heterogeneity was evaluated using I^2^ statistics, with adherence to the Cochrane threshold. We utilized cut-off values of 0%, 25%, 50%, and 75% to categorize heterogeneity as insignificant, low, moderate, and high, consecutively. Sensitivity analyses were conducted for outcomes with the heterogeneity exceeding 25% to assess the robustness of the findings, particularly concerning each technique and study [13].

Publication bias was evaluated using both qualitative methods, such as funnel plots, and quantitative methods, such as Egger’s linear regression test [14]. If significant bias was detected, we applied other methods suitable for NMA, such as the Thompson-Sharp [15,16] and Begg-Mazumdar tests [17] to increase the validity of the bias assessment results.

We categorized ventilation time, operative time, ICU stay, and length of stay as continuous variables, while the remaining variables were considered dichotomous. Continuous outcomes were presented as mean differences (MDs) and standard deviations (SDs), whereas dichotomous data were expressed as odds ratios (ORs), both accompanied by a 95% confidence interval. A 95% CI that did not include 0 was considered statistically significant for continuous outcomes. For dichotomous outcomes significance was defined as a 95% confidence interval that did not include 1. These principles were applied unless the Benjamini-Hochberg false discovery rate (FDR) correction test indicated otherwise. Reflective ranking of treatments based on p-score was utilized. These are based on their reflective effectiveness that reflects the mean extent of certainty that a given treatment is better than the other competing treatments [18]. When conducting reflective ranking via the forest plot, a reference treatment (C-CABG or C-ONCAB) was used. A higher ranking indicated fewer complications and shorter durations. Furthermore, the node-splitting method was employed to assess the coherence (consistency) of the NMA between direct and indirect evidence [19].

### 2.6. Risk of bias

All the observational studies were appraised using the Newcastle-Ottawa Quality Assessment Scale for Cohort Studies to qualitatively assess the bias of the included studies. Scores were converted to the Agency for Healthcare Research and Quality (AHRQ) standards, which were divided into >6: good; 4–6: fair; 4: poor [20,21]. For RCTs, Cochrane Risk of Bias (RoB) 2.0 was applied and visualized using RoB [22]. Five reviewers (TFH, APV, DAY, MFA, RA) performed the quality assessment independently and then discussed results until a consensus was reached. In addition, to limit the risk of bias due to the difference in baseline patients’ risk profiles, for observational studies, we included only the propensity score-matched populations in the NMA whether available; however we listed all the baseline characteristics before and after matched in the systematic review.

### 2.7. Certainty of evidence

The certainty of the evidence for the primary and secondary outcomes for the three main techniques (C-CABG, MI-CABG, R-CABG) were estimated based by the Grading of Recommendations Assessment, Development, and Evaluation (GRADE) approach. For the nine sub-techniques, only the outcomes with a direct comparison were assessed. These assessments were performed by three independent authors (TFH, APV, DAY) [23].

## 3. Results

### 3.1. Study selection and characteristics

A total of 24,303 studies were identified through searches across seven databases, with detailed information provided in Appendix A in S1 Appendix. Following deduplication and the exclusion of irrelevant studies, 603 records underwent full-text review. Ultimately, 132 studies were selected for qualitative and quantitative analysis (Fig 1). These 132 studies consisted of 8 RCTs, 80 cohort retrospective studies (of which 3 obtained data from registries (no deduplicates identified), 44 were prospective studies, and 14 studies involving propensity score matching. Six RCTs compared C-CABG techniques (C-ONCAB (1,198 patients), OPCAB (1,139 patients), and BH-ONCAB(119 patients)), while 2 studies compared MIDCAB (154 patients) with OPCAB (152 patients), totaling of 2,762 patients. The characteristics of the included studies are outlined in Appendix B.1 in S2 Appendix [24–158], which also features information on patients’ underlying diseases and preoperative data before CABG.

The overall included studies spanned three continents: America, Asia, and Europe, encompassing 779,773 patients (Fig 2). The majority of participants were male (72.0%, 95% CI: 71.9–72.1), with a mean age of 63.6 years (95% CI: 63.3–63.9). Most patients included in this network meta-analysis underwent elective CABG (90.8%, 95% CI: 89.2–92.4) with a comparable operative risk score, EuroScore II 3.02 (95% CI 1.08 to 4.96). Among the 120 studies with available enrollment start years, 61 studies (50.8%, 95% CI 41.9%–59.8%) began enrollment in or before 2005, 21 studies (17.5%, 95% CI 10.7%–24.3%) started between 2006 and 2009, and 38 studies (31.7%, 95% CI 23.4%–40.0%) began enrollment after 2009. For studies reporting the robotic technique, 16 (66.7%) began enrollment in or before 2005, 3 (12.5%) between 2006 and 2009, and 5 (20.8%) after 2009. For MI-CABG, 15 (53.6%) were ≤2005, 4 (14.3%) in 2006–2009, and 9 (32.1%) after 2009. For C-CABG, 78 (63.4%) were ≤2005, 22 (17.9%) in 2006–2009, and 23 (18.7%) after 2009. Across the studies, participants were generally comparable in terms of age, gender, urgency of the procedure, operative risk, and study period supporting the assumption of transitivity for these effect modifiers. However, significant differences were observed in two other potential effect modifiers: the number of diseased vessels and left ventricular ejection fraction. Patients undergoing C-CABG tended to have a significantly lower ejection fraction (45.2 ± 12.8) and a higher number of diseased vessels (2.9 ± 0.4) compared to those in the MI-CABG (57.3 ± 7.0; 2.0 ± 0.6) and R-CABG (56.9 ± 4.5; 2.7 ± 0.5) groups. Between the robotic and minimally invasive techniques, ejection fraction was comparable; however, robotic procedures were more frequently performed in patients with single-vessel disease, while the minimally invasive approach was more often used in patients with multi-vessel disease, though still with a significantly lower number of vessels involved compared to the conventional group.

**Fig 2:**
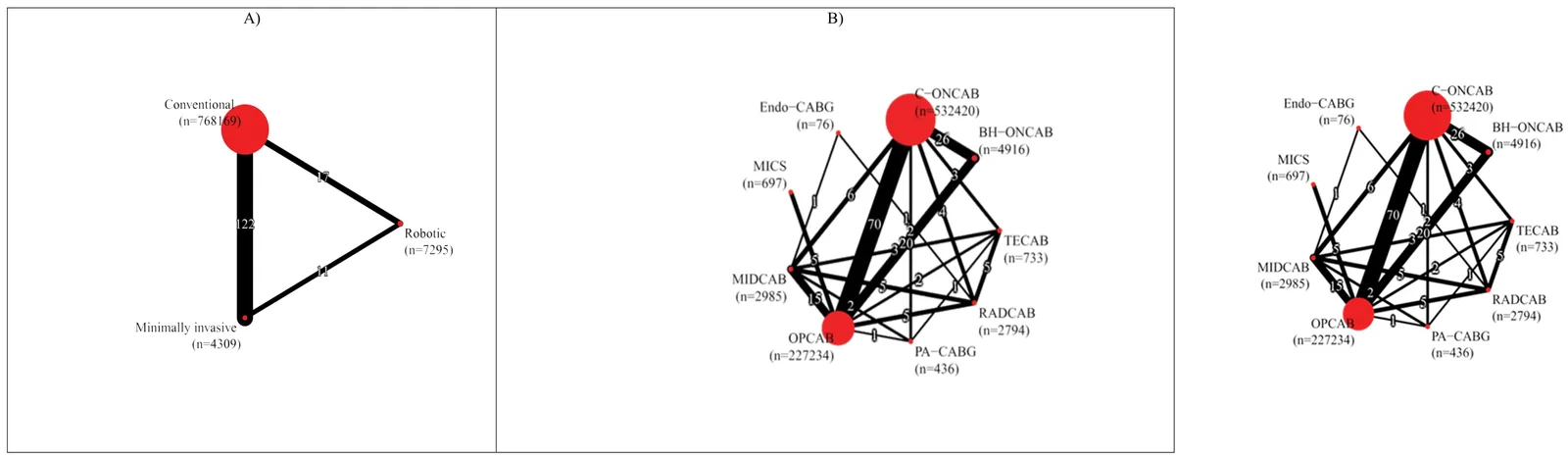
A) Netgraph of three main techniques; B) netgraph of nine sub-techniques.

### 3.2. Quality assessment

The majority of cohort studies were rated as high quality with a low risk of bias, except for two studies which were rated as fair quality. Regarding RCTs, assessment was conducted using the Cochrane Risk of Bias 2.0 tool, revealing an overall low risk of bias, except for two RCTs which raised some concerns (Appendix C in S2 Appendix [124–158], Fig S1 in S1 File).

### 3.3. Publication bias

For publication bias regarding the three main techniques, Egger’s test showed no significant bias. However, two outcomes could not be assessed due to the limited number of studies (complete revascularization and ventilation time), though the overall funnel plot showed symmetry. For the nine sub-techniques, the funnel plots were generally symmetrical and overall, the Egger’s test showed no bias except for three outcomes which are length of stay, CVA, and renal failure. For renal failure, the Egger’s test indicated statistically significant asymmetry in the funnel plot (p = 0.04). However, upon further bias testing (Begg-Mazumdar, p = 0.09; Thompson-Sharp, p = 0.08), we concluded that there was no significant publication bias for this outcome. While, for CVA and length of stay, the Egger’s test showed that there is a statistically significant asymmetry in the funnel plot (both p < 0.01). Further bias tests, showed different results. For, Thompson-sharp, both outcomes showed the similar results to Egger’s test, whereas the Begg-Mazumdar test showed insignificant results (CVA (p = 0.62) and length of stay (p = 0.30)). This might suggest that the bias is negligible and not likely to substantially affect the overall conclusions of the meta-analysis (Fig S2-3 in S1 File). Besides publication bias, our sensitivity analysis demonstrated that the majority of the outcomes showed insignificant to low heterogeneity. A few outcomes exhibited moderate to high heterogeneity, our analysis showed that the heterogeneity is likely to be caused by the high variety of the techniques (Fig S4-5 in S1 File). While for the network coherence, our node-splitting analysis showed p-values>0.05, indicating no significant differences between the direct and indirect evidence. (Fig S6-7 in S1 File)

### 3.4. Operative procedure

A total of 7,716 patients were included for the operative time outcomes (Fig 3A, B, Appendix B.2 in S2 Appendix [24–158]). In the analysis of the three main techniques and nine sub-techniques analysis, there was no significant difference in operative time among them (Table 1 and 2, Supp table 1A in S1 File). The GRADE certainty of evidence was very low (C-CABG vs others) and moderate (MI-CABG vs R-CABG). (Table 2, Supp table 2A in S1 File). All details about the GRADE evaluation are located in Supp. Table 3-4 in S1 File.

**Table 1:** Ranking of three main (overall) Techniques’ outcomes (P-score) along with heterogeneity, egger’s test, and node-splitting value.

Technique	Outcome (rank (p-score))											
	Operative time	ICU stay	Length of stay	Mortality	Complete revascularization	CVA	MI	Non-SR	Reoperation	Ventilation time	Wound infection	Renal failure
R-CABG	1 (0.77)	1 (1.00)	1 (0.97)	1 (0.89)	1 (0.87)	1 (0.90)	1 (0.72)	1 (0.89)	1 (0.78)	1 (0.93)	1 (1.00)	1 (0.90)
MI-CABG	1 (0.59)	2 (0.50)	2 (0.53)	2 (0.61)	1 (0.51)	1 (0.49)	1 (0.55)	1 (0.61)	2 (0.00)	2 (0.57)	2 (0.48)	2 (0.58)
C-CABG	1 (0.14)	3 (0.00)	3 (0.00)	3 (0.00)	1 (0.12)	2 (0.16)	1 (0.23)	2 (0.00)	1 (0.72)	3 (0.00)	2 (0.02)	2 (0.02)
Heterogeneity	99.78%	97.91%	99.09%	<0.05%	<0.05%	<0.05%	<0.05%	<0.05%	<0.05%	91.73%	<0.05%	<0.05%
Node-splitting	Consistent	Consistent	Consistent	Consistent	Consistent	Consistent	Consistent	Consistent	Consistent	Consistent	Consistent	Consistent
Egger’s test	0.97	0.30	0.19	0.76	–	0.65	0.84	0.28	0.59	0.07	0.69	0.10

Same rank mean the outcomes are comparable between the techniques, the significancy were evaluated by FDR adjusted p-values. A higher ranking indicated fewer complications and shorter durations.

**Table 2:** Ranking of nine sub-techniques’ outcomes (p-score) along with heterogeneity, egger’s test, and node-splitting value.

Technique	Outcome (rank (p-score))											
	Operative time	ICU stay	Length of stay	Mortality	Complete revascularization	CVA	MI	Non-SR	Reoperation	Ventilation time	Wound infection	Renal failure
RADCAB	4 (0.40)	1 (0.94)	3 (0.64)*	1 (0.65)	7 (0.25)	5 (0.57)	3 (0.58)	1 (0.91)	2 (0.75)	3 (0.64)*	1 (0.85)	1 (0.80)
TECAB	6 (0.33)	3 (0.71)*	1 (0.94)*	4 (0.50)	2 (0.69)	2 (0.70)	7 (0.21)	9 (0.12)*	9 (0.10)	4 (0.47)*	4 (0.61)	3 (0.73)
MIDCAB	1 (0.88)	4 (0.57)*	6 (0.50)*	3 (0.62)	4 (0.62)	3 (0.64)	6 (0.50	3 (0.66)	7 (0.32)	2 (0.83)*	6 (0.38)	2 (0.78)
MICS	9 (0.26)	2 (0.82)	5 (0.52)*	1 (0.65)	–	1 (0.78)	5 (0.52)	2 (0.69)	1 (0.77)	3 (0.64)	2 (0.82)	5 (0.45)
PA-CABG	8 (0.29)	5 (0.43)*	2 (0.90)*	6 (0.42)	5 (0.55)*	8 (0.15)	8 (0.10)*	4 (0.61)*	8 (0.28)	1 (0.94)*	5 (0.56)	–
Endo-CABG	2 (0.87)	–	4 (0.63)*	2 (0.64)	1 (0.70)		–	5 (0.58)	4 (0.62)	–	3 (0.76)	4 (0.53)
C-ONCAB	5 (0.33)	8 (0.01)	9 (0.01)	8 (0.20)	8 (0.17)	6 (0.34)	4 (0.51)	7 (0.23)	5 (0.53)	6 (0.11)	9 (0.09)	7 (0.18)
BH-ONCAB	4 (0.40)	7 (0.15)	8 (0.12)	7 (0.35)	6 (0.40)	7 (0.18)	1 (0.81)	8 (0.17)	6 (0.40)	7 (0.03)	8 (0.11)	8 (0.10)
OPCAB	3 (0.75)	6 (0.36)*	7 (0.25)*	5 (0.46)	3 (0.63)	4 (0.63)	2 (0.77)	6 (0.53)*	3 (0.73)	5 (0.33)*	7 (0.32)	6 (0.42)
Heterogeneity	99.45%	97.80%	97.99%	90.96%	69.87%	<0.05%	4.12%	58.83%	59.57%	<0.05%	<0.05%	86.19%
Node-splitting	Consistent	Consistent	Consistent	Consistent	Consistent	Consistent	Consistent	Consistent	Consistent	Consistent	Consistent	Consistent
Egger’s test	0.65	0.34	<0.05*	0.11	0.86	<0.05*	0.73	0.05	0.17	0.40	0.41	<0.05*

*: Significance with the reference (C-ONCAB) evaluated by FDR adjusted p-values

A higher ranking indicated fewer complications and shorter durations

**Fig 3:**
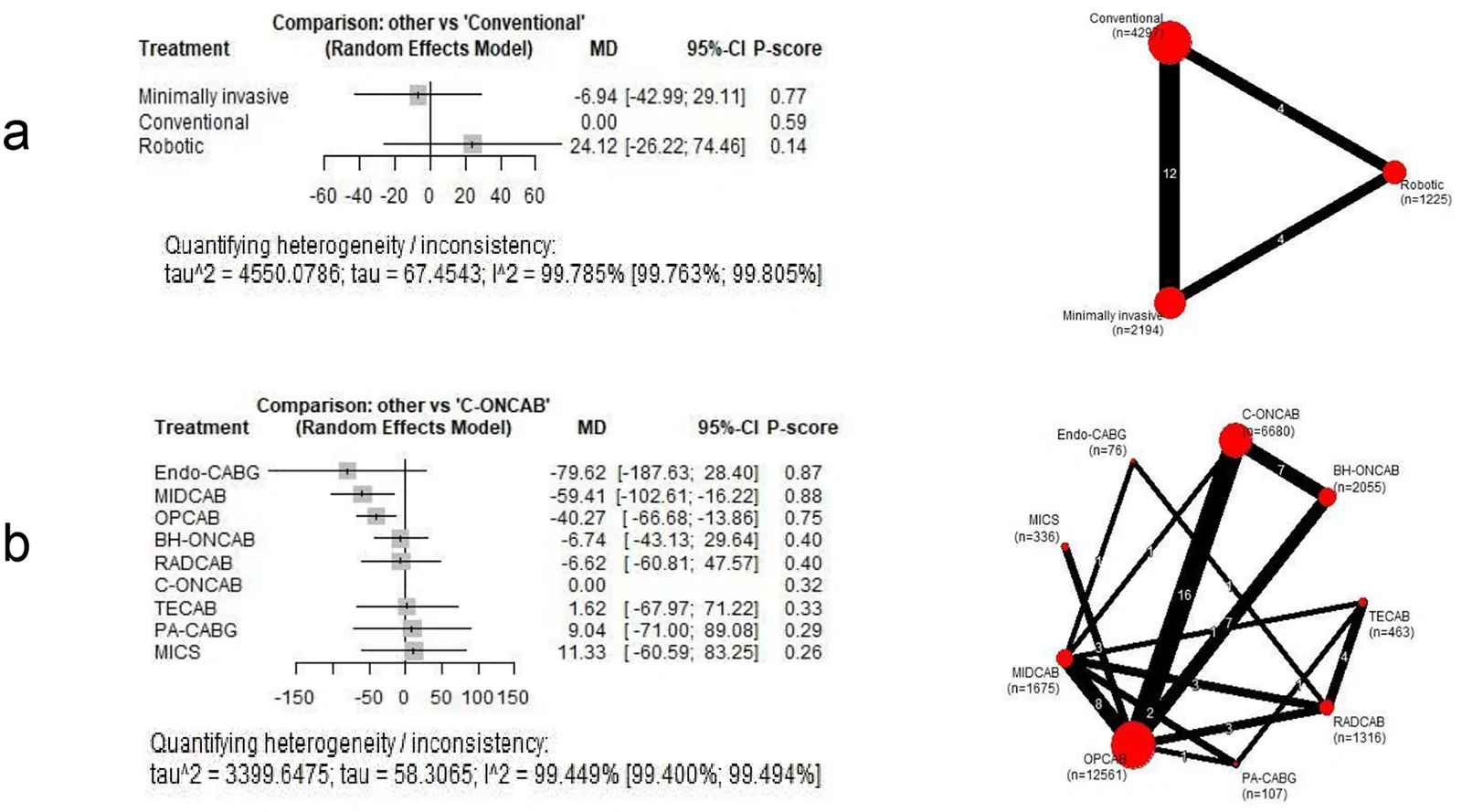
A) Operative time rank of three main techniques; b) operative time rank of nine sub-techniques.

### 3.5. Hospital stay

In terms of postoperative ICU stay, there were 9,924 patients included (Fig 4A, Appendix B.3 in S2 Appendix [24–158]). For the overall hospital stay, there were 499,627 patients (Fig 5A, Appendix B.3 in S2 Appendix).

**Fig 4:**
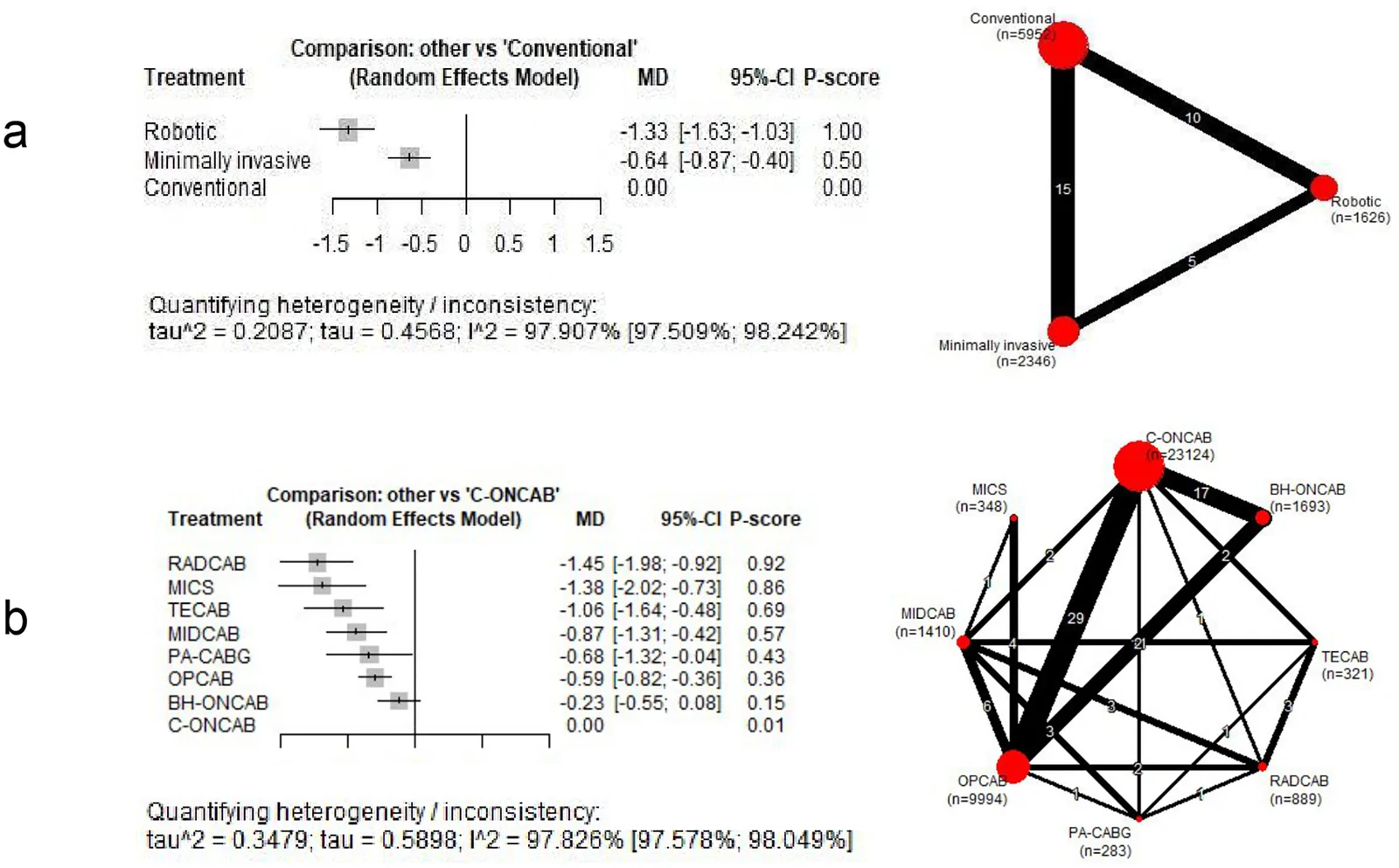
A) ICU stay rank of three main techniques; b) ICU stay rank of nine sub-techniques.

**Fig 5:**
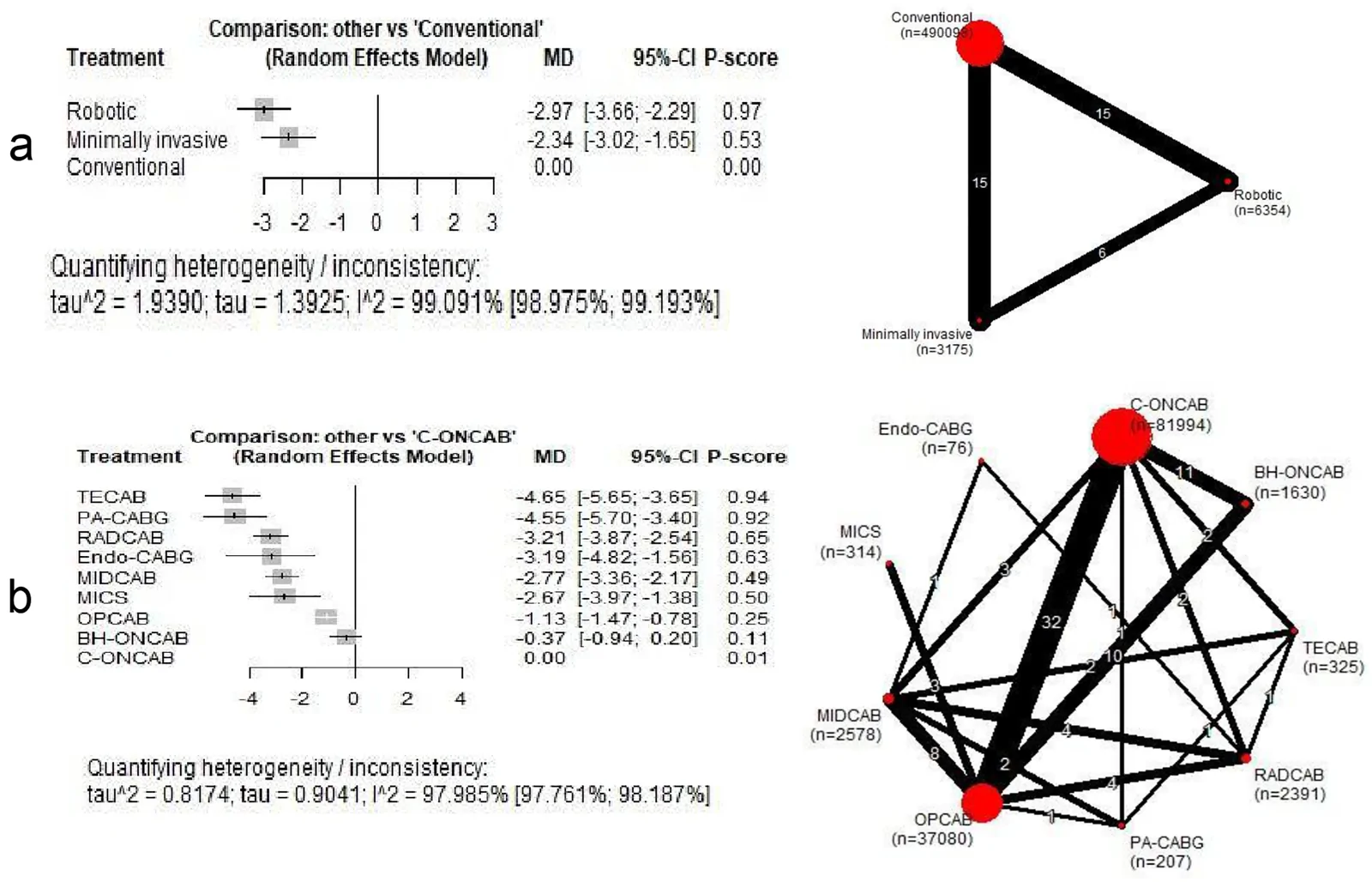
A) Hospital stay rank of three main techniques; B) Hospital stay rank of nine sub-techniques.

In terms of ICU stay, R-CABG was associated with the shortest stay [p-score: R-CABG (1.00)>MI-CABG (0.50)>C-CABG (0.00)], with a low GRADE certainty of evidence. While in the nine sub-techniques, TECAB, MIDCAB, and PA-CABG were associated with a significantly shorter stay than C-ONCAB (8 (0.01), GRADE certainty of evidence moderate, the detailed ranking can be seen in Table 1 and Table 2, Fig 4A, B, and Supp table 1-2B in S1 File. Similar results were found for overall length of stay in the hospital [p-score: R-CABG (0.97)>MI-CABG (0.53)>C-CABG (0.00)], with a low GRADE certainty of evidence. For the nine sub-techniques, almost all techniques were associated with a significantly shorter stay compared to C-ONCAB except for BH-ONCAB. The rank (from shortest stay) is: TECAB (1(0.94))> PA-CABG (2(0.90))> RADCAB (3 (0.64))> Endo-CABG (4 (0.63))> MICS (5 (0.52))> MIDCAB (6 (0.50))> OPCAB (7 (0.25))> BH-ONCAB (8 (0.12)) ~ C-ONCAB (9 (0.01)) (Table 1 and Table 2, Fig 5A, B, and Supp table 1-2C in S1 File), with a low GRADE certainty of evidence. All details about the GRADE evaluation are located in Supp. Table 5-8 in S1 File.

### 3.6. Postoperative outcome

The nine domains of postoperative outcomes are located in Table 1 and Table 2. Appendix B.4 in S2 Appendix [24–158]. All details about the GRADE evaluation are located in Supp. Table 9-26 in S1 File.

#### 3.6.1. Primary outcomes: mortality and complete revascularization.

In the comparison of the three main techniques, R-CABG was associated with the lowest mortality compared to C-CABG [p-score: R-CABG (0.89)>MI-CABG (0.61)>C-CABG (0.00)], with a moderate GRADE certainty of evidence. For the nine sub-techniques the results were comparable (Fig 6 and Fig 7, Supp. Table 1&2D in S1 File), also with a GRADE certainty of evidence. For complete revascularization rate, comparison of three main techniques showed similar rate of complete revascularization, GRADE certainty of evidence moderate. While for the nine sub-techniques only PA-CABG was associated with a significantly higher rate compared to C-ONCAB [p-SCORE: PA-CABG (0.55) > C-ONCAB (0.17)] (Table 2-3,5 in S1 File, Fig 5B, Supp. Table 1&2E in S1 File), with moderate GRADE certainty of evidence.

**Fig 6:**
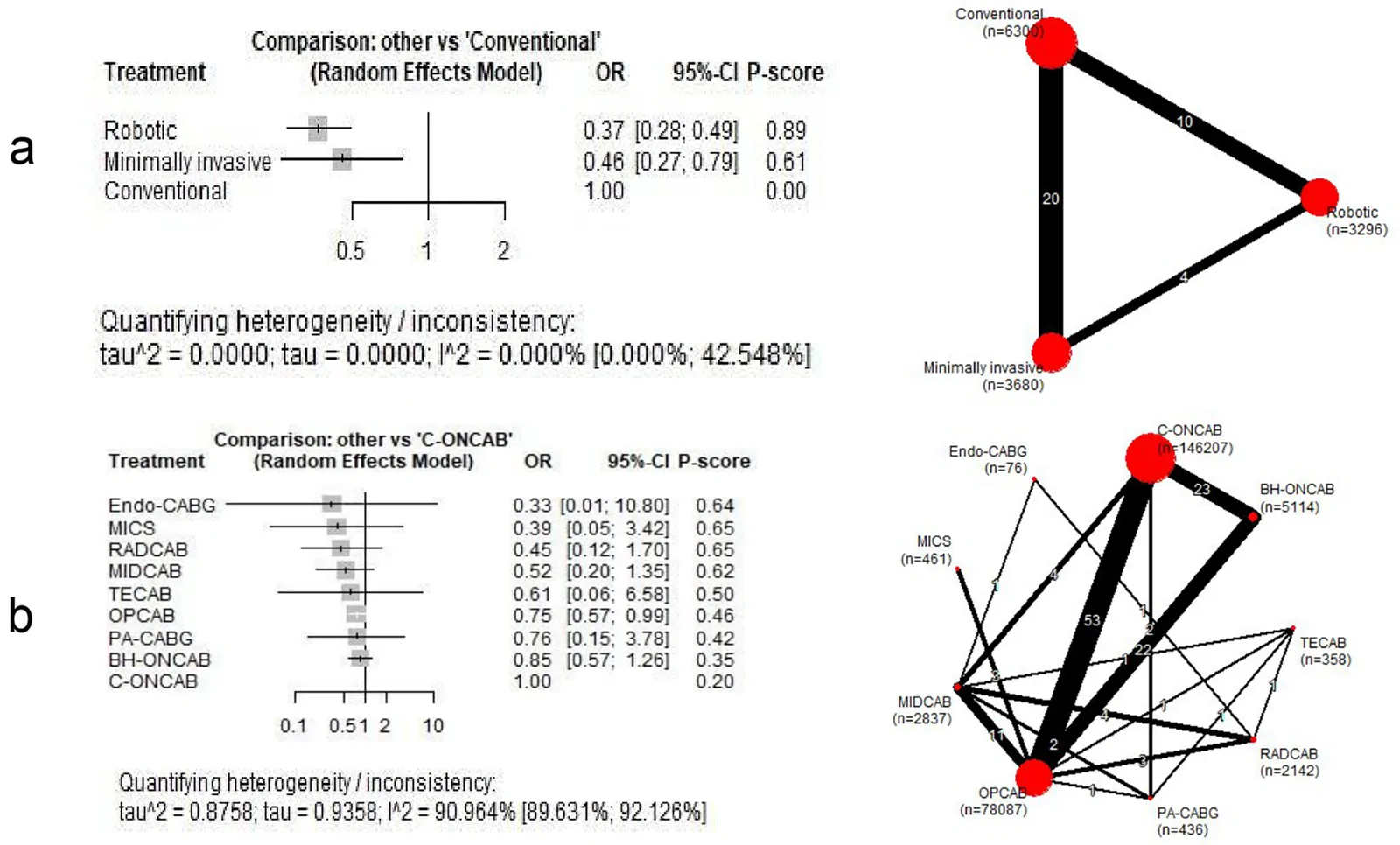
A) Mortality rank of three main techniques; B) Mortality rank of nine sub-techniques.

**Fig 7:**
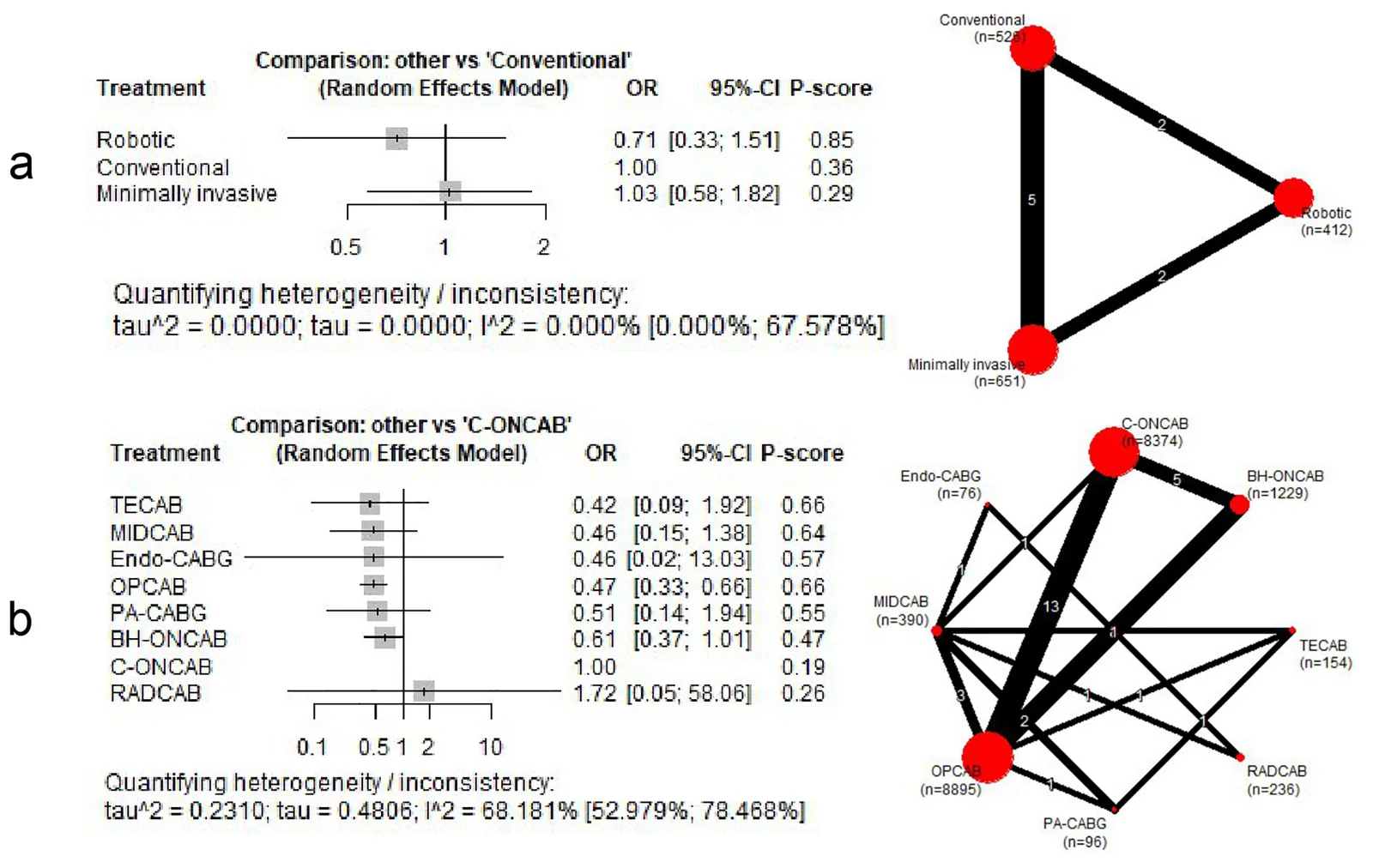
A) Complete revascularization rank of three main techniques; B) Complete revascularization rank of nine sub-techniques.

#### 3.6.2. Secondary outcomes.

R-CABG was associated with the shortest ventilation time [p-score: R-CABG (0.93)>MI-CABG (0.57)>C-CABG (0.00)], with a low GRADE certainty of evidence. R-CABG and MI-CABG were associated with lower non-SR (both with moderate GRADE certainty of evidence). Furthermore, R-CABG was related to lower rates of wound infection, CVA, and renal failure than C-CABG, both GRADE certainty of evidence moderate. However, MI-CABG was associated with the highest reoperation rate (Table 2 and Supp table 1F-L in S1 File), GRADE certainty of evidence moderate. Nevertheless, when the analysis of the nine sub-techniques was performed, all techniques had a similar reoperation rate compared to C-ONCAB (Table 3 in S1 File), GRADE certainty of evidence low to moderate.

In terms of other postoperative outcomes such as non SR, PA-CABG (p-score: 0.61) was associated with a lower rate compared to C-ONCAB (p-score: 0.23), with moderate GRADE certainty of evidence. For ventilation time, all techniques were associated with a significantly shorter time than C-ONCAB, except for BH-ONCAB. [p-score: PA-CABG (1(0.94))> MIDCAB (2(0.83))> MICS (3(0.64))> TECAB (4(0.47))> OPCAB (5(0.33))> C-ONCAB (6(0.11)) ~ BH-ONCAB (7(0.03))] (Table 3 and Supp table 2F-L in S1 File), GRADE certainty of evidence very low to moderate (except for MICS and PA-CABG, its significance is only based on indirect evidence). While in the rest of outcomes, all subtechniques showed comparable outcomes (Fig S8-9 in S1 File)).

## 4. Discussion

Our NMA results demonstrated that the three main techniques and nine sub-techniques showed comparable operative times. This finding differs from the systematic review conducted by Hammal et al., which highlighted that MIDCAB takes longer than RADCAB and has a shorter operative time compared to C-ONCAB [159]. However, that study was only a systematic review using a range of times, offering a lower quality of evidence compared to our NMA. Other studies by Lichtenberg et al. and Sabashnikov et al. provided different results, stating that MIDCAB has a shorter operative time compared to C-ONCAB [160,161]. Conflicting findings mainly result from significant differences in the number of diseased vessels and patient risk profiles across studies [161–167].

Furthermore, the extent of revascularization is a critical determinant of long-term graft patency and clinical success. Our NMA demonstrated that the three primary techniques (R-CABG, MI-CABG, and C-CABG) achieved comparable rates of complete revascularization, suggesting that robotic and minimally invasive approaches are as effective as the conventional strategy. Interestingly, the sub-technique analysis showed that PA-CABG achieved significantly higher rates of complete revascularization compared to C-ONCAB. This favorable outcome in the PA-CABG group may be attributable to several factors, including the better visualization inherent in endoscopic platforms which facilitates a higher quality of graft harvesting with minimal vessel injury. However, it is crucial to consider that baseline differences may bias these results in favor of less invasive techniques. Patients undergoing C-CABG in the included studies consistently presented with a higher number of diseased vessels and lower left ventricular ejection fraction (LVEF) compared to those in the MI-CABG and R-CABG groups. This suggests that surgeons may preferentially select “healthier” patients with simpler anatomy for less invasive approaches, while reserving C-CABG for more complex cases. Additionally, unmeasured confounders, such as higher surgeon volume and institutional expertise in specialized centers performing PA-CABG or overall MI-CABG, likely contribute to these superior outcomes. Therefore, while our findings suggest a potential technical advantage for PA-CABG, they should be interpreted with caution given the small number of comparative studies available and the inherent selection bias regarding patient baseline characteristics [161–167].

Regarding ICU stay, the shortest stays were observed in the following order: R-CABG, followed by MI-CABG, and then C-CABG. In congruence with the meta-analysis by Hammal et al., the ICU stay for R-CABG was shown to be shorter than that for C-CABG [159]. Further analysis of the sub-techniques indicated that only TECAB, MIDCAB, and PA-CABG had significantly shorter ICU stays than C-ONCAB. Another study by Yu et al. supports our findings, showing that OPCAB has a shorter ICU stay than C-ONCAB [168]. A study by Mizutani et al. also supports our results, reporting that BH-ONCAB is more efficient than C-ONCAB [169]. Various other studies further supported our findings, showing that both BH-ONCAB and OPCAB had shorter ICU stays than C-ONCAB [170–172]. Our analysis showed that the ranking for overall length of stay was similar to the ICU stay results. Hammal et al.’s study also showed that R-CABG and MI-CABG are significantly faster in terms of recovery than C-CABG. These two techniques have fewer complications, which likely contribute to the shorter ICU and overall lengths of stay [159]. Further analysis of the nine sub-techniques showed that almost all had significantly shorter stays compared to C-ONCAB, except for BH-ONCAB. Notably, TECAB had the shortest length of stay, followed by PA-CABG. This result aligns with the study by Jegaden et al., 2011, which showed that TECAB had a shorter length of stay compared to PA-CABG [166].

Our study found that R-CABG was associated with the lowest mortality and ventilation time, along with lower rates of wound infection, CVA, and renal failure compared to C-CABG. Additionally, both R-CABG and MI-CABG were associated with lower rates of non-SR than C-CABG. These findings are largely consistent with the existing literature. A meta-analysis by Hammal et al. reported similar outcomes, demonstrating that both R-CABG and MI-CABG were associated with lower mortality, shorter ventilation times, and reduced renal failure compared to C-CABG [159]. Likewise, Lin et al. supported our findings by showing a lower mortality rate for R-CABG than for C-CABG [42], while Detter et al. also reported lower mortality in the MI-CABG group relative to C-CABG. Furthermore, the study by Leyvi et al. revealed a significantly lower incidence of non-SR in R-CABG patients than in those undergoing C-CABG [173], reinforcing our own observations. The same study, along with the one by Teman et al., also supported our results regarding reduced renal failure in R-CABG and MI-CABG groups [173,174].In terms of overall superiority, the study by Watson et al. concluded that MI-CABG was generally superior to C-CABG, in line with our findings.

While many outcomes show that R-CABG and MI-CABG are comparable, our analysis suggests that R-CABG may offer additional benefits in specific areas particularly in reducing wound infection and CVA. Notably, R-CABG ranked highest in minimizing wound infection across all major techniques. This aligns with Hammal et al., who also found a significant reduction in wound infections with R-CABG compared to C-CABG [159]. Similarly, studies by Cavallaro et al. and Sabashnikov et al. highlighted the promising outcomes of R-CABG in minimizing CVA rates when compared to other CABG techniques [159,175]. Additional support for the neurological benefits of R-CABG comes from research by Kofler et al. and Sabashnikov et al., both of whom reported reduced CVA incidence in the R-CABG group [155,173].

### 4.1. Techniques challenges

Although R-CABG offers promising advantages over C-CABG regarding several complications, the technique is significantly more complex and demands a high level of surgical expertise. Proficiency in these procedures requires a structured transition period; for instance, Papakonstantinou et al. emphasize that the favorable outcomes of RADCAB are heavily dependent on the surgeon’s skill and experience [3]. Specifically, the learning curve for R-CABG is initially steep, with studies by Patrick et al. quantifying that surgeons require at least 10–20 cases to achieve technical stabilization and proficiency [176]. As experience grows, the learning curve flattens, leading to gradually improved operative times and patient outcomes. This underscores the requisite period of adjustment and the crucial role of surgical mentorship, continuous training, and professional development when adopting robotic platforms. Consequently, while the benefits of R-CABG are evident, the initial phase of adoption requires careful patient selection to mitigate risks associated with the surgeon’s learning trajectory [3,176].

### 4.2. Limitations

First, this NMA predominantly included cohort studies, with very few available RCTs due to the limited number of trials in the field. The possibility of confounding remains, as two potential effect modifiers, LVEF and the number of diseased vessels,which differed significantly between techniques; this may influence the outcomes. Because these variables are also plausible determinants of treatment selection and prognosis, their imbalance across techniques may violate the transitivity assumption underlying the network comparisons; where a technique was preferentially used in patients with better baseline LVEF or less extensive disease, its apparent advantage in treatment estimates and P-score ranking could partly reflect this case-mix imbalance rather than a true treatment effect, and rankings should therefore be interpreted as hypothesis-generating rather than definitive evidence of superiority. Furthermore, unmeasured confounders such as surgeon-specific volume and institutional expertise were not accounted for, which could disproportionately favor more advanced techniques like R-CABG and MI-CABG performed in high-volume specialized centers. Second, wide confidence intervals were observed in some outcomes, likely due to clinical and methodological heterogeneity across studies, despite the application of random-effects models. Much of this variability stemmed from the considerable differences in surgical techniques, as demonstrated by our sensitivity analyses. For several outcomes, heterogeneity was very high (I^2^ > 90%), most likely reflecting differences across studies in patient case-mix, surgical technique and operator experience, follow-up duration, and outcome ascertainment; such high heterogeneity widens the uncertainty around the pooled estimates and reduces confidence in the corresponding treatment rankings, so these particular outcomes should be interpreted with additional caution and viewed as exploratory. In addition, this NMA combined studies that reported outcomes based on either the intention-to-treat (ITT) or as-treated analysis principles due to the different study designs included (RCTs and observational studies) which can further increase heterogeneity. Because as-treated analyses can favor the received treatment by excluding crossovers or non-adherent patients, whereas ITT analyses preserve randomization but may dilute effect estimates, pooling both approaches could bias effect estimates in either direction and add a further source of between-study heterogeneity that is not fully captured by I^2^ alone. It should also be noted that one study by Cavallaro et al. (2015) contributed nearly 50% of the total patient population. While this large sample size increases statistical power, it may disproportionately influence pooled effect estimates. Sensitivity analyses excluding this study confirmed the robustness of our findings; nonetheless, this potential source of bias should be taken into account when interpreting the results. Taken together, these limitations suggest that while the results can serve as exploratory and hypothesis-generating, they should be interpreted with caution. In particular, because the network was dominated by observational studies subject to confounding by indication, P-scores and the resulting treatment rankings should not be read as evidence of one technique’s superiority over another; they are better regarded as a summary of the relative ordering of effect estimates within this evidence base, to be confirmed by future randomized trials. Further, more definitive conclusions will require future NMAs based exclusively on RCT data.

## 5. Conclusion

R-CABG suggests promising advantages over C-CABG. However, given the inherent limitations of the included studies and the nature of indirect comparisons, these findings should be regarded as hypothesis-generating rather than definitive. While providing a valuable framework for surgical decision-making, these insights must be integrated with institutional expertise and patient-specific profiles. Ultimately, large-scale prospective RCTs are imperative to validate these results and bridge current knowledge gaps in CABG revascularization strategies.

## Supporting information

S1 Appendix A. Results of databases or platforms search. (DOCX)

S2 Appendix Link to Appendix B-C. (PDF)

S1 File Supplementary tables for S1-S26, Figs 1-9. (ZIP)

S2 File PRISMA NMA checklist of items to include when reporting a systematic review involving a network meta-analysis. (DOCX)

## Abbreviation:

CABG: Coronary artery bypass grafting
CHD: Coronary heart disease
C-CABG: Conventional coronary artery bypass grafting
MI-CABG: Minimally invasive coronary artery bypass grafting
R-CABG: Robotic coronary artery bypass grafting
RADCAB: Robotic-assisted direct coronary artery bypass
TECAB: Totally endoscopic coronary artery bypass
MIDCAB: Minimally invasive direct coronary artery bypass
MICS: Minimally invasive cardiac surgery
PA-CABG: Port-access coronary artery bypass grafting
Endo-CABG: Endoscopic; coronary artery bypass grafting
C-ONCAB: Conventional on-pump coronary artery bypass
BH-ONCAB: Beating-heart coronary artery bypass
OPCAB: Off-pump coronary artery bypass
MI: Myocardial infarction
SR: Sinus rhythm
CVA: Cerebrovascular accidents
