# Supplementary material for: Clinical outcomes in various coronary artery bypass grafting techniques: A systematic review and network meta-analysis
Source: PLoS One. 2026 Nov 5;21(11):e0360757. doi: 10.1371/journal.pone.0360757 (PMC13739143; doi:10.1371/journal.pone.0360757)
Supplement: Supplementary file 1 [file pone.0360757.s002.pdf]

Link to Appendix B-C:

https://docs.google.com/spreadsheets/d/1c4qZZA9QZrxyk8qJBu9l_c0poS4nuk4G/edit?usp=sharing&ouid=115506432859215879911&rtpof=true&sd=true
