# Supplementary material for: Clinical outcomes in various coronary artery bypass grafting techniques: A systematic review and network meta-analysis
Source: PLoS One. 2026 Nov 5;21(11):e0360757. doi: 10.1371/journal.pone.0360757 (PMC13739143; doi:10.1371/journal.pone.0360757)
Supplement: Supplementary file 2 [file pone.0360757.s001.docx]

**Appendix A.** Results of databases or platforms search

| **Database or platform** | **Keyword(s)** | **Hits** |
| --- | --- | --- |
| Medline | ((Coronary Artery Bypass[MeSH]) OR (CABG) OR (coronary artery bypass graft surgery) OR (coronary artery bypass surgery) OR (bypass surgery) OR (bypass cardiac surgery)) AND ((Robotic Surgical Procedures[MeSH]) OR (Minimally Invasive Surgical Procedures[MeSH]) OR (Thoracic Surgery, Video-Assisted[MeSH]) OR (Sternotomy[MeSH]) OR (robotic surgery) OR (da vinci robotic system) OR (robot surgery) OR (RCAB) OR (RCABG) OR (robot-assisted coronary artery bypass) OR (robot-assisted coronary artery bypass surgery) OR (robot-assisted coronary artery bypass graft surgery) OR (minimally invasive) OR (minithoracotomy) OR (thoracoscopic) OR (nonsternotomy) OR (ministernotomy) OR (minimally invasive direct coronary artery bypass) OR (MIDCAB) OR (MIDCABG) OR (minimally invasive cardiac surgery) OR (MICS) OR (MICS CABG) OR (video assisted cardiac surgery) OR (video assisted thoracoscopic surgery) OR (VATS) OR (VATS CABG) OR (port-access bypass surgery) OR (PA-CAB) OR (PACAB) OR (PA-CABG) OR (PCABG) OR (port-access coronary artery bypass) OR (port-access coronary artery bypass surgery) OR (port-access coronary artery bypass graft surgery) OR (thoracoscopic cardiac surgery) OR (totally endoscopic bypass surgery) OR (totally endoscopic coronary artery bypass surgery) OR (endoscopic CABG) OR (Endo-CABG) OR (Endo CABG) OR (TECAB) OR (TECABG) OR (conventional) OR (C-CABG) OR (sternotomy) OR (middle sternotomy) OR (median sternotomy) OR (ONCAB) OR (OPCAB) OR (beating heart CABG) OR (BH CABG))  **Filters applied:** Full text, Clinical Study, Clinical Trial, Comparative Study, Controlled Clinical Trial, Multicenter Study, Observational Study, Randomized Controlled Trial, Human, Adult | 7,281 |
| Google Scholar | ((CABG) OR (coronary artery bypass graft surgery)  OR (coronary artery bypass surgery) OR (bypass surgery) OR (bypass cardiac surgery)) AND ((robotic surgery) OR (da vinci robotic system) OR (robot surgery) OR (RCAB) OR (RCABG) OR (robot-assisted coronary artery bypass) OR (robot-assisted coronary artery bypass surgery) OR (robot-assisted coronary artery bypass graft surgery) OR (minimally invasive) OR (minithoracotomy) OR (thoracoscopic) OR (nonsternotomy) OR (ministernotomy) OR (minimally invasive direct coronary artery bypass) OR (MIDCAB) OR (MIDCABG) OR (minimally invasive cardiac surgery) OR (MICS) OR (MICS CABG) OR (video assisted cardiac surgery) OR (video assisted thorascopic surgery) OR (VATS) OR (VATS CABG) OR (port-access bypass surgery) OR (PA-CAB) OR (PACAB) OR (PA-CABG) OR (PCABG) OR (port-access coronary artery bypass) OR (port-access coronary artery bypass surgery) OR (port-access coronary artery bypass graft surgery) OR (thorascopic cardiac surgery) OR (totally endoscopic bypass surgery) OR (totally endoscopic coronary artery bypass surgery) OR (endoscopic CABG) OR (Endo-CABG) OR (Endo CABG) OR (TECAB) OR (TECABG) OR (conventional) OR (C-CABG) OR (sternotomy) OR (middle sternotomy) OR (median sternotomy) OR (ONCAB) OR (OPCAB) OR (beating heart CABG) OR (BH CABG) | 4,210 |
| Cochrane | 3063 Trials matching ((CABG) OR (coronary artery bypass graft surgery) OR (coronary artery bypass surgery) OR (bypass surgery) OR (bypass cardiac surgery)) in Title Abstract Keyword AND ((robotic surgery) OR (da vinci robotic system) OR (robot surgery) OR (RCAB) OR (RCABG) OR (robot-assisted coronary artery bypass) OR (robot-assisted coronary artery bypass surgery) OR (robot-assisted coronary artery bypass graft surgery) OR (minimally invasive) OR (minithoracotomy) OR (thoracoscopic) OR (nonsternotomy) OR (ministernotomy) OR (minimally invasive direct coronary artery bypass) OR (MIDCAB) OR (MIDCABG) OR (minimally invasive cardiac surgery) OR (MICS) OR (MICS CABG) OR (video assisted cardiac surgery) OR (video assisted thorascopic surgery) OR (VATS) OR (VATS CABG) OR (port-access bypass surgery) OR (PA-CAB) OR (PACAB) OR (PA-CABG) OR (PCABG) OR (port-access coronary artery bypass) OR (port-access coronary artery bypass surgery) OR (port-access coronary artery bypass graft surgery) OR (thorascopic cardiac surgery) OR (totally endoscopic bypass surgery) OR (totally endoscopic coronary artery bypass surgery) OR (endoscopic CABG) OR (Endo-CABG) OR (Endo CABG) OR (TECAB) OR (TECABG) OR (conventional) OR (C-CABG) OR (sternotomy) OR (middle sternotomy) OR (median sternotomy) OR (ONCAB) OR (OPCAB) OR (beating heart CABG) OR (BH CABG)) in Title Abstract Keyword - (Word variations have been searched) | 3,063 |
| Scopus | ( ( cabg ) OR ( coronary AND artery AND bypass AND graft AND surgery ) OR ( coronary AND artery AND bypass AND surgery ) OR ( bypass AND surgery ) OR ( bypass AND cardiac AND surgery ) ) AND ( ( robotic AND surgery ) OR ( da AND vinci AND robotic AND system ) OR ( robot AND surgery ) OR ( rcab ) OR ( rcabg ) OR ( robot-assisted AND coronary AND artery AND bypass ) OR ( robot-assisted AND coronary AND artery AND bypass AND surgery ) OR ( robot-assisted AND coronary AND artery AND bypass AND graft AND surgery ) ) AND ( ( minimally AND invasive ) OR ( minithoracostomy ) OR ( thoracoscopic ) OR ( nonsternotomy ) OR ( ministernotomy ) OR ( minimally AND invasive AND direct AND coronary AND artery AND bypass ) OR ( midcab ) OR ( midcabg ) OR ( mics ) OR ( mics AND cabg ) ) OR ( ( port-access AND bypass AND surgery ) OR ( pa-cab ) OR ( pacab ) OR ( pa-cabg ) OR ( pcabg ) OR ( port-access AND coronary AND artery AND bypass ) OR ( port-access AND coronary AND artery AND bypass AND surgery ) OR ( port-access AND coronary AND artery AND bypass AND graft AND surgery ) ) OR ( ( totally AND endoscopic AND bypass AND surgery ) OR ( totally AND endoscopic AND coronary AND artery AND bypass AND surgery ) OR ( endoscopic AND cabg ) OR ( endo-cabg ) OR ( endo AND cabg ) OR ( tecab ) OR ( tecabg ) OR ( conventional ) OR ( c-cabg ) OR ( sternotomy ) OR ( middle AND sternotomy ) OR ( median AND sternotomy ) ) AND ( LIMIT-TO ( SUBJAREA , "MEDI" ) OR EXCLUDE ( SUBJAREA , "ENGI" ) OR EXCLUDE ( SUBJAREA , "PHYS" ) OR EXCLUDE ( SUBJAREA , "MATE" ) OR EXCLUDE ( SUBJAREA , "CENG" ) OR EXCLUDE ( SUBJAREA , "IMMU" ) OR EXCLUDE ( SUBJAREA , "ENVI" ) OR EXCLUDE ( SUBJAREA , "DENT" ) OR EXCLUDE ( SUBJAREA , "NEUR" ) OR EXCLUDE ( SUBJAREA , "PSYC" ) OR EXCLUDE ( SUBJAREA , "PHAR" ) OR EXCLUDE ( SUBJAREA , "SOCI" ) OR EXCLUDE ( SUBJAREA , "HEAL" ) OR EXCLUDE ( SUBJAREA , "BIOC" ) OR EXCLUDE ( SUBJAREA , "COMP" ) ) AND ( LIMIT-TO ( DOCTYPE , "ar" ) ) AND ( LIMIT-TO ( EXACTKEYWORD , "Human" ) OR EXCLUDE ( EXACTKEYWORD , "Gastrectomy" ) OR EXCLUDE ( EXACTKEYWORD , "Roux-en-Y Gastric Bypass" ) OR EXCLUDE ( EXACTKEYWORD , "Sleeve Gastrectomy" ) OR EXCLUDE ( EXACTKEYWORD , "Percutaneous Coronary Intervention" ) OR EXCLUDE ( EXACTKEYWORD , "Computer Assisted Surgery" ) OR EXCLUDE ( EXACTKEYWORD , "Lymph Node Dissection" ) OR EXCLUDE ( EXACTKEYWORD , "Cholecystectomy" ) OR EXCLUDE ( EXACTKEYWORD , "Systematic Review" ) OR EXCLUDE ( EXACTKEYWORD , "Quality Of Life" ) OR EXCLUDE ( EXACTKEYWORD , "Cardioplegia" ) OR EXCLUDE ( EXACTKEYWORD , "Surgery, Computer-Assisted" ) OR EXCLUDE ( EXACTKEYWORD , "Heart Valve Prosthesis Implantation" ) OR EXCLUDE ( EXACTKEYWORD , "Heart Atrium Septum Defect" ) OR EXCLUDE ( EXACTKEYWORD , "General Anesthesia" ) OR EXCLUDE ( EXACTKEYWORD , "Mitral Valve Surgery" ) OR EXCLUDE ( EXACTKEYWORD , "Human Tissue" ) OR EXCLUDE ( EXACTKEYWORD , "Cancer Staging" ) OR EXCLUDE ( EXACTKEYWORD , "Equipment Design" ) OR EXCLUDE ( EXACTKEYWORD , "Case Report" ) OR EXCLUDE ( EXACTKEYWORD , "Mitral Valve" ) OR EXCLUDE ( EXACTKEYWORD , "Diagnostic Imaging" ) OR EXCLUDE ( EXACTKEYWORD , "Mitral Valve Repair" ) OR EXCLUDE ( EXACTKEYWORD , "Mitral Valve Regurgitation" ) OR EXCLUDE ( EXACTKEYWORD , "Feasibility Study" ) OR EXCLUDE ( EXACTKEYWORD , "Pathology" ) OR EXCLUDE ( EXACTKEYWORD , "Cancer Surgery" ) OR EXCLUDE ( EXACTKEYWORD , "Artificial Ventilation" ) OR EXCLUDE ( EXACTKEYWORD , "Pneumonia" ) OR EXCLUDE ( EXACTKEYWORD , "Child" ) OR EXCLUDE ( EXACTKEYWORD , "Bariatric Surgery" ) OR EXCLUDE ( EXACTKEYWORD , "Prostatectomy" ) OR EXCLUDE ( EXACTKEYWORD , "Mitral Valve Replacement" ) OR EXCLUDE ( EXACTKEYWORD , "Videorecording" ) OR LIMIT-TO ( EXACTKEYWORD , "Review" ) ) AND ( LIMIT-TO ( SRCTYPE , "j" ) ) AND ( LIMIT-TO ( PUBSTAGE , "final" ) ) | 929 |
| Wiley | "((CABG) OR (coronary artery bypass graft surgery) OR (coronary artery bypass surgery) OR (bypass surgery) OR (bypass cardiac surgery)) AND ((robotic surgery) OR (da vinci robotic system) OR (robot surgery) OR (RCAB) OR (RCABG) OR (robot-assisted coronary artery bypass) OR (robot-assisted coronary artery bypass surgery) OR (robot-assisted coronary artery bypass graft surgery) OR (minimally invasive) OR (minithoracotomy) OR (thoracoscopic) OR (nonsternotomy) OR (ministernotomy) OR (minimally invasive direct coronary artery bypass) OR (MIDCAB) OR (MIDCABG) OR (minimally invasive cardiac surgery) OR (MICS) OR (MICS CABG) OR (video assisted cardiac surgery) OR (video assisted thorascopic surgery) OR (VATS) OR (VATS CABG) OR (port-access bypass surgery) OR (PA-CAB) OR (PACAB) OR (PA-CABG) OR (PCABG) OR (port-access coronary artery bypass) OR (port-access coronary artery bypass surgery) OR (port-access coronary artery bypass graft surgery) OR (thorascopic cardiac surgery) OR (totally endoscopic bypass surgery) OR (totally endoscopic coronary artery bypass surgery) OR (endoscopic CABG) OR (Endo-CABG) OR (Endo CABG) OR (TECAB) OR (TECABG) OR (conventional) OR (C-CABG) OR (sternotomy) OR (middle sternotomy) OR (median sternotomy) OR (ONCAB) OR (OPCAB) OR (beating heart CABG) OR (BH CABG))" in Abstract  Publication type: Journal | 2,559 |
| Embase | ((CABG) OR (coronary artery bypass graft surgery)  OR (coronary artery bypass surgery) OR (bypass surgery) OR (bypass cardiac surgery)) AND ((robotic surgery) OR (da vinci robotic system) OR (robot surgery) OR (RCAB) OR (RCABG) OR (robot-assisted coronary artery bypass) OR (robot-assisted coronary artery bypass surgery) OR (robot-assisted coronary artery bypass graft surgery) OR (minimally invasive) OR (minithoracotomy) OR (thoracoscopic) OR (nonsternotomy) OR (ministernotomy) OR (minimally invasive direct coronary artery bypass) OR (MIDCAB) OR (MIDCABG) OR (minimally invasive cardiac surgery) OR (MICS) OR (MICS CABG) OR (video assisted cardiac surgery) OR (video assisted thorascopic surgery) OR (VATS) OR (VATS CABG) OR (port-access bypass surgery) OR (PA-CAB) OR (PACAB) OR (PA-CABG) OR (PCABG) OR (port-access coronary artery bypass) OR (port-access coronary artery bypass surgery) OR (port-access coronary artery bypass graft surgery) OR (thorascopic cardiac surgery) OR (totally endoscopic bypass surgery) OR (totally endoscopic coronary artery bypass surgery) OR (endoscopic CABG) OR (Endo-CABG) OR (Endo CABG) OR (TECAB) OR (TECABG) OR (conventional) OR (C-CABG) OR (sternotomy) OR (middle sternotomy) OR (median sternotomy) OR (ONCAB) OR (OPCAB) OR (beating heart CABG) OR (BH CABG)) AND (randomized control trial)/lim AND (article)/lim) | 2,597 |
| ProQuest | ( ( cabg ) OR ( coronary AND artery AND bypass AND graft AND surgery ) OR ( coronary AND artery AND bypass AND surgery ) OR ( bypass AND surgery ) OR ( bypass AND cardiac AND surgery ) ) AND ( ( robotic AND surgery ) OR ( da AND vinci AND robotic AND system ) OR ( robot AND surgery ) OR ( rcab ) OR ( rcabg ) OR ( robot-assisted AND coronary AND artery AND bypass ) OR ( robot-assisted AND coronary AND artery AND bypass AND surgery ) OR ( robot-assisted AND coronary AND artery AND bypass AND graft AND surgery ) ) AND ( ( minimally AND invasive ) OR ( minithoracostomy ) OR ( thoracoscopic ) OR ( nonsternotomy ) OR ( ministernotomy ) OR ( minimally AND invasive AND direct AND coronary AND artery AND bypass ) OR ( midcab ) OR ( midcabg ) OR ( mics ) OR ( mics AND cabg ) ) OR ( ( port-access AND bypass AND surgery ) OR ( pa-cab ) OR ( pacab ) OR ( pa-cabg ) OR ( pcabg ) OR ( port-access AND coronary AND artery AND bypass ) OR ( port-access AND coronary AND artery AND bypass AND surgery ) OR ( port-access AND coronary AND artery AND bypass AND graft AND surgery ) ) OR ( ( totally AND endoscopic AND bypass AND surgery ) OR ( totally AND endoscopic AND coronary AND artery AND bypass AND surgery ) OR ( endoscopic AND cabg ) OR ( endo-cabg ) OR ( endo AND cabg ) OR ( tecab ) OR ( tecabg ) OR ( conventional ) OR ( c-cabg ) OR ( sternotomy ) OR ( middle AND sternotomy ) OR ( median AND sternotomy ) ) AND ( LIMIT-TO ( SUBJAREA , "MEDI" ) OR EXCLUDE ( SUBJAREA , "ENGI" ) OR EXCLUDE ( SUBJAREA , "PHYS" ) OR EXCLUDE ( SUBJAREA , "MATE" ) OR EXCLUDE ( SUBJAREA , "CENG" ) OR EXCLUDE ( SUBJAREA , "IMMU" ) OR EXCLUDE ( SUBJAREA , "ENVI" ) OR EXCLUDE ( SUBJAREA , "DENT" ) OR EXCLUDE ( SUBJAREA , "NEUR" ) OR EXCLUDE ( SUBJAREA , "PSYC" ) OR EXCLUDE ( SUBJAREA , "PHAR" ) OR EXCLUDE ( SUBJAREA , "SOCI" ) OR EXCLUDE ( SUBJAREA , "HEAL" ) OR EXCLUDE ( SUBJAREA , "BIOC" ) OR EXCLUDE ( SUBJAREA , "COMP" ) ) AND ( LIMIT-TO ( DOCTYPE , "ar" ) ) AND ( LIMIT-TO ( EXACTKEYWORD , "Human" ) OR EXCLUDE ( EXACTKEYWORD , "Gastrectomy" ) OR EXCLUDE ( EXACTKEYWORD , "Roux-en-Y Gastric Bypass" ) OR EXCLUDE ( EXACTKEYWORD , "Sleeve Gastrectomy" ) OR EXCLUDE ( EXACTKEYWORD , "Percutaneous Coronary Intervention" ) OR EXCLUDE ( EXACTKEYWORD , "Computer Assisted Surgery" ) OR EXCLUDE ( EXACTKEYWORD , "Lymph Node Dissection" ) OR EXCLUDE ( EXACTKEYWORD , "Cholecystectomy" ) OR EXCLUDE ( EXACTKEYWORD , "Systematic Review" ) OR EXCLUDE ( EXACTKEYWORD , "Quality Of Life" ) OR EXCLUDE ( EXACTKEYWORD , "Cardioplegia" ) OR EXCLUDE ( EXACTKEYWORD , "Surgery, Computer-Assisted" ) OR EXCLUDE ( EXACTKEYWORD , "Heart Valve Prosthesis Implantation" ) OR EXCLUDE ( EXACTKEYWORD , "Heart Atrium Septum Defect" ) OR EXCLUDE ( EXACTKEYWORD , "General Anesthesia" ) OR EXCLUDE ( EXACTKEYWORD , "Mitral Valve Surgery" ) OR EXCLUDE ( EXACTKEYWORD , "Human Tissue" ) OR EXCLUDE ( EXACTKEYWORD , "Cancer Staging" ) OR EXCLUDE ( EXACTKEYWORD , "Equipment Design" ) OR EXCLUDE ( EXACTKEYWORD , "Case Report" ) OR EXCLUDE ( EXACTKEYWORD , "Mitral Valve" ) OR EXCLUDE ( EXACTKEYWORD , "Diagnostic Imaging" ) OR EXCLUDE ( EXACTKEYWORD , "Mitral Valve Repair" ) OR EXCLUDE ( EXACTKEYWORD , "Mitral Valve Regurgitation" ) OR EXCLUDE ( EXACTKEYWORD , "Feasibility Study" ) OR EXCLUDE ( EXACTKEYWORD , "Pathology" ) OR EXCLUDE ( EXACTKEYWORD , "Cancer Surgery" ) OR EXCLUDE ( EXACTKEYWORD , "Artificial Ventilation" ) OR EXCLUDE ( EXACTKEYWORD , "Pneumonia" ) OR EXCLUDE ( EXACTKEYWORD , "Child" ) OR EXCLUDE ( EXACTKEYWORD , "Bariatric Surgery" ) OR EXCLUDE ( EXACTKEYWORD , "Prostatectomy" ) OR EXCLUDE ( EXACTKEYWORD , "Mitral Valve Replacement" ) OR EXCLUDE ( EXACTKEYWORD , "Videorecording" ) OR LIMIT-TO ( EXACTKEYWORD , "Review" ) ) AND ( LIMIT-TO ( SRCTYPE , "j" ) ) AND ( LIMIT-TO ( PUBSTAGE , "final" ) in title, keywords, abstract )  Publication type: scholarly journal | 3,664 |
|  | **Total** | **24,303** |
|  | **Full-text review** | **603** |
|  | **Included** | **132** |
