# Supplementary material for: Clinical outcomes in various coronary artery bypass grafting techniques: A systematic review and network meta-analysis
Source: PLoS One. 2026 Nov 5;21(11):e0360757. doi: 10.1371/journal.pone.0360757 (PMC13739143; doi:10.1371/journal.pone.0360757)
Supplement: Supplementary file 3 [file pone.0360757.s003.zip › Supplementary Figure S3.docx]

| **Outcome** | **Funnel plot** | **Egger’s test** |
| --- | --- | --- |
| **A. Operative time** |  |  |
| **B. ICU stay** |  |  |
| **C. Length of stay** |  |  |
| **D. Mortality** |  |  |
| **E. Complete revascularization** |  |  |
| **F. CVA** |  |  |
| **G. MI** |  |  |
| **H. Non-SR** |  |  |
| **I. Reoperation** |  |  |
| **J. Ventilation time** |  |  |
| **K. Wound infection** |  |  |
| **L. Renal failure** |  |  |

# Figure S3A-L. Funnel plots and Egger’s test graphs in pair-wise meta-analysis of nine sub-technique
