# Supplementary material for: Clinical outcomes in various coronary artery bypass grafting techniques: A systematic review and network meta-analysis
Source: PLoS One. 2026 Nov 5;21(11):e0360757. doi: 10.1371/journal.pone.0360757 (PMC13739143; doi:10.1371/journal.pone.0360757)
Supplement: Supplementary file 3 [file pone.0360757.s003.zip › Supplementary Figure S4.docx]

| **Outcome** | **Sensitivity analysis plot** |
| --- | --- |
| **A. Operative time** |   The heterogeneity reduced to 0% indicates that the intervention does not cause the cause of heterogeneity. Furthermore, each intervention has equal significance. |
| **B. ICU stay** | The initial I^2^ <0.05% which indicates the lack of heterogeneity across the studies included, therefore the interventions are homogeneous. |
| **C. Length of stay** |   The heterogeneity reduced to 38% indicates that the intervention does not cause the cause of heterogeneity. Furthermore, each intervention has equal significance. |
| **D. Mortality** | The initial I^2^ <0.05% which indicates the lack of heterogeneity across the studies included, therefore the interventions are homogeneous. |
| **E. Complete revascularization** | The initial I^2^ <0.05% which indicates the lack of heterogeneity across the studies included, therefore the interventions are homogeneous. |
| **F. CVA** | The initial I^2^ <0.05% which indicates the lack of heterogeneity across the studies included, therefore the interventions are homogeneous. |
| **G. MI** | The initial I^2^ <0.05% which indicates the lack of heterogeneity across the studies included, therefore the interventions are homogeneous. |
| **H. Non-SR** | The initial I^2^ <0.05% which indicates the lack of heterogeneity across the studies included, therefore the interventions are homogeneous. |
| **I. Reoperation** | The initial I^2^ <0.05% which indicates the lack of heterogeneity across the studies included, therefore the interventions are homogeneous. |
| **J. Ventilation time** |   The heterogeneity reduced to 0% indicates that the intervention does not cause the cause of heterogeneity. Furthermore, each intervention has equal significance. |
| **K. Wound infection** | The initial I^2^ <0.05% which indicates the lack of heterogeneity across the studies included, therefore the interventions are homogeneous. |
| **L. Renal failure** | The initial I^2^ <0.05% which indicates the lack of heterogeneity across the studies included, therefore the interventions are homogeneous. |

# Figure S4A-L. Sensitivity analysis of three main (overall) techniques
