# Supplementary material for: Clinical outcomes in various coronary artery bypass grafting techniques: A systematic review and network meta-analysis
Source: PLoS One. 2026 Nov 5;21(11):e0360757. doi: 10.1371/journal.pone.0360757 (PMC13739143; doi:10.1371/journal.pone.0360757)
Supplement: Supplementary file 3 [file pone.0360757.s003.zip › Supplementary Figure S7.docx]

| **Outcome** | **Node-splitting** |
| --- | --- |
| **A. Operative time** |   P-value<0.05 for disagreement between direct and indirect comparison, thus showing consistency. |
| **B. ICU stay** |   P-value<0.05 for disagreement between direct and indirect comparison, thus showing consistency. |
| **C. Length of stay** |   P-value>0.05 for the disagreement between direct and indirect comparison for between PA-CABG and C-ONCAB, MIDCAB, and OPCAB, this happened due to the limited amount of study. But, because direct evidence gave stronger evidence, the direct evidence is the one included. |
| **D. Mortality** |   Consistent |
| **E. Complete revascularization** |   Consistent |
| **F. CVA** |   Consistent |
| **G. MI** |   Consistent |
| **H. Non-SR** |   Consistent |
| **I. Reoperation** |   Consistent |
| **J. Ventilation time** |   Consistent |
| **K. Wound infection** |   Consistent |
| **L. Renal failure** |   Consistent |

# Figure S7A-L. Node-splitting of nine sub-techniques
