# Supplementary material for: Clinical outcomes in various coronary artery bypass grafting techniques: A systematic review and network meta-analysis
Source: PLoS One. 2026 Nov 5;21(11):e0360757. doi: 10.1371/journal.pone.0360757 (PMC13739143; doi:10.1371/journal.pone.0360757)
Supplement: Supplementary file 3 [file pone.0360757.s003.zip › Supplementary Table 1.docx]

# **Supp. Table 1A-N.** Ranking netleague of three main (overall) techniques

| **A. Operative time** | | |
| --- | --- | --- |
| Conventional | 5.31 [ -33.73; 44.35] | -19.38 [ -85.96; 47.21] |
| 6.94 [-29.11; 42.99] | Minimally invasive | -35.76 [-102.03; 30.52] |
| -24.12 [-74.46; 26.22] | -31.06 [-81.34; 19.22] | Robotic |
| **B. ICU stay** | | |
| Conventional | 0.45 [ 0.19; 0.70] | 1.66 [ 1.30; 2.03] |
| 0.64 [ 0.40; 0.87] | Minimally invasive | 0.21 [-0.20; 0.62] |
| 1.33 [ 1.03; 1.63] | 0.69 [ 0.39; 1.00] | Robotic |
| **C. Length of stay** | | |
| Conventional | 2.73 [ 1.95; 3.50] | 2.62 [ 1.85; 3.40] |
| 2.34 [ 1.65; 3.02] | Minimally invasive | 1.77 [ 0.62; 2.93] |
| 2.97 [ 2.29; 3.66] | 0.63 [-0.17; 1.44] | Robotic |
| **D. Mortality** | | |
| Conventional | 1.58 [0.81; 3.06] | 2.87 [2.14; 3.85] |
| 2.18 [1.27; 3.73] | Minimally invasive | 0.71 [0.29; 1.70] |
| 2.70 [2.03; 3.58] | 1.24 [0.71; 2.17] | Robotic |
| **E. Complete revascularization** | | |
| Conventional | 0.96 [0.42; 2.19] | 4.25 [1.28; 14.09] |
| 1.42 [0.69; 2.88] | Minimally invasive | 1.00 [0.50; 2.01] |
| 1.87 [0.84; 4.18] | 1.32 [0.71; 2.48] | Robotic |
| **F. CVA** | | |
| Conventional | 0.84 [0.36; 1.96] | 1.80 [1.19; 2.72] |
| 1.19 [0.58; 2.41] | Minimally invasive | 0.65 [0.18; 2.30] |
| 1.66 [1.12; 2.47] | 1.40 [0.66; 2.97] | Robotic |
| **G. MI** | | |
| Conventional | 0.96 [0.57; 1.62] | 0.92 [0.82; 1.03] |
| 0.99 [0.62; 1.60] | Minimally invasive | 0.77 [0.23; 2.57] |
| 0.92 [0.82; 1.03] | 0.93 [0.57; 1.51] | Robotic |
| **H. Non-SR** | | |
| Conventional | 1.20 [1.01; 1.44] | 1.53 [1.18; 1.98] |
| 1.21 [1.03; 1.43] | Minimally invasive | 1.19 [0.88; 1.61] |
| 1.49 [1.21; 1.84] | 1.23 [0.99; 1.53] | Robotic |
| **I. Reoperation** | | |
| Conventional | 0.68 [0.41; 1.13] | 0.96 [0.60; 1.55] |
| 0.63 [0.44; 0.92] | Minimally invasive | 1.66 [1.27; 2.16] |
| 1.03 [0.71; 1.48] | 1.63 [1.27; 2.08] | Robotic |
| **J. Ventilation time** | | |
| Conventional | 3.09 [ 2.04; 4.15] | 2.44 [ 0.85; 4.04] |
| 3.12 [ 2.17; 4.08] | Minimally invasive | -0.82 [-2.34; 0.70] |
| 2.45 [ 1.23; 3.67] | -0.67 [-1.87; 0.53] | Robotic |
| **K. Wound infection** | | |
| Conventional | 1.61 [1.01; 2.56] | 2.55 [2.08; 3.13] |
| 1.43 [0.94; 2.17] | Minimally invasive | 2.90 [1.15; 7.27] |
| 2.61 [2.14; 3.19] | 1.83 [1.17; 2.86] | Robotic |
| **L. Renal failure** | | |
| Conventional | 1.63 [0.81; 3.28] | 1.92 [1.23; 3.00] |
| 1.59 [0.96; 2.63] | Minimally invasive | 1.25 [0.70; 2.22] |
| 1.94 [1.30; 2.90] | 1.22 [0.76; 1.96] | Robotic |
