# Supplementary material for: Clinical outcomes in various coronary artery bypass grafting techniques: A systematic review and network meta-analysis
Source: PLoS One. 2026 Nov 5;21(11):e0360757. doi: 10.1371/journal.pone.0360757 (PMC13739143; doi:10.1371/journal.pone.0360757)
Supplement: Supplementary file 3 [file pone.0360757.s003.zip › Supplementary Table 10.docx]

# Supp. Table 10. The evidence findings for nine sub-techniques in mortality

| **Certainty assessment** | | | | | | | **Effect** | **Certainty** | |
| --- | --- | --- | --- | --- | --- | --- | --- | --- | --- |
| **№ of studies** | **Study design** | **Risk of bias** | **Inconsistency** | **Indirectness** | **Imprecision** | **Publication bias** | **SMD with 95% CI** |  |  |
| **BH-ONCAB vs C-ONCAB** | | | | | | | | |  |
| **21** | Cohort Prospective, Cohort Retrospective ,RCT | Serious | Not serious | Not serious | Not serious | Undetected | 0.84 [0.51;   1.38] | Moderate | |
| **BH-ONCAB vs OPCAB** | | | | | | | | |  |
| **20** | Cohort Prospective, Cohort Retrospective ,RCT | Serious | Not serious | Not serious | Not serious | Undetected | 1.25 [0.75;   2.06] | Moderate | |
| **C-ONCAB vs MIDCAB** | | | | | | | | |  |
| **2** | Cohort Prospective | Serious | Serious | Not serious | Not serious | Undetected | 0.92 [0.09;   9.65] | Low | |
| **C-ONCAB vs OPCAB** | | | | | | | | |  |
| **56** | Cohort Prospective, Cohort Retrospective ,RCT | Serious | Not serious | Not serious | Not serious | Undetected | 1.34 [1.00;   1.79] | Moderate | |
| **C-ONCAB vs PA-CABG** | | | | | | | | |  |
| **2** | Cohort Retrospective | Serious | Not serious | Not serious | Not serious | Undetected | 1.50 [0.18;  12.39] | Moderate | |
| **Endo-CABG vs RADCAB** | | | | | | | | |  |
| **1** | Cohort Retrospective | Serious | Serious | Not serious | Not serious | Undetected | 1.03 [0.03;  41.42] | Low | |
| **MICS vs OPCAB** | | | | | | | | |  |
| **2** | Cohort Prospective, Cohort Retrospective | Serious | Serious | Not serious | Not serious | Undetected | 0.52 [0.06;   4.47] | Low | |
| **MIDCAB vs OPCAB** | | | | | | | | |  |
| **8** | Cohort Prospective, Cohort Retrospective | Serious | Serious | Not serious | Not serious | Undetected | 0.67 [0.21;   2.13] | Low | |
| **MIDCAB vs PA-CABG** | | | | | | | | |  |
| **2** | Cohort Prospective, Cohort Retrospective | Serious | Not serious | Not serious | Not serious | Undetected | 0.51 [0.03;   8.61] | Moderate | |
| **MIDCAB vs RADCAB** | | | | | | | | |  |
| **4** | Cohort Retrospective | Serious | Not serious | Not serious | Not serious | Undetected | 1.09 [0.24;   4.93] | Moderate | |
| **MIDCAB vs TECAB** | | | | | | | | |  |
| **1** | Cohort Retrospective | Serious | Not serious | Not serious | Not serious | Undetected | 0.37 [0.01;  14.88] | Moderate | |
| **OPCAB vs PA-CABG** | | | | | | | | |  |
| **1** | Cohort Prospective | Serious | Not serious | Not serious | Not serious | Undetected | 0.33 [0.01;  13.58] | Moderate | |
| **OPCAB vs RADCAB** | | | | | | | | |  |
| **2** | Cohort Prospective | Serious | Serious | Not serious | Not serious | Undetected | 1.81 [0.27;  11.96] | Low | |
| **OPCAB vs TECAB** | | | | | | | | |  |
| **1** | Cohort Retrospective | Serious | Serious | Not serious | Not serious | Undetected | 4.90 [0.14; 171.96] | Low | |
| **PA-CABG vs TECAB** | | | | | | | | |  |
| **1** | Cohort Retrospective | Serious | Not serious | Not serious | Not serious | Undetected | 0.40 [0.01;  16.47] | Moderate | |
