# Supplementary material for: Clinical outcomes in various coronary artery bypass grafting techniques: A systematic review and network meta-analysis
Source: PLoS One. 2026 Nov 5;21(11):e0360757. doi: 10.1371/journal.pone.0360757 (PMC13739143; doi:10.1371/journal.pone.0360757)
Supplement: Supplementary file 3 [file pone.0360757.s003.zip › Supplementary Table 12.docx]

# Supp. Table 12. The evidence findings for nine sub-techniques in complete revascularization

| **Certainty assessment** | | | | | | | **Effect** | **Certainty** | |
| --- | --- | --- | --- | --- | --- | --- | --- | --- | --- |
| **№ of studies** | **Study design** | **Risk of bias** | **Inconsistency** | **Indirectness** | **Imprecision** | **Publication bias** | **SMD with 95% CI** |  |  |
| **BH-ONCAB vs C-ONCAB** | | | | | | | | |  |
| **5** | Cohort Prospective, Cohort Retrospective ,RCT | Serious | Not serious | Not serious | Not serious | Undetected | 0.50 [0.24;   1.03] | Moderate | |
| **BH-ONCAB vs OPCAB** | | | | | | | | |  |
| **4** | Cohort Prospective, Cohort Retrospective ,RCT | Serious | Not serious | Not serious | Not serious | Undetected | 1.65 [0.97;   2.80] | Moderate | |
| **C-ONCAB vs MIDCAB** | | | | | | | | |  |
| **1** | Cohort Retrospective | Serious | Not serious | Not serious | Not serious | Undetected | 1.60 [0.05;  48.34] | Moderate | |
| **C-ONCAB vs OPCAB** | | | | | | | | |  |
| **13** | Cohort Prospective, Cohort Retrospective ,RCT | Serious | Not serious | Not serious | Not serious | Undetected | 1.93 [1.33;   2.81] | Moderate | |
| **Endo-CABG vs MIDCAB** | | | | | | | | |  |
| **1** | Cohort Retrospective | Serious | Not serious | Not serious | Not serious | Undetected | 0.60 [0.04;   9.17] | Moderate | |
| **Endo-CABG vs RADCAB** | | | | | | | | |  |
| **1** | Cohort Retrospective | Serious | Not serious | Not serious | Not serious | Undetected | 0.16 [0.00;   5.52] | Moderate | |
| **MIDCAB vs OPCAB** | | | | | | | | |  |
| **2** | Cohort Prospective | Serious | Not serious | Not serious | Not serious | Undetected | 1.03 [0.32;   3.27 | Moderate | |
| **MIDCAB vs PA-CABG** | | | | | | | | |  |
| **2** | Cohort Prospective, Cohort Retrospective | Serious | Not serious | Not serious | Not serious | Undetected | 0.93 [0.32;   2.73] | Moderate | |
| **MIDCAB vs RADCAB** | | | | | | | | |  |
| **1** | Cohort Retrospective | Serious | Not serious | Not serious | Not serious | Undetected | 0.27 [0.01;   7.55] | Moderate | |
| **MIDCAB vs TECAB** | | | | | | | | |  |
| **1** | Cohort Retrospective | Serious | Not serious | Not serious | Not serious | Undetected | 1.03 [0.29;   3.62] | Moderate | |
| **OPCAB vs PA-CABG** | | | | | | | | |  |
| **1** | Cohort Prospective | Serious | Not serious | Not serious | Not serious | Undetected | 0.65 [0.11;   3.84] | Moderate | |
| **OPCAB vs TECAB** | | | | | | | | |  |
| **1** | Cohort Retrospective | Serious | Not serious | Not serious | Not serious | Undetected | 5.32 [0.22; 130.11] | Moderate | |
| **PA-CABG vs TECAB** | | | | | | | | |  |
| **1** | Cohort Retrospective | Serious | Not serious | Not serious | Not serious | Undetected | 1.09 [0.30;   3.93] | Moderate | |
