# Supplementary material for: Clinical outcomes in various coronary artery bypass grafting techniques: A systematic review and network meta-analysis
Source: PLoS One. 2026 Nov 5;21(11):e0360757. doi: 10.1371/journal.pone.0360757 (PMC13739143; doi:10.1371/journal.pone.0360757)
Supplement: Supplementary file 3 [file pone.0360757.s003.zip › Supplementary Table 13.docx]

# Supp. Table 13. The evidence findings for three main techniques in cerebrovascular accident

| **Certainty assessment** | | | | | | | **Effect** | **Certainty** |
| --- | --- | --- | --- | --- | --- | --- | --- | --- |
| **№ of studies** | **Study design** | **Risk of bias** | **Inconsistency** | **Indirectness** | **Imprecision** | **Publication bias** | **SMD with 95% CI** |  |
| **Conventional vs Minimal Invasive** | | | | | | | | |
| **11** | Cohort Prospective, Cohort Retrospective | Serious | Not serious | Not serious | Not serious | Undetected | 0.84 [0.36; 1.96] | Moderate |
| **Conventional vs Robotic** | | | | | | | | |
| **12** | Cohort Prospective, Cohort Retrospective | Serious | Not serious | Not serious | Not serious | Undetected | 1.80 [1.19; 2.72] | Moderate |
| **Minimal Invasive vs Robotic** | | | | | | | | |
| **3** | Cohort Retrospective | Serious | Not serious | Not serious | Not serious | Undetected | 0.65 [0.18; 2.30] | Moderate |
| **Conventional vs Minimal Invasive (Indirect)** | | | | | | | | |
| **11** | Cohort Prospective, Cohort Retrospective | Serious | Not serious | Not serious | Not serious | Undetected | 1.19 [0.58; 2.41] | Moderate |
| **Conventional vs Robotic (Indirect)** | | | | | | | | |
| **12** | Cohort Prospective, Cohort Retrospective | Serious | Not serious | Not serious | Not serious | Undetected | 1.66 [1.12; 2.47] | Moderate |
| **Minimal Invasive vs Robotic (Indirect)** | | | | | | | | |
| **3** | Cohort Retrospective | Serious | Not serious | Not serious | Not serious | Undetected | 1.40 [0.66; 2.97] | Moderate |
