# Supplementary material for: Clinical outcomes in various coronary artery bypass grafting techniques: A systematic review and network meta-analysis
Source: PLoS One. 2026 Nov 5;21(11):e0360757. doi: 10.1371/journal.pone.0360757 (PMC13739143; doi:10.1371/journal.pone.0360757)
Supplement: Supplementary file 3 [file pone.0360757.s003.zip › Supplementary Table 14.docx]

# Supp. Table 14. The evidence findings for nine sub-techniques in cerebrovascular accident

| **Certainty assessment** | | | | | | | **Effect** | **Certainty** | |
| --- | --- | --- | --- | --- | --- | --- | --- | --- | --- |
| **№ of studies** | **Study design** | **Risk of bias** | **Inconsistency** | **Indirectness** | **Imprecision** | **Publication bias** | **SMD with 95% CI** |  |  |
| **BH-ONCAB vs C-ONCAB** | | | | | | | | |  |
| **13** | Cohort Prospective, Cohort Retrospective ,RCT | Serious | Not serious | Not serious | Not serious | Undetected | 1.18 [0.89;   1.58] | Moderate | |
| **BH-ONCAB vs OPCAB** | | | | | | | | |  |
| **3** | RCT | Not serious | Not serious | Not serious | Not serious | Undetected | 1.73 [1.29;   2.31] | High | |
| **C-ONCAB vs OPCAB** | | | | | | | | |  |
| **48** | Cohort Prospective, Cohort Retrospective ,RCT | Serious | Not serious | Not serious | Not serious | Undetected | 1.45 [1.34;   1.56] | Moderate | |
| **C-ONCAB vs PA-CABG** | | | | | | | | |  |
| **1** | Cohort Retrospective | Serious | Not serious | Not serious | Not serious | Undetected | 0.49 [0.13;   1.90] | Moderate | |
| **C-ONCAB vs RADCAB** | | | | | | | | |  |
| **1** | Cohort Prospective | Serious | Not serious | Not serious | Not serious | Undetected | 1.64 [0.39;   6.87] | Moderate | |
| **C-ONCAB vs TECAB** | | | | | | | | |  |
| **1** | Cohort Retrospective | Serious | Not serious | Not serious | Not serious | Undetected | 3.55 [0.14;  90.24] | Moderate | |
| **MICS vs OPCAB** | | | | | | | | |  |
| **3** | Cohort Prospective, Cohort Retrospective | Serious | Not serious | Not serious | Not serious | Undetected | 0.56 [0.12;   2.55] | Moderate | |
| **MIDCAB vs OPCAB** | | | | | | | | |  |
| **5** | Cohort Prospective, Cohort Retrospective | Serious | Not serious | Not serious | Not serious | Undetected | 1.23 [0.30;   5.12] | Moderate | |
| **MIDCAB vs PA-CABG** | | | | | | | | |  |
| **3** | Cohort Prospective, Cohort Retrospective | Serious | Not serious | Not serious | Not serious | Undetected | 0.76 [0.11;   5.10] | Moderate | |
| **MIDCAB vs RADCAB** | | | | | | | | |  |
| **2** | Cohort Retrospective | Serious | Not serious | Not serious | Not serious | Undetected | 0.55 [0.14;   2.16] | Moderate | |
| **MIDCAB vs TECAB** | | | | | | | | |  |
| **1** | Cohort Retrospective | Serious | Not serious | Not serious | Not serious | Undetected | 1.11 [0.02;  57.16] | Moderate | |
| **OPCAB vs PA-CABG** | | | | | | | | |  |
| **1** | Cohort Prospective | Serious | Not serious | Not serious | Not serious | Undetected | 0.50 [0.02;  15.33] | Moderate | |
| **OPCAB vs RADCAB** | | | | | | | | |  |
| **3** | Cohort Prospective | Serious | Not serious | Not serious | Not serious | Undetected | 1.64 [0.34;   7.77 | Moderate | |
| **PA-CABG vs RADCAB** | | | | | | | | |  |
| **1** | Cohort Retrospective | Serious | Not serious | Not serious | Not serious | Undetected | 3.12 [0.06; 158.55] | Moderate | |
| **PA-CABG vs TECAB** | | | | | | | | |  |
| **1** | Cohort Retrospective | Serious | Not serious | Not serious | Not serious | Undetected | 2.49 [0.08;  75.82] | Moderate | |
