# Supplementary material for: Clinical outcomes in various coronary artery bypass grafting techniques: A systematic review and network meta-analysis
Source: PLoS One. 2026 Nov 5;21(11):e0360757. doi: 10.1371/journal.pone.0360757 (PMC13739143; doi:10.1371/journal.pone.0360757)
Supplement: Supplementary file 3 [file pone.0360757.s003.zip › Supplementary Table 16.docx]

# Supp. Table 16. The evidence findings for nine sub-techniques in myocardial infarction

| **Certainty assessment** | | | | | | | **Effect** | **Certainty** |
| --- | --- | --- | --- | --- | --- | --- | --- | --- |
| **№ of studies** | **Study design** | **Risk of bias** | **Inconsistency** | **Indirectness** | **Imprecision** | **Publication bias** | **SMD with 95% CI** |  |

| **BH-ONCAB vs C-ONCAB** | | | | | | | | | | | | | | |  |  |  |  |  |  |  |  |
| --- | --- | --- | --- | --- | --- | --- | --- | --- | --- | --- | --- | --- | --- | --- | --- | --- | --- | --- | --- | --- | --- | --- |
| 8 | Cohort Prospective, Cohort Retrospective, RCT | | Serious | Not serious | | Not serious | Not serious | | Undetected | | | 0.63 [0.37; 1.08] | Moderate | | Cohort Retrospective | Serious | Not serious | Not serious | Not serious | Undetected | 1.51 [0.25;    9.25] | Moderate |
| **C-ONCAB vs OPCAB** | | | | | | | | | | | | | | |  |  |  |  |  |  |  |  |
| **31** | Cohort Prospective, Cohort Retrospective, RCT | | Serious | Not serious | | Not serious | Not serious | | Undetected | | | **1.27 [1.09; 1.48]** | Moderate | |  |  |  |  |  |  |  |  |
| **MICS vs OPCAB** | | | | | | | | | | | | | | |  |  |  |  |  |  |  |  |
| **1** | | Cohort Retrospective | Serious | Not serious | Not serious | | | Not serious | | Undetected | 1.51 [0.25;    9.25] | | Moderate |  |  |  |  |  |  |  |  |  |
| **MIDCAB vs OPCAB** | | | | | | | | | | | | | | |  |  |  |  |  |  |  |  |
| **8** | | Cohort Prospective | Serious | Not serious | Not serious | | | Not serious | | Undetected | 1.60 [0.71;    3.60] | | Moderate |  |  |  |  |  |  |  |  |  |
| **MIDCAB vs PA-CABG** | | | | | | | | | | | | | | |  |  |  |  |  |  |  |  |
| **2** | | Cohort Prospective, Cohort Retrospective | Serious | Not serious | Not serious | | | Not serious | | Undetected | 0.09 [0.01;    0.99] | | Moderate |  |  |  |  |  |  |  |  |  |
| **MIDCAB vs RADCAB** | | | | | | | | | | | | | | |  |  |  |  |  |  |  |  |
| **3** | | Cohort Retrospective | Serious | Not serious | Not serious | | | Not serious | | Undetected | 1.07 [0.27;    4.27] | | Moderate |  |  |  |  |  |  |  |  |  |
| **MIDCAB vs TECAB** | | | | | | | | | | | | | | |  |  |  |  |  |  |  |  |
| **1** | | Cohort Retrospective | Serious | Not serious | Not serious | | | Not serious | | Undetected | 0.21 [0.01;    4.61] | | Moderate |  |  |  |  |  |  |  |  |  |
| **OPCAB vs PA-CABG** | | | | | | | | | | | | | | |  |  |  |  |  |  |  |  |
| **1** | | Cohort Prospective | Serious | Not serious | Not serious | | | Not serious | | Undetected | 0.01 [0.00;    0.44] | | Moderate |  |  |  |  |  |  |  |  |  |
| **OPCAB vs RADCAB** | | | | | | | | | | | | | | |  |  |  |  |  |  |  |  |
| **2** | | Cohort Prospective | Serious | Not serious | Not serious | | | Not serious | | Undetected | 1.61 [0.08;   31.79] | | Moderate |  |  |  |  |  |  |  |  |  |
| **OPCAB vs TECAB** | | | | | | | | | | | | | | |  |  |  |  |  |  |  |  |
| **1** | | Cohort Retrospective | Serious | Not serious | Not serious | | | Not serious | | Undetected | 0.96 [0.06;   15.67] | | Moderate |  |  |  |  |  |  |  |  |  |
| **PA-CABG vs TECAB** | | | | | | | | | | | | | | |  |  |  |  |  |  |  |  |
| **1** | | Cohort Retrospective | Serious | Not serious | Not serious | | | Not serious | | Undetected | 0.73 [0.09;    5.76] | | Moderate |  |  |  |  |  |  |  |  |  |
| **RADCAB vs TECAB** | | | | | | | | | | | | | | |  |  |  |  |  |  |  |  |
| **1** | | Cohort Retrospective | Serious | Not serious | Not serious | | | Not serious | | Undetected | 0.67 [0.03;   14.23] | | Moderate |  |  |  |  |  |  |  |  |  |
