# Supplementary material for: Clinical outcomes in various coronary artery bypass grafting techniques: A systematic review and network meta-analysis
Source: PLoS One. 2026 Nov 5;21(11):e0360757. doi: 10.1371/journal.pone.0360757 (PMC13739143; doi:10.1371/journal.pone.0360757)
Supplement: Supplementary file 3 [file pone.0360757.s003.zip › Supplementary Table 18.docx]

# Supp. Table 18. The evidence findings for nine sub-techniques in non sinus rhytm

| **Certainty assessment** | | | | | | | **Effect** | **Certainty** | |
| --- | --- | --- | --- | --- | --- | --- | --- | --- | --- |
| **№ of studies** | **Study design** | **Risk of bias** | **Inconsistency** | **Indirectness** | **Imprecision** | **Publication bias** | **SMD with 95% CI** |  |  |
| **BH-ONCAB vs C-ONCAB** | | | | | | | | |  |
| **11** | Cohort Prospective, Cohort Retrospective ,RCT | Serious | Not serious | Not serious | Not serious | Undetected | 0.87 [0.65;  1.15] | Moderate | |
| **BH-ONCAB vs OPCAB** | | | | | | | | |  |
| **13** | Cohort Prospective, Cohort Retrospective ,RCT | Serious | Not serious | Not serious | Not serious | Undetected | 1.54 [1.20;  1.98] | Moderate | |
| **C-ONCAB vs MIDCAB** | | | | | | | | |  |
| **2** | Cohort Retrospective | Serious | Serious | Not serious | Not serious | Undetected | 1.29 [0.65;  2.56] | Low | |
| **C-ONCAB vs OPCAB** | | | | | | | | |  |
| **32** | Cohort Prospective, Cohort Retrospective ,RCT | Serious | Not serious | Not serious | Not serious | Undetected | 1.29 [1.15;  1.46] | Moderate | |
| **C-ONCAB vs PA-CABG** | | | | | | | | |  |
| **2** | Cohort Retrospective | Serious | Not serious | Not serious | Not serious | Undetected | 1.38 [0.87;  2.18] | Moderate | |
| **C-ONCAB vs RADCAB** | | | | | | | | |  |
| **2** | Cohort Retrospective | Serious | Serious | Not serious | Not serious | Undetected | 1.45 [0.81;  2.59] | Low | |
| **C-ONCAB vs TECAB** | | | | | | | | |  |
| **1** | Cohort Prospective | Serious | Serious | Not serious | Not serious | Undetected | 0.44 [0.10;  1.98] | Low | |
| **Endo-CABG vs MIDCAB** | | | | | | | | |  |
| **1** | Cohort Prospective, Cohort Retrospective | Serious | Not serious | Not serious | Not serious | Undetected | 1.03 [0.46;  2.29] | Moderate | |

| **MICS vs OPCAB** | | | | | | | | |  |
| --- | --- | --- | --- | --- | --- | --- | --- | --- | --- |
| **3** | Cohort Prospective, Cohort Retrospective | Serious | Serious | Not serious | Not serious | Undetected | 0.87 [0.60;  1.28] | Low | |
| **MIDCAB vs OPCAB** | | | | | | | | |  |
| **8** | Cohort Prospective, Cohort Retrospective | Serious | Serious | Not serious | Not serious | Undetected | 0.93 [0.64;  1.35] | Low | |
| **MIDCAB vs RADCAB** | | | | | | | | |  |
| **3** | Cohort Retrospective | Serious | Not serious | Not serious | Not serious | Undetected | 1.17 [0.79;  1.73] | Moderate | |
| **OPCAB vs RADCAB** | | | | | | | | |  |
| **3** | Cohort Prospective, Cohort Retrospective | Serious | Serious | Not serious | Not serious | Undetected | 1.73 [1.12;  2.68] | Low | |
| **OPCAB vs TECAB** | | | | | | | | |  |
| **1** | Cohort Retrospective | Serious | Serious | Not serious | Not serious | Undetected | 0.96 [0.06; 16.02] | Low | |
