# Supplementary material for: Clinical outcomes in various coronary artery bypass grafting techniques: A systematic review and network meta-analysis
Source: PLoS One. 2026 Nov 5;21(11):e0360757. doi: 10.1371/journal.pone.0360757 (PMC13739143; doi:10.1371/journal.pone.0360757)
Supplement: Supplementary file 3 [file pone.0360757.s003.zip › Supplementary Table 2.docx]

# **Supplementary table 2A-N.** Ranking netleague of nine sub-techniques

| **A. Operative time** | | | | | | | | |
| --- | --- | --- | --- | --- | --- | --- | --- | --- |
| BH-ONCAB | -2.66 [ -49.00; 43.69] | - | - | - | 31.35 [ -12.47; 75.18] | - | - | - |
| -6.74 [ -43.13; 29.64] | C-ONCAB | - | - | 51.20 [ -66.84; 169.24] | 42.17 [ 13.29; 71.06] | - | - | - |
| 72.88 [ -38.05; 183.80] | 79.62 [ -28.40; 187.63] | Endo-CABG | - | 17.80 [ -96.56; 132.16] | - | - | -111.00 [-225.36; 3.36] | - |
| -18.07 [ -93.94; 57.79] | -11.33 [ -83.25; 60.59] | -90.95 [-215.62; 33.73] | MICS | - | 51.60 [ -15.29; 118.49] | - | - | - |
| 52.67 [ 2.49; 102.85] | 59.41 [ 16.22; 102.61] | -20.20 [-121.97; 81.56] | 70.75 [ -5.23; 146.72] | MIDCAB | -29.44 [ -71.28; 12.39] | -57.47 [-139.56; 24.63] | -43.26 [-109.26; 22.73] | -18.00 [-134.69; 98.69] |
| 33.53 [ -2.27; 69.32] | 40.27 [ 13.86; 66.68] | -39.35 [-144.56; 65.86] | 51.60 [ -15.29; 118.49] | -19.15 [ -55.17; 16.88] | OPCAB | -90.00 [-205.79; 25.79] | -48.29 [-115.02; 18.44] | - |
| -15.78 [ -99.66; 68.09] | -9.04 [ -89.08; 71.00] | -88.66 [-211.83; 34.51] | 2.29 [ -99.04; 103.62] | -68.46 [-141.93; 5.01] | -49.31 [-125.43; 26.80] | PA-CABG | - | -12.00 [-127.23; 103.23] |
| -0.12 [ -59.78; 59.53] | 6.62 [ -47.57; 60.81] | -73.00 [-174.76; 28.76] | 17.95 [ -64.42; 100.32] | -52.79 [ -99.49; -6.10] | -33.65 [ -81.72; 14.42] | 15.66 [ -64.41; 95.74] | RADCAB | -11.88 [ -69.40; 45.64] |
| -8.37 [ -82.32; 65.59] | -1.62 [ -71.22; 67.97] | -81.24 [-193.32; 30.84] | 9.71 [ -83.57; 102.98] | -61.04 [-123.65; 1.58] | -41.89 [-106.90; 23.11] | 7.42 [ -76.67; 91.51] | -8.24 [ -59.94; 43.46] | TECAB |
| **B. ICU stay** | | | | | | | | |
| BH-ONCAB | -0.15 [ -0.52; 0.22] | - | - | 0.32 [ -0.10; 0.74] | - | - | - | - |
| -0.23 [-0.55; 0.08] | C-ONCAB | - | 0.49 [ -0.45; 1.42] | 0.77 [ 0.51; 1.03] | 0.58 [ -0.58; 1.74] | 0.60 [ -0.61; 1.81] | 0.58 [ -0.28; 1.44] | - |
| 1.14 [ 0.46; 1.83] | 1.38 [ 0.73; 2.02] | MICS | -40.20 [-45.01; -35.39] | -0.47 [ -1.07; 0.13] | - | - | - | - |
| 0.63 [ 0.12; 1.15] | 0.87 [ 0.42; 1.31] | -0.51 [-1.25; 0.23] | MIDCAB | -0.06 [ -0.65; 0.52] | -0.24 [ -0.99; 0.52] | 0.18 [ -0.49; 0.85] | 0.10 [ -0.74; 0.95] | - |
| 0.36 [ 0.03; 0.68] | 0.59 [ 0.36; 0.82] | -0.79 [-1.39; -0.18] | -0.28 [-0.71; 0.16] | OPCAB | 0.00 [ -1.47; 1.47] | 4.13 [ 2.89; 5.37] | . | - |
| 0.44 [-0.25; 1.14] | 0.68 [ 0.04; 1.32] | -0.70 [-1.58; 0.18] | -0.19 [-0.81; 0.43] | 0.09 [-0.56; 0.73] | PA-CABG | 0.30 [ -0.87; 1.47] | 0.70 [ -0.70; 2.10] | - |
| 1.22 [ 0.62; 1.81] | 1.45 [ 0.92; 1.98] | 0.07 [-0.73; 0.88] | 0.58 [ 0.08; 1.08] | 0.86 [ 0.32; 1.39] | 0.77 [ 0.09; 1.46] | RADCAB | -0.07 [ -0.86; 0.72] | - |
| 0.83 [ 0.18; 1.47] | 1.06 [ 0.48; 1.64] | -0.32 [-1.16; 0.53] | 0.19 [-0.38; 0.76] | 0.47 [-0.12; 1.06] | 0.38 [-0.35; 1.12] | -0.39 [-0.96; 0.18] | TECAB | - |
| **C. Length of stay** | | | | | | | | |
| BH-ONCAB | -0.53 [ -1.28; 0.22] | - | - | - | 0.93 [ 0.25; 1.61] | - | - | - |
| -0.37 [-0.94; 0.20] | C-ONCAB | - | - | 1.98 [ 0.65; 3.31] | 1.14 [ 0.76; 1.52] | 8.60 [ 6.77; 10.43] | 1.64 [ 0.32; 2.95] | 5.01 [ 3.46; 6.57] |
| 2.82 [ 1.12; 4.52] | 3.19 [ 1.56; 4.82] | Endo-CABG | - | 0.00 [ -1.78; 1.78] | - | - | -0.40 [ -2.18; 1.38] | - |
| 2.30 [ 0.94; 3.67] | 2.67 [ 1.38; 3.97] | -0.51 [-2.56; 1.53] | MICS | - | -1.54 [ -2.79; -0.30] | - | - | - |
| 2.40 [ 1.64; 3.16] | 2.77 [ 2.17; 3.36] | -0.42 [-1.99; 1.15] | 0.09 [-1.27; 1.46] | MIDCAB | -2.04 [ -2.76; -1.32] | -0.70 [ -2.26; 0.86] | 1.05 [ 0.16; 1.93] | 2.99 [ 1.46; 4.51] |
| 0.76 [ 0.20; 1.32] | 1.13 [ 0.78; 1.47] | -2.06 [-3.68; -0.44] | -1.54 [-2.79; -0.30] | -1.64 [-2.19; -1.09] | OPCAB | 0.00 [ -2.52; 2.52] | 1.57 [ 0.53; 2.61] | - |
| 4.18 [ 2.92; 5.44] | 4.55 [ 3.40; 5.70] | 1.37 [-0.56; 3.29] | 1.88 [ 0.18; 3.58] | 1.78 [ 0.62; 2.95] | 3.42 [ 2.27; 4.58] | PA-CABG | - | 1.50 [ -0.51; 3.51] |
| 2.84 [ 2.01; 3.67] | 3.21 [ 2.54; 3.87] | 0.02 [-1.55; 1.59] | 0.54 [-0.87; 1.94] | 0.44 [-0.21; 1.09] | 2.08 [ 1.44; 2.72] | -1.34 [-2.59; -0.10] | RADCAB | -0.50 [ -2.28; 1.28] |
| **D. Mortality** | | | | | | | | |
| BH-ONCAB | 0.84 [0.51; 1.38] | - | - | - | 1.25 [0.75; 2.06] | - | - | - |
| 0.85 [0.57; 1.26] | C-ONCAB | - | - | 0.92 [0.09; 9.65] | 1.34 [1.00; 1.79] | 1.50 [0.18; 12.39] | - | - |
| 2.57 [0.08; 85.18] | 3.03 [0.09; 98.86] | Endo-CABG | - |  | - | - | 1.03 [0.03; 41.42] | - |
| 2.16 [0.24; 19.20] | 2.55 [0.29; 22.17] | 0.84 [0.01; 50.20] | MICS | - | 0.52 [0.06; 4.47] | - | - | - |
| 1.63 [0.60; 4.43] | 1.92 [0.74; 4.96] | 0.63 [0.02; 18.95] | 0.75 [0.07; 7.81] | MIDCAB | 0.67 [0.21; 2.13] | 0.51 [0.03; 8.61] | 1.09 [0.24; 4.93] | 0.37 [0.01; 14.88] |
| 1.13 [0.76; 1.68] | 1.33 [1.01; 1.74] | 0.44 [0.01; 14.24] | 0.52 [0.06; 4.47] | 0.69 [0.27; 1.75] | OPCAB | 0.33 [0.01; 13.58] | 1.81 [0.27; 11.96] | 4.90 [0.14; 171.96] |
| 1.12 [0.22; 5.76] | 1.31 [0.26; 6.51] | 0.43 [0.01; 19.12] | 0.52 [0.04; 7.54] | 0.68 [0.12; 3.87] | 0.99 [0.20; 4.93] | PA-CABG | . | 0.40 [0.01; 16.47] |
| 1.88 [0.48; 7.34] | 2.21 [0.59; 8.34] | 0.73 [0.02; 22.51] | 0.87 [0.07; 10.73] | 1.15 [0.34; 3.95] | 1.67 [0.45; 6.15] | 1.68 [0.23; 12.52] | RADCAB | . |
| 1.39 [0.13; 15.24] | 1.63 [0.15; 17.55] | 0.54 [0.01; 34.09] | 0.64 [0.03; 15.66] | 0.85 [0.08; 9.52] | 1.23 [0.12; 13.11] | 1.24 [0.10; 16.07] | 0.74 [0.05; 10.26] | TECAB |
| **E. Complete revascularization** | | | | | | | | |
| BH-ONCAB | 0.50 [0.24; 1.03] | - | - | 1.65 [0.97; 2.80] | - | - | - |  |
| 0.67 [0.40; 1.11] | C-ONCAB | - | 1.60 [0.05; 48.34] | 1.93 [1.33; 2.81] | - | - | - |  |
| 2.44 [0.13; 45.93] | 3.65 [0.20; 67.51] | Endo-CABG | 0.60 [0.04; 9.17] | - | - | 0.16 [0.00; 5.52] | - |  |
| 1.47 [0.49; 4.46] | 2.21 [0.77; 6.36] | 0.60 [0.04; 9.17] | MIDCAB | 1.03 [0.32; 3.27] | 0.93 [0.32; 2.73] | 0.27 [0.01; 7.55] | 1.03 [0.29; 3.62] |  |
| 1.40 [0.88; 2.22] | 2.09 [1.47; 2.97] | 0.57 [0.03; 10.42] | 0.95 [0.35; 2.60] | OPCAB | 0.65 [0.11; 3.84] | - | 5.32 [0.22; 130.11] |  |
| 1.34 [0.36; 5.05] | 2.01 [0.56; 7.27] | 0.55 [0.03; 10.11] | 0.91 [0.32; 2.57] | 0.96 [0.28; 3.34] | PA-CABG | - | 1.09 [0.30; 3.93] |  |
| 0.39 [0.01; 13.30] | 0.59 [0.02; 19.61] | 0.16 [0.00; 5.52] | 0.27 [0.01; 7.55] | 0.28 [0.01; 9.25] | 0.29 [0.01; 9.68] | RADCAB | - |  |
| 1.76 [0.42; 7.47] | 2.64 [0.64; 10.79] | 0.72 [0.04; 13.81] | 1.20 [0.38; 3.76] | 1.26 [0.32; 4.97] | 1.31 [0.40; 4.27] | 4.50 [0.13; 154.60] | TECAB |  |
| BH-ONCAB | 0.50 [0.24; 1.03] | - | - | 1.65 [0.97; 2.80] | - | - | - |  |
| **F. CVA** | | | | | | | | |
| BH-ONCAB | 1.18 [0.89; 1.58] | - | - | 1.73 [1.29; 2.31] | - | - | - |  |
| 1.17 [0.92; 1.49] | C-ONCAB | - | - | 1.45 [1.34; 1.56] | 0.49 [0.13; 1.90] | 1.64 [0.39; 6.87] | 3.55 [0.14; 90.24] |  |
| 3.03 [0.65; 14.13] | 2.59 [0.56; 11.85] | MICS | - | 0.56 [0.12; 2.55] | - | - | - |  |
| 1.90 [0.67; 5.39] | 1.62 [0.59; 4.48] | 0.63 [0.10; 3.89] | MIDCAB | 1.23 [0.30; 5.12] | 0.76 [0.11; 5.10] | 0.55 [0.14; 2.16] | 1.11 [0.02; 57.16] |  |
| 1.70 [1.33; 2.15] | 1.45 [1.34; 1.56] | 0.56 [0.12; 2.55] | 0.89 [0.32; 2.47] | OPCAB | 0.50 [0.02; 15.33] | 1.64 [0.34; 7.77] | . |  |
| 0.78 [0.25; 2.37] | 0.66 [0.22; 1.97] | 0.26 [0.04; 1.66] | 0.41 [0.11; 1.50] | 0.46 [0.15; 1.36] | PA-CABG | 3.12 [0.06; 158.55] | 2.49 [0.08; 75.82] |  |
| 1.67 [0.65; 4.30] | 1.43 [0.57; 3.56] | 0.55 [0.09; 3.25] | 0.88 [0.31; 2.54] | 0.99 [0.40; 2.46] | 2.15 [0.56; 8.21] | RADCAB | - |  |
| 2.88 [0.28; 29.71] | 2.45 [0.24; 25.04] | 0.95 [0.06; 15.23] | 1.52 [0.13; 17.20] | 1.70 [0.17; 17.33] | 3.70 [0.34; 40.29] | 1.72 [0.15; 20.03] | TECAB |  |
| **G. MI** | | | | | | | | |
| BH-ONCAB | 0.63 [0.37; 1.08] | - | - | 1.16 [0.67; 2.02] | - | - | - |  |
| 0.73 [0.49; 1.10] | C-ONCAB | - | - | 1.27 [1.09; 1.48] | - | - | - |  |
| 0.62 [0.10; 3.99] | 0.85 [0.14; 5.24] | MICS | - | 1.51 [0.25; 9.25] | - | - | - |  |
| 0.66 [0.27; 1.57] | 0.90 [0.41; 1.97] | 1.06 [0.15; 7.57] | MIDCAB | 1.60 [0.71; 3.60] | 0.09 [0.01; 0.99] | 1.07 [0.27; 4.27] | 0.21 [0.01; 4.61] |  |
| 0.94 [0.62; 1.42] | 1.28 [1.10; 1.49] | 1.51 [0.25; 9.25] | 1.43 [0.66; 3.08] | OPCAB | 0.01 [0.00; 0.44] | 1.61 [0.08; 31.79] | 0.96 [0.06; 15.67] |  |
| 0.14 [0.02; 1.19] | 0.19 [0.02; 1.57] | 0.23 [0.01; 3.61] | 0.21 [0.03; 1.67] | 0.15 [0.02; 1.22] | PA-CABG | - | 0.73 [0.09; 5.76] |  |
| 0.72 [0.18; 2.91] | 0.99 [0.26; 3.77] | 1.16 [0.12; 11.00] | 1.10 [0.33; 3.64] | 0.77 [0.20; 2.91] | 5.13 [0.54; 48.69] | RADCAB | 0.67 [0.03; 14.23] |  |
| 0.25 [0.04; 1.47] | 0.35 [0.06; 1.93] | 0.41 [0.03; 4.94] | 0.39 [0.07; 2.09] | 0.27 [0.05; 1.50] | 1.81 [0.28; 11.54] | 0.35 [0.06; 2.22] | TECAB |  |
| **H. Non-SR** |  |  |  |  |  |  |  |  |
|  | | | | | | | | |
| BH-ONCAB | 0.87 [0.65; 1.15] | - | - | - | 1.54 [1.20; 1.98] | - | - | - |
| 1.06 [0.86; 1.32] | C-ONCAB | - | - | 1.29 [0.65; 2.56] | 1.29 [1.15; 1.46] | 1.38 [0.87; 2.18] | 1.45 [0.81; 2.59] | 0.44 [0.10; 1.98] |
| 1.46 [0.66; 3.22] | 1.37 [0.64; 2.96] | Endo-CABG | - | 1.03 [0.46; 2.29] | - | - | 1.34 [0.61; 2.97] | - |
| 1.58 [1.02; 2.43] | 1.48 [1.00; 2.20] | 1.08 [0.46; 2.54] | MICS | - | 0.87 [0.60; 1.28] | - | - | - |
| 1.53 [1.08; 2.16] | 1.44 [1.07; 1.92] | 1.05 [0.50; 2.20] | 0.97 [0.61; 1.55] | MIDCAB | 0.93 [0.64; 1.35] | - | 1.17 [0.79; 1.73] | - |
| 1.38 [1.12; 1.70] | 1.29 [1.16; 1.45] | 0.94 [0.44; 2.03] | 0.87 [0.60; 1.28] | 0.90 [0.68; 1.19] | OPCAB | - | 1.73 [1.12; 2.68] | 0.96 [0.06; 16.02] |
| 1.46 [0.88; 2.43] | 1.38 [0.87; 2.18] | 1.00 [0.41; 2.46] | 0.93 [0.51; 1.71] | 0.96 [0.56; 1.65] | 1.06 [0.66; 1.71] | PA-CABG | - | - |
| 1.92 [1.35; 2.73] | 1.81 [1.34; 2.43] | 1.32 [0.63; 2.76] | 1.22 [0.76; 1.96] | 1.26 [0.93; 1.70] | 1.39 [1.04; 1.87] | 1.31 [0.76; 2.27] | RADCAB | - |
| 0.59 [0.16; 2.26] | 0.56 [0.15; 2.09] | 0.41 [0.09; 1.87] | 0.38 [0.09; 1.49] | 0.39 [0.10; 1.50] | 0.43 [0.11; 1.62] | 0.40 [0.10; 1.64] | 0.31 [0.08; 1.19] | TECAB |
| **I. Reoperation** | | | | | | | | |
| BH-ONCAB | 0.90 [0.55; 1.47] | - | - | - | 1.76 [1.14; 2.72] | - | - | - |
| 1.18 [0.81; 1.71] | C-ONCAB | - | - | 0.65 [0.14; 2.98] | 1.16 [0.96; 1.40] | 0.09 [0.00; 1.73] | 2.32 [0.63; 8.54] | 0.22 [0.01; 5.01] |
| 1.51 [0.15; 14.92] | 1.28 [0.13; 12.31] | Endo-CABG | - | 0.83 [0.08; 8.79] | - | - | 0.77 [0.08; 7.67] | - |
| 1.84 [0.57; 5.95] | 1.56 [0.50; 4.82] | 1.22 [0.10; 15.22] | MICS | - | 0.77 [0.25; 2.34] | - | - | - |
| 0.86 [0.43; 1.75] | 0.73 [0.39; 1.36] | 0.57 [0.06; 5.13] | 0.47 [0.13; 1.68] | MIDCAB | 2.01 [0.88; 4.58] | 2.77 [0.10; 74.03] | 1.68 [1.08; 2.61] | 0.35 [0.05; 2.57] |
| 1.41 [0.98; 2.04] | 1.20 [1.00; 1.44] | 0.94 [0.10; 9.02] | 0.77 [0.25; 2.34] | 1.64 [0.88; 3.03] | OPCAB | - | 6.43 [0.35; 117.36] | 0.32 [0.01; 8.36] |
| 0.53 [0.06; 4.74] | 0.45 [0.05; 3.89] | 0.35 [0.02; 7.71] | 0.29 [0.03; 3.29] | 0.62 [0.07; 5.46] | 0.38 [0.04; 3.27] | PA-CABG | - | 0.13 [0.01; 2.59] |
| 1.56 [0.73; 3.33] | 1.32 [0.67; 2.60] | 1.03 [0.12; 9.18] | 0.85 [0.23; 3.12] | 1.80 [1.19; 2.73] | 1.10 [0.56; 2.17] | 2.93 [0.32; 26.52] | RADCAB | 1.00 [0.06; 17.63] |
| 0.35 [0.09; 1.42] | 0.30 [0.08; 1.15] | 0.23 [0.02; 2.99] | 0.19 [0.03; 1.10] | 0.41 [0.11; 1.53] | 0.25 [0.07; 0.96] | 0.67 [0.07; 6.37] | 0.23 [0.06; 0.87] | TECAB |
| **J. Ventilation time** | | | | | | | | |
| BH-ONCAB | 1.42 [ -0.53; 3.36] | - | - | 3.51 [ 0.63; 6.39] | - | - | - | - |
| 0.66 [ -1.11; 2.44] | C-ONCAB | - | 7.93 [ 2.84; 13.03] | 6.00 [ 4.35; 7.65] | - | 0.50 [ -3.88; 4.88] | -1.90 [-10.36; 6.56] | - |
| 7.59 [ 4.24; 10.94] | - | MICS | - | -2.36 [ -5.05; 0.33] | - | - | - | - |
| 8.22 [ 5.70; 10.73] | 7.55 [ 5.52; 9.59] | 0.63 [ -2.54; 3.80] | MIDCAB | -3.52 [ -5.54; -1.50] | 2.60 [ -2.05; 7.25] | 1.38 [ -1.71; 4.46] | -3.49 [ -6.89; -0.09] | - |
| 5.23 [ 3.22; 7.23] | 4.57 [ 3.13; 6.00] | -2.36 [ -5.05; 0.33] | -2.99 [ -4.67; -1.31] | OPCAB | - | 1.71 [ -2.69; 6.10] | 5.00 [ 0.48; 9.52] | - |
| 10.13 [ 5.47; 14.80] | 9.47 [ 5.05; 13.89] | 2.55 [ -2.51; 7.60] | 1.92 [ -2.18; 6.01] | 4.91 [ 0.63; 9.19] | PA-CABG | - | -3.40 [ -7.92; 1.12] | - |
| 7.05 [ 4.30; 9.80] | 6.39 [ 4.10; 8.68] | -0.54 [ -3.94; 2.87] | -1.17 [ -3.18; 0.85] | 1.82 [ -0.27; 3.91] | -3.08 [ -7.33; 1.17] | RADCAB | -1.15 [ -3.37; 1.07] | - |
| 6.12 [ 3.22; 9.03] | 5.46 [ 2.97; 7.95] | -1.46 [ -4.98; 2.05] | -2.09 [ -4.26; 0.07] | 0.90 [ -1.36; 3.15] | -4.01 [ -8.07; 0.05] | -0.93 [ -2.80; 0.94] | TECAB | - |
| **K. Wound infection** | | | | | | | | |
| BH-ONCAB | 0.89 [0.62; 1.28] | . | . | . | 1.32 [0.98; 1.76] | . | . | . |
| 0.99 [0.76; 1.30] | C-ONCAB | . | . | 2.28 [0.40; 12.99] | 1.25 [1.13; 1.39] | 2.81 [1.21; 6.55] | 10.35 [1.95; 54.80] | 8.84 [0.44; 177.66] |
| 4.82 [0.98; 23.79] | 4.85 [1.00; 23.44] | Endo-CABG | . | 0.29 [0.07; 1.30] | . | . | 1.57 [0.28; 8.73] | . |
| 5.97 [1.24; 28.74] | 6.00 [1.27; 28.35] | 1.24 [0.14; 11.27] | MICS | . | 0.21 [0.04; 1.00] | . | . | . |
| 1.50 [0.81; 2.78] | 1.51 [0.86; 2.65] | 0.31 [0.07; 1.37] | 0.25 [0.05; 1.30] | MIDCAB | 1.10 [0.57; 2.12] | 0.33 [0.03; 3.30] | 3.39 [1.34; 8.60] | . |
| 1.26 [0.97; 1.65] | 1.27 [1.15; 1.41] | 0.26 [0.05; 1.26] | 0.21 [0.04; 1.00] | 0.84 [0.48; 1.48] | OPCAB | . | 9.77 [0.51; 186.52] | 0.32 [0.01; 7.87] |
| 2.28 [0.98; 5.28] | 2.29 [1.03; 5.08] | 0.47 [0.08; 2.71] | 0.38 [0.07; 2.18] | 1.52 [0.60; 3.88] | 1.80 [0.81; 4.02] | PA-CABG | . | . |
| 6.03 [2.46; 14.77] | 6.06 [2.57; 14.31] | 1.25 [0.25; 6.20] | 1.01 [0.17; 5.94] | 4.02 [1.83; 8.85] | 4.77 [2.02; 11.25] | 2.65 [0.84; 8.34] | RADCAB | 1.12 [0.05; 27.88] |
| 3.03 [0.48; 19.27] | 3.04 [0.49; 19.03] | 0.63 [0.06; 6.73] | 0.51 [0.05; 5.59] | 2.02 [0.31; 13.25] | 2.40 [0.38; 14.98] | 1.33 [0.18; 9.75] | 0.50 [0.07; 3.37] | TECAB |
| **L. Renal failure** | | | | | | | | |
| BH-ONCAB | 0.95 [0.61; 1.48] | - | - | - | 2.37 [1.38; 4.07] | - | - |  |
| 1.13 [0.77; 1.66] | C-ONCAB | - | - | 3.63 [0.51; 25.92] | 1.39 [1.09; 1.76] | 4.05 [0.63; 26.16] | - |  |
| 2.71 [0.44; 16.63] | 2.39 [0.40; 14.13] | Endo-CABG | - | 1.53 [0.32; 7.32] | - | 2.16 [0.45; 10.44] | - |  |
| 1.94 [0.46; 8.21] | 1.71 [0.42; 6.97] | 0.72 [0.07; 6.84] | MICS | - | 0.84 [0.21; 3.37] | - | - |  |
| 4.77 [1.43; 15.87] | 4.20 [1.34; 13.22] | 1.76 [0.42; 7.41] | 2.46 [0.41; 14.91] | MIDCAB | 0.27 [0.04; 1.83] | 1.09 [0.46; 2.59] | - |  |
| 1.63 [1.10; 2.43] | 1.44 [1.14; 1.81] | 0.60 [0.10; 3.58] | 0.84 [0.21; 3.37] | 0.34 [0.11; 1.08] | OPCAB | - | - |  |
| 5.09 [1.42; 18.32] | 4.49 [1.32; 15.31] | 1.88 [0.44; 7.95] | 2.62 [0.41; 16.81] | 1.07 [0.48; 2.40] | 3.12 [0.91; 10.73] | RADCAB | 1.14 [0.09; 14.67] |  |
| 5.78 [0.33; 101.08] | 5.10 [0.30; 87.00] | 2.13 [0.11; 40.22] | 2.98 [0.13; 70.35] | 1.21 [0.08; 17.75] | 3.54 [0.21; 60.70] | 1.14 [0.09; 14.67] | TECAB |  |

*Strip (-) indicates unavailability of direct treatment or between treatments produced same results with value 0, thus there is no CI.
