# Supplementary material for: Clinical outcomes in various coronary artery bypass grafting techniques: A systematic review and network meta-analysis
Source: PLoS One. 2026 Nov 5;21(11):e0360757. doi: 10.1371/journal.pone.0360757 (PMC13739143; doi:10.1371/journal.pone.0360757)
Supplement: Supplementary file 3 [file pone.0360757.s003.zip › Supplementary Table 20.docx]

# Supp. Table 20. The evidence findings for nine sub-techniques in reoperation

| **Certainty assessment** | | | | | | | **Effect** | **Certainty** |
| --- | --- | --- | --- | --- | --- | --- | --- | --- |
| **№ of studies** | **Study design** | **Risk of bias** | **Inconsistency** | **Indirectness** | **Imprecision** | **Publication bias** | **SMD with 95% CI** |  |

| **BH-ONCAB vs C-ONCAB** | | | | | | | | |  |
| --- | --- | --- | --- | --- | --- | --- | --- | --- | --- |
| **7** | Cohort Prospective, Cohort Retrospective | Serious | Not serious | Not serious | Not serious | Undetected | 0.90 [0.55; 1.47] | Moderate |  |
| **BH-ONCAB vs OPCAB** | | | | | | | | |  |
| **8** | Cohort Prospectiv, Cohort Retrospective | Serious | Not serious | Not serious | Not serious | Undetected | 1.76 [1.14; 2.72] | Moderate |  |
| **C-ONCAB vs MIDCAB** | | | | | | | | |  |
| **2** | Cohort Prospective, Cohort Retrospective | Serious | Serious | Not serious | Not serious | Undetected | 0.65 [0.14; 2.98] | Low |  |
| **C-ONCAB vs OPCAB** | | | | | | | | |  |
| **34** | Cohort Prospective, Cohort Retrospective | Serious | Not serious | Not serious | Not serious | Undetected | 1.16 [0.96; 1.40] | Moderate |  |
| **C-ONCAB vs PA-CABG** | | | | | | | | |  |
| **1** | Cohort Retrospective | Serious | Serious | Not serious | Not serious | Undetected | 0.09 [0.00;   1.73] | Low |  |
| **C-ONCAB vs RADCAB** | | | | | | | | |  |
| **2** | Cohort Prospective | Serious | Serious | Not serious | Not serious | Undetected | 2.32 [0.63;   8.54] | Low |  |
| **C-ONCAB vs TECAB** | | | | | | | | |  |
| **1** | Cohort Retrospective | Serious | Serious | Not serious | Not serious | Undetected | 0.22 [0.01;   5.01] | Low |  |
| **Endo-CABG vs MIDCAB** | | | | | | | | |  |
| **1** | Cohort Retrospective | Serious | Not serious | Not serious | Not serious | Undetected | 0.83 [0.08;   8.79] | Moderate |  |
| **Endo-CABG vs RADCAB** | | | | | | | | |  |
| **1** | Cohort Retrospective | Serious | Not serious | Not serious | Not serious | Undetected | 0.77 [0.08;   7.67] | Moderate |  |
| **MICS vs OPCAB** | | | | | | | | | |
| **2** | Cohort Prospective, Cohort Retrospective | Serious | Serious | Not serious | Not serious | Undetected | 0.77 [0.25;   2.34] | Low |  |
| **MIDCAB vs OPCAB** | | | | | | | | | |
| **8** | Cohort Prospective, Cohort Retrospective | Serious | Serious | Not serious | Not serious | Undetected | 2.01 [0.88;   4.58] | Low |  |
| **MIDCAB vs PA-CABG** | | | | | | | | |  |
| **1** | Cohort Prospective | Serious | Not serious | Not serious | Not serious | Undetected | 2.77 [0.10; 74.03] | Moderate |  |
| **MIDCAB vs RADCAB** | | | | | | | | | |
| **4** | Cohort Retrospective | Serious | Not serious | Not serious | Not serious | Undetected | 1.68 [1.08;   2.61] | Moderate |  |
| **MIDCAB vs TECAB** | | | | | | | | |  |
| **1** | Cohort Retrospective | Serious | Not Serious | Not serious | Not serious | Undetected | 0.35 [0.05;   2.57] | Moderate |  |
| **OPCAB vs RADCAB** | | | | | | | | | |
| **1** | Cohort Retrospective | Serious | Serious | Not serious | Not serious | Undetected | 6.43 [0.35; 117.36] | Low |  |
| **OPCAB vs TECAB** | | | | | | | | | |
| **1** | Cohort Retrospective | Serious | Serious | Not serious | Not serious | Undetected | 0.32 [0.01;   8.36] | Low |  |
| **PA-CABG vs TECAB** | | | | | | | | |  |
| **1** | Cohort Retrospective | Serious | Not serious | Not serious | Not serious | Undetected | 0.13 [0.01;   2.59] | Moderate |  |
| **RADCAB vs TECAB** | | | | | | | | |  |
| **1** | Cohort Retrospective | Serious | Not Serious | Not serious | Not serious | Undetected | 1.00 [0.06;  17.63] | Moderate |  |
