# Supplementary material for: Clinical outcomes in various coronary artery bypass grafting techniques: A systematic review and network meta-analysis
Source: PLoS One. 2026 Nov 5;21(11):e0360757. doi: 10.1371/journal.pone.0360757 (PMC13739143; doi:10.1371/journal.pone.0360757)
Supplement: Supplementary file 3 [file pone.0360757.s003.zip › Supplementary Table 21.docx]

# Supp. Table 21. The evidence findings for three main techniques in ventilation time

| **Certainty assessment** | | | | | | | **Effect** | **Certainty** |
| --- | --- | --- | --- | --- | --- | --- | --- | --- |
| **№ of studies** | **Study design** | **Risk of bias** | **Inconsistency** | **Indirectness** | **Imprecision** | **Publication bias** | **SMD with 95% CI** |  |

| **Conventional vs Minimal Invasive** | | | | | | | | | |  |
| --- | --- | --- | --- | --- | --- | --- | --- | --- | --- | --- |
| **15** | Cohort Prospective, Cohort Retrospective | Serious | Very serious | Not serious | Not serious | Undetected | 3.09 [ 2.04;  4.15] | Very Low |  |  |
| **Conventional vs Robotic** | | | | | | | | | |  |
| **8** | Cohort Prospective, Cohort Retrospective | Serious | Very serious | Not serious | Not serious | Undetected | 2.44 [ 0.85;  4.04] | Very Low |  |  |
| **Minimal Invasive vs Robotic** | | | | | | | | | |  |
| **4** | Cohort Prospective, Cohort Retrospective | Serious | Serious | Not serious | Not serious | Undetected | -0.82 [-2.34;  0.70] | Low |  |  |
| **Conventional vs Minimal Invasive (Indirect)** | | | | | | | | | |  |
| **15** | Cohort Prospective, Cohort Retrospective | Serious | Very serious | Not serious | Not serious | Undetected | 3.12 [ 2.17; 4.08] | Very low |  |  |
| **Conventional vs Robotic (Indirect)** | | | | | | | | | |  |
| **8** | Cohort Prospective, Cohort Retrospective | Serious | Very serious | Not serious | Not serious | Undetected | 2.45 [ 1.23; 3.67] | Very low |  |  |
| **Minimal Invasive vs Robotic (Indirect)** | | | | | | | | |  |  |
| **4** | Cohort Prospective, Cohort Retrospective | Serious | Serious | Not serious | Not serious | Undetected | -0.67 [-1.87; 0.53] | Low |  |  |
