# Supplementary material for: Clinical outcomes in various coronary artery bypass grafting techniques: A systematic review and network meta-analysis
Source: PLoS One. 2026 Nov 5;21(11):e0360757. doi: 10.1371/journal.pone.0360757 (PMC13739143; doi:10.1371/journal.pone.0360757)
Supplement: Supplementary file 3 [file pone.0360757.s003.zip › Supplementary Table 22.docx]

# Supp. Table 22. The evidence findings for nine sub-techniques in ventilation time

| **Certainty assessment** | | | | | | | **Effect** | **Certainty** |
| --- | --- | --- | --- | --- | --- | --- | --- | --- |
| **№ of studies** | **Study design** | **Risk of bias** | **Inconsistency** | **Indirectness** | **Imprecision** | **Publication bias** | **SMD with 95% CI** |  |

| **BH-ONCAB vs C-ONCAB** | | | | | | | | |  |
| --- | --- | --- | --- | --- | --- | --- | --- | --- | --- |
| **8** | Cohort Prospective, Cohort Retrospective | Serious | Not serious | Not serious | Not serious | Undetected | 1.42 [ -0.53; 3.36] | Moderate |  |
| **BH-ONCAB vs OPCAB** | | | | | | | | |  |
| **2** | Cohort Prospective, Cohort Retrospective | Serious | Not serious | Not serious | Not serious | Undetected | 3.51 [ 0.63; 6.39] | Moderate |  |
| **C-ONCAB vs MIDCAB** | | | | | | | | |  |
| **2** | Cohort Prospective, Cohort Retrospective | Serious | Very serious | Not serious | Not serious | Undetected | 7.93 [ 2.84; 13.03] | Very Low |  |
| **C-ONCAB vs OPCAB** | | | | | | | | |  |
| **15** | Cohort Prospective, Cohort Retrospective | Serious | Not serious | Not serious | Not serious | Undetected | 6.00 [ 4.35; 7.65] | Moderate |  |
| **C-ONCAB vs RADCAB** | | | | | | | | |  |
| **1** | Cohort Retrospective | Serious | Very serious | Not serious | Not serious | Undetected | 0.50 [ -3.88;  4.88] | Very Low |  |
| **C-ONCAB vs TECAB** | | | | | | | | |  |
| **1** | Cohort Prospective | Serious | Very serious | Not serious | Not serious | Undetected | -1.90 [-10.36;  6.56] | Very Low |  |
| **MICS vs OPCAB** | | | | | | | | | |
| **3** | Cohort Prospective, Cohort Retrospective | Serious | Very serious | Not serious | Not serious | Undetected | -2.36 [ -5.05;  0.33] | Very Low |  |
| **MIDCAB vs OPCAB** | | | | | | | | | |
| **9** | Cohort Prospective, Cohort Retrospective | Serious | Very serious | Not serious | Not serious | Undetected | -3.52 [-5.54; -1.50] | Very Low |  |
| **MIDCAB vs PA-CABG** | | | | | | | | |  |
| **1** | Cohort Retrospective | Serious | Not serious | Not serious | Not serious | Undetected | 2.60 [-2.05;  7.25] | Moderate |  |
| **MIDCAB vs RADCAB** | | | | | | | | | |
| **2** | Cohort Retrospective | Serious | Serious | Not serious | Not serious | Undetected | 1.38 [ -1.71;  4.46] | Low |  |
| **MIDCAB vs TECAB** | | | | | | | | |  |
| **2** | Cohort Prospective, Cohort Retrospective | Serious | Serious | Not serious | Not serious | Undetected | -3.49 [ -6.89; -0.09] | Low |  |
| **OPCAB vs RADCAB** | | | | | | | | | |
| **2** | Cohort Prospective, Cohort Retrospective | Serious | Very serious | Not serious | Not serious | Undetected | 1.71 [ -2.69;  6.10] | Very Low |  |
| **OPCAB vs TECAB** | | | | | | | | | |
| **1** | Cohort Retrospective | Serious | Very serious | Not serious | Not serious | Undetected | 5.00 [0.48;  9.52] | Very Low |  |
| **PA-CABG vs TECAB** | | | | | | | | |  |
| **1** | Cohort Retrospective | Serious | Serious | Not serious | Not serious | Undetected | -3.40 [ -7.92;  1.12] | Low |  |
| **RADCAB vs TECAB** | | | | | | | | |  |
| **4** | Cohort Retrospective | Serious | Not serious | Not serious | Not serious | Undetected | -1.15 [ -3.37;  1.07] | Moderate |  |
