# Supplementary material for: Clinical outcomes in various coronary artery bypass grafting techniques: A systematic review and network meta-analysis
Source: PLoS One. 2026 Nov 5;21(11):e0360757. doi: 10.1371/journal.pone.0360757 (PMC13739143; doi:10.1371/journal.pone.0360757)
Supplement: Supplementary file 3 [file pone.0360757.s003.zip › Supplementary Table 23.docx]

# Supp. Table 23. The evidence findings for three main techniques in wound infection

| **Certainty assessment** | | | | | | | **Effect** | **Certainty** |
| --- | --- | --- | --- | --- | --- | --- | --- | --- |
| **№ of studies** | **Study design** | **Risk of bias** | **Inconsistency** | **Indirectness** | **Imprecision** | **Publication bias** | **SMD with 95% CI** |  |

| **Conventional vs Minimal Invasive** | | | | | | | | | | |  |
| --- | --- | --- | --- | --- | --- | --- | --- | --- | --- | --- | --- |
| **13** | Cohort Prospective, Cohort Retrospective | Serious | Not serious | Not serious | Not serious | Undetected | 1.61 [1.01; 2.56] | Moderate |  |  |  |
| **Conventional vs Robotic** | | | | | | | | | | |  |
| **11** | Cohort Prospective, Cohort Retrospective | Serious | Not serious | Not serious | Not serious | Undetected | 2.55 [2.08; 3.13] | Moderate |  |  |  |
| **Minimal Invasive vs Robotic** | | | | | | | | | | |  |
| **3** | Cohort Retrospective | Serious | Not serious | Not serious | Not serious | Undetected | 2.90 [1.15; 7.27] | Moderate |  |  |  |
| **Conventional vs Minimal Invasive (Indirect)** | | | | | | | | | | |  |
| **13** | Cohort Prospective, Cohort Retrospective | Serious | Not serious | Not serious | Not serious | Undetected | 1.43 [0.94; 2.17] | Moderate |  |  |  |
| **Conventional vs Robotic (Indirect)** | | | | | | | | | | |  |
| **11** | Cohort Prospective, Cohort Retrospective | Serious | Not serious | Not serious | Not serious | Undetected | 2.61 [2.14; 3.19] | Moderate |  |  |  |
| **Minimal Invasive vs Robotic (Indirect)** | | | | | | | | | |  |  |
| **3** | Cohort Retrospective | Serious | Not serious | Not serious | Not serious | Undetected | 1.83 [1.17; 2.86] | Moderate |  |  |  |
