# Supplementary material for: Clinical outcomes in various coronary artery bypass grafting techniques: A systematic review and network meta-analysis
Source: PLoS One. 2026 Nov 5;21(11):e0360757. doi: 10.1371/journal.pone.0360757 (PMC13739143; doi:10.1371/journal.pone.0360757)
Supplement: Supplementary file 3 [file pone.0360757.s003.zip › Supplementary Table 24.docx]

# Supp. Table 24. The evidence findings for nine sub-techniques in wound infection

| **Certainty assessment** | | | | | | | **Effect** | **Certainty** |
| --- | --- | --- | --- | --- | --- | --- | --- | --- |
| **№ of studies** | **Study design** | **Risk of bias** | **Inconsistency** | **Indirectness** | **Imprecision** | **Publication bias** | **SMD with 95% CI** |  |

| **BH-ONCAB vs C-ONCAB** | | | | | | | | | | | | | |  |
| --- | --- | --- | --- | --- | --- | --- | --- | --- | --- | --- | --- | --- | --- | --- |
| **8** | Cohort Prospective, Cohort Retrospective | Serious | Not serious | Not serious | | | Not serious | Undetected | | | 0.89 [0.62; 1.28] | | Moderate |  |
| **BH-ONCAB vs OPCAB** | | | | | | | | | | | | | |  |
| **10** | Cohort Retrospective | Serious | Not serious | Not serious | | | Not serious | Undetected | | | 1.32 [0.98; 1.76] | | Moderate |  |
| **C-ONCAB vs MIDCAB** | | | | | | | | | | | | | |  |
| **2** | Cohort Prospective, Cohort Retrospective | Serious | Not serious | Not serious | | | Not serious | Undetected | | | 2.28 [0.40; 12.99] | | Moderate |  |
| **C-ONCAB vs OPCAB** | | | | | | | | | | | | | |  |
| **28** | Cohort Prospective, Cohort Retrospective | Serious | Not serious | Not serious | | | Not serious | Undetected | | | 1.25 [1.13; 1.39] | | Moderate |  |
| **C-ONCAB vs PA-CABG** | | | | | | | | | | | | | |  |
| **1** | Cohort Retrospective | Serious | Not serious | | Not serious | Not serious | | | Undetected | | | 2.81 [1.21; 6.55] | Moderate |  |
| **C-ONCAB vs RADCAB** | | | | | | | | | | | | | |  |
| **3** | Cohort Prospective, Cohort Retrospective | Serious | Not serious | | Not serious | Not serious | | | Undetected | | | 10.35 [1.95; 54.80] | Moderate |  |
| **C-ONCAB vs TECAB** | | | | | | | | | | | | | |  |
| **1** | Cohort Retrospective | Serious | Not serious | | Not serious | Not serious | | | Undetected | 8.84 [0.44; 177.66] | | | Moderate |  |
| **Endo-CABG vs MIDCAB** | | | | | | | | | | | | | |  |
| **1** | Cohort Retrospective | Serious | Not serious | Not serious | | | Not serious | Undetected | | | 0.29 [0.07;   1.30] | | Moderate |  |
| **Endo-CABG vs RADCAB** | | | | | | | | | | | | | |  |
| **1** | Cohort Retrospective | Serious | Not serious | Not serious | | | Not serious | Undetected | | | 1.57 [0.28;   8.73] | | Moderate |  |
| **MICS vs OPCAB** | | | | | | | | | | | | | | |
| **1** | Cohort Prospective | Serious | Not serious | Not serious | | | Not serious | Undetected | | | 0.21 [0.04;   1.00] | | Moderate |  |
| **MIDCAB vs OPCAB** | | | | | | | | | | | | | | |
| **7** | Cohort Prospective, Cohort Retrospective | Serious | Not serious | Not serious | | | Not serious | Undetected | | | 1.10 [0.57;   2.12] | | Moderate |  |
| **MIDCAB vs PA-CABG** | | | | | | | | | | | | | |  |
| **1** | Cohort Prospective | Serious | Not serious | Not serious | | | Not serious | Undetected | | | 0.33 [0.03;   3.30] | | Moderate |  |
| **MIDCAB vs RADCAB** | | | | | | | | | | | | | | |
| **3** | Cohort Retrospective | Serious | Not serious | Not serious | | | Not serious | Undetected | | | 3.39 [1.34;   8.60] | | Moderate |  |
| **OPCAB vs RADCAB** | | | | | | | | | | | | | | |
| **1** | Cohort Prospective | Serious | Not serious | Not serious | | | Not serious | Undetected | | | 9.77 [0.51; 186.52] | | Moderate |  |
| **OPCAB vs TECAB** | | | | | | | | | | | | | | |
| **1** | Cohort Retrospective | Serious | Not serious | Not serious | | | Not serious | Undetected | | | 0.32 [0.01;   7.87] | | Moderate |  |
| **RADCAB vs TECAB** | | | | | | | | | | | | | |  |
| **1** | Cohort Retrospective | Serious | Not serious | Not serious | | | Not serious | Undetected | | | 1.12 [0.05;  27.88] | | Moderate |  |
