# Supplementary material for: Clinical outcomes in various coronary artery bypass grafting techniques: A systematic review and network meta-analysis
Source: PLoS One. 2026 Nov 5;21(11):e0360757. doi: 10.1371/journal.pone.0360757 (PMC13739143; doi:10.1371/journal.pone.0360757)
Supplement: Supplementary file 3 [file pone.0360757.s003.zip › Supplementary Table 26.docx]

# Supp. Table 26. The evidence findings for nine sub-techniques in renal failure

| **Certainty assessment** | | | | | | | **Effect** | **Certainty** |
| --- | --- | --- | --- | --- | --- | --- | --- | --- |
| **№ of studies** | **Study design** | **Risk of bias** | **Inconsistency** | **Indirectness** | **Imprecision** | **Publication bias** | **SMD with 95% CI** |  |

| **BH-ONCAB vs C-ONCAB** | | | | | | | | | |
| --- | --- | --- | --- | --- | --- | --- | --- | --- | --- |
| **15** | Cohort Prospective, Cohort Retrospective | Serious | Not serious | Not serious | Not serious | Undetected | 0.95 [0.61; 1.48] | Moderate | |
| **BH-ONCAB vs OPCAB** | | | | | | | | | |
| **12** | Cohort Prospective, Cohort Retrospective | Serious | Not serious | Not serious | Not serious | Undetected | 2.37 [1.38; 4.07] | Moderate | |
| **C-ONCAB vs MIDCAB** | | | | | | | | | |
| **1** | Cohort Prospective | Serious | Very serious | Not serious | Not serious | Undetected | 3.63 [0.51; 25.92] | Very low | |
| **C-ONCAB vs OPCAB** | | | | | | | | | |
| **43** | Cohort Prospective, Cohort Retrospective | Serious | Not serious | Not serious | Not serious | Undetected | 1.39 [1.09; 1.76] | Moderate | |
| **C-ONCAB vs RADCAB** | | | | | | | | | |
| **3** | Cohort Prospective, Cohort Retrospective | Serious | Very serious | Not serious | Not serious | Undetected | 4.05 [0.63; 26.16] | Very low | |
| **Endo-CABG vs MIDCAB** | | | | | | | | | |
| **1** | Cohort Retrospective | Serious | Not serious | Not serious | Not serious | Undetected | 1.53 [0.32;  7.32] | Moderate | |
| **Endo-CABG vs RADCAB** | | | | | | | | | |
| **1** | Cohort Retrospective | Serious | Serious | Not serious | Not serious | Undetected | 2.16 [0.45; 10.44] | Low | |
| **MICS vs OPCAB** | | | | | | | | |  |
| **2** | Cohort Prospective | Serious | Very serious | Not serious | Not serious | Undetected | 0.84 [0.21;  3.37] | Very low | |
| **MIDCAB vs OPCAB** | | | | | | | | |  |
| **5** | Cohort Prospective | Serious | Very serious | Not serious | Not serious | Undetected | 0.27 [0.04;  1.83] | Very low | |
| **MIDCAB vs RADCAB** | | | | | | | | |  |
| **4** | Cohort Prospective, Cohort Retrospective | Serious | Serious | Not serious | Not serious | Undetected | 1.09 [0.46;  2.59] | Low | |
| **RADCAB vs TECAB** | | | | | | | | | |
| **1** | Cohort Retrospective | Serious | Not serious | Not serious | Not serious | Undetected | 1.14 [0.09; 14.67] | Moderate | |
