# Supplementary material for: Clinical outcomes in various coronary artery bypass grafting techniques: A systematic review and network meta-analysis
Source: PLoS One. 2026 Nov 5;21(11):e0360757. doi: 10.1371/journal.pone.0360757 (PMC13739143; doi:10.1371/journal.pone.0360757)
Supplement: Supplementary file 3 [file pone.0360757.s003.zip › Supplementary Table 28.docx]

# **Supp. Table 28. A-G**. Forest plots and netgraph in pair-wise meta-analysis of nine sub-techniques for secondary postoperative outcomes.

| **Outcome** | **Forest plot** | **Netgraph** |
| --- | --- | --- |
| **A. CVA** |    |  |
| **B. MI** |    |  |
| **C. Non-SR** |    |  |
| **D. Reoperation** |    |  |
| **E. Ventilation time** |    |  |
| **F. Wound infection** |    |  |
| **G. Renal failure** |    |  |
