# Supplementary material for: Clinical outcomes in various coronary artery bypass grafting techniques: A systematic review and network meta-analysis
Source: PLoS One. 2026 Nov 5;21(11):e0360757. doi: 10.1371/journal.pone.0360757 (PMC13739143; doi:10.1371/journal.pone.0360757)
Supplement: Supplementary file 3 [file pone.0360757.s003.zip › Supplementary Table 3.docx]

# Supp. Table 3. The evidence findings for three main techniques in operative times

| **Certainty assessment** | | | | | | | **Effect** | **Certainty** |
| --- | --- | --- | --- | --- | --- | --- | --- | --- |
| **№ of studies** | **Study design** | **Risk of bias** | **Inconsistency** | **Indirectness** | **Imprecision** | **Publication bias** | **SMD with 95% CI** |  |
| **Conventional vs Minimal Invasive** | | | | | | | | |
| **12** | Cohort Prospective, Cohort Retrospective | Serious | Very serious | Not serious | Not serious | Undetected | 5.31 [ -33.73;  44.35] | Very low |
| **Conventional vs Robotic** | | | | | | | | |
| **4** | Cohort Prospective, Cohort Retrospective | Serious | Very serious | Not serious | Not serious | Undetected | -19.38 [ -85.96;  47.21] | Very low |
| **Minimal Invasive vs Robotic** | | | | | | | | |
| **4** | Cohort Prospective, Cohort Retrospective | Serious | Serious | Not serious | Not serious | Undetected | -35.76 [-102.03;  30.52] | Low |
| **Conventional vs Minimal Invasive (Indirect)** | | | | | | | | |
| **12** | Cohort Prospective, Cohort Retrospective | Serious | Very serious | Not serious | Not serious | Undetected | 6.94 [-29.11; 42.99] | Very low |
| **Conventional vs Robotic (Indirect)** | | | | | | | |  |
| **4** | Cohort Prospective, Cohort Retrospective | Serious | Very serious | Not serious | Not serious | Undetected | -24.12 [-74.46; 26.22] | Very low |
| **Minimal Invasive vs Robotic (Indirect)** | | | | | | | | |
| **4** | Cohort Prospective, Cohort Retrospective | Serious | Serious | Not serious | Not serious | Undetected | -31.06 [-81.34; 19.22] | Low |
