# Supplementary material for: Clinical outcomes in various coronary artery bypass grafting techniques: A systematic review and network meta-analysis
Source: PLoS One. 2026 Nov 5;21(11):e0360757. doi: 10.1371/journal.pone.0360757 (PMC13739143; doi:10.1371/journal.pone.0360757)
Supplement: Supplementary file 3 [file pone.0360757.s003.zip › Supplementary Table 4.docx]

# Supp. Table 4. The evidence findings for nine sub-techniques in operative times

| **Certainty assessment** | | | | | | | **Effect** | **Certainty** |
| --- | --- | --- | --- | --- | --- | --- | --- | --- |
| **№ of studies** | **Study design** | **Risk of bias** | **Inconsistency** | **Indirectness** | **Imprecision** | **Publication bias** | **SMD with 95% CI** |  |
| **BH-ONCAB vs C-ONCAB** | | | | | | | | |
| **7** | Cohort Prospective, Cohort Retrospective, RCT | Serious | Not serious | Not serious | Not serious | Undetected | -2.66 [ -49.00;  43.69] | Moderate |
| **BH-ONCAB vs OPCAB** | | | | | | | | |
| **7** | Cohort Prospective, Cohort Retrospective,RCT | Serious | Not serious | Not serious | Not serious | Undetected | 31.35 [ -12.47;  75.18] | Moderate |
| **C-ONCAB vs MIDCAB** | | | | | | | | |
| **1** | Cohort Retrospective | Serious | Very serious | Not serious | Not serious | Undetected | 51.20 [ -66.84; 169.24] | Very low |
| **C-ONCAB vs OPCAB** | | | | | | | | |
| **16** | Cohort Prospective, Cohort Retrospective, RCT | Serious | Not serious | Not serious | Not serious | Undetected | 42.17 [  13.29;  71.06] | Moderate |
| **Endo-ONCAB vs MIDCAB** | | | | | | | | |
| **1** | Cohort Retrospective | Serious | Not serious | Not serious | Not serious | Undetected | 17.80 [ -96.56; 132.16] | Moderate |
| **Endo-ONCAB vs RADCAB** | | | | | | | | |
| **1** | Cohort Retrospective | Serious | Serious | Not serious | Not serious | Undetected | -111.00 [-225.36;   3.36] | Low |
| **MICS vs OPCAB** | | | | | | | | |
| **3** | Cohort Prospective, Cohort Retrospective | Serious | Not serious | Not serious | Not serious | Undetected | 51.60 [ -15.29; 118.49] | Moderate |
| **MIDCAB vs OPCAB** | | | | | | | | |
| **8** | Cohort Prospective, Cohort Retrospective | Serious | Very serious | Not serious | Not serious | Undetected | -29.44 [ -71.28;  12.39] | Very low |
| **MIDCAB vs PA-CABG** | | | | | | | | |
| **2** | Cohort Prospective, Cohort Retrospective | Serious | Not serious | Not serious | Not serious | Undetected | -57.47 [-139.56;  24.63] | Moderate |
| **MIDCAB vs RADCAB** | | | | | | | | |
| **3** | Cohort Retrospective | Serious | Not serious | Not serious | Not serious | Undetected | -43.26 [-109.26;  22.73] | Moderate |
| **MIDCAB vs TECAB** | | | | | | | | |
| **1** | Cohort Retrospective | Serious | Not serious | Not serious | Not serious | Undetected | -18.00 [-134.69;  98.69] | Moderate |
| **OPCAB vs PA-CABG** | | | | | | | | |
| **1** | Cohort Prospective | Serious | Serious | Not serious | Not serious | Undetected | -90.00 [-205.79;  25.79] | Low |
| **OPCAB vs RADCAB** | | | | | | | | |
| **3** | Cohort Prospective, Cohort Retrospective | Serious | Very serious | Not serious | Not serious | Undetected | -48.29 [-115.02;  18.44] | Very low |
| **PA-CABG vs TECAB** | | | | | | | | |
| **1** | Cohort Retrospective | Serious | Serious | Not serious | Not serious | Undetected | -12.00 [-127.23; 103.23] | Low |
| **RADCAB vs TECAB** | | | | | | | | |
| **4** | Cohort Retrospective | Serious | Not serious | Not serious | Not serious | Undetected | -11.88 [ -69.40;  45.64] | Moderate |
