# Supplementary material for: Clinical outcomes in various coronary artery bypass grafting techniques: A systematic review and network meta-analysis
Source: PLoS One. 2026 Nov 5;21(11):e0360757. doi: 10.1371/journal.pone.0360757 (PMC13739143; doi:10.1371/journal.pone.0360757)
Supplement: Supplementary file 3 [file pone.0360757.s003.zip › Supplementary Table 6.docx]

# Supp. Table 6. The evidence findings for nine sub-techniques in ICU stay

| **Certainty assessment** | | | | | | | **Effect** | **Certainty** | |
| --- | --- | --- | --- | --- | --- | --- | --- | --- | --- |
| **№ of studies** | **Study design** | **Risk of bias** | **Inconsistency** | **Indirectness** | **Imprecision** | **Publication bias** | **SMD with 95% CI** |  |  |
| **BH-ONCAB vs C-ONCAB** | | | | | | | | |  |
| **17** | Cohort Prospective, Cohort Retrospective | Serious | Not serious | Not serious | Not serious | Undetected | -0.15 [ -0.52;   0.22] | Moderate | |
| **BH-ONCAB vs OPCAB** | | | | | | | | |  |
| **121** | Cohort Prospective, Cohort Retrospective ,RCT | Serious | Not serious | Not serious | Not serious | Undetected | 0.32 [ -0.10;   0.74] | Moderate | |
| **C-ONCAB vs MICS** | | | | | | | | |  |
| **1** | Cohort Retrospective | Serious | Serious | Not serious | Not serious | Undetected | 0.70 [ -0.59;   1.99] | Low | |
| **C-ONCAB vs MIDCAB** | | | | | | | | |  |
| **2** | Cohort Retrospective | Serious | Very Serious | Not serious | Not serious | Undetected | 0.49 [ -0.45;   1.42] | Very Low | |
| **C-ONCAB vs OPCAB** | | | | | | | | |  |
| **29** | Cohort Prospective, Cohort Retrospective | Serious | Not serious | Not serious | Not serious | Undetected | 0.77 [  0.51;   1.03] | Moderate | |
| **C-ONCAB vs PA-CABG** | | | | | | | | |  |
| **1** | Cohort Retrospective | Serious | Serious | Not serious | Not serious | Undetected | 0.58 [ -0.58;   1.74] | Low | |
| **C-ONCAB vs RADCAB** | | | | | | | | |  |
| **1** | Cohort Retrospective | Serious | Very Serious | Not serious | Not serious | Undetected | 0.60 [ -0.61;   1.81]] | Very low | |
| **C-ONCAB vs TECAB** | | | | | | | | |  |
| **2** | Cohort Prospective, Cohort Retrospective | Serious | Serious | Not serious | Not serious | Undetected | 0.58 [ -0.28;   1.44] | Low | |
| **MICS vs MIDCAB** | | | | | | | | |  |
| **1** | Cohort Retrospective | Serious | Not serious | Not serious | Not serious | Undetected | -40.20 [-45.01; -35.39]] | Moderate | |
| **MICS vs OPCAB** | | | | | | | | |  |
| **4** | Cohort Prospective, Cohort Retrospective | Serious | Not serious | Not serious | Not serious | Undetected | -0.47 [ -1.07;   0.13] | Moderate | |
| **MIDCAB vs OPCAB** | | | | | | | | |  |
| **6** | Cohort Prospective, Cohort Retrospective | Serious | Very serious | Not serious | Not serious | Undetected | -0.07 [ -0.65;   0.52] | Very low | |
| **MIDCAB vs PA-CABG** | | | | | | | | |  |
| **3** | Cohort Prospective, Cohort Retrospective | Serious | Not serious | Not serious | Not serious | Undetected | -0.24 [ -0.99;   0.52] | Moderate | |
| **MIDCAB vs RADCAB** | | | | | | | | |  |
| **3** | Cohort Retrospective | Serious | Not Serious | Not serious | Not serious | Undetected | 0.18 [ -0.48;   0.85] | Moderate | |
| **MIDCAB vs TECAB** | | | | | | | | |  |
| **2** | Cohort Prospective, Cohort Retrospective | Serious | Not serious | Not serious | Not serious | Undetected | 0.10 [ -0.74;   0.95] | Moderate | |
| **OPCAB vs PA-CABG** | | | | | | | | |  |
| **1** | Cohort Prospective | Serious | Serious | Not serious | Not serious | Undetected | 0.00 [ -1.47;   1.47] | Low | |
| **OPCAB vs RADCAB** | | | | | | | | |  |
| **2** | Cohort Prospective, Cohort Retrospective | Serious | Very serious | Not serious | Not serious | Undetected | 4.13 [  2.89;   5.37] | Very low | |
| **PA-CABG vs RADCAB** | | | | | | | | |  |
| **1** | Cohort Retrospective | Serious | Serious | Not serious | Not serious | Undetected | 0.30 [ -0.87;   1.47] | Low | |
| **PA-CABG vs TECAB** | | | | | | | | |  |
| **1** | Cohort Retrospective | Serious | Serious | Not serious | Not serious | Undetected | 0.70 [ -0.70;   2.10] | Low | |
| **RADCAB vs TECAB** | | | | | | | | |  |
| **3** | Cohort Retrospective | Serious | Not serious | Not serious | Not serious | Undetected | -0.07 [ -0.86;   0.71] | Moderate | |
