# Supplementary material for: Clinical outcomes in various coronary artery bypass grafting techniques: A systematic review and network meta-analysis
Source: PLoS One. 2026 Nov 5;21(11):e0360757. doi: 10.1371/journal.pone.0360757 (PMC13739143; doi:10.1371/journal.pone.0360757)
Supplement: Supplementary file 3 [file pone.0360757.s003.zip › Supplementary Table 7.docx]

# Supp. Table 7. The evidence findings for three main techniques in length of stay

| **Certainty assessment** | | | | | | | **Effect** | **Certainty** |
| --- | --- | --- | --- | --- | --- | --- | --- | --- |
| **№ of studies** | **Study design** | **Risk of bias** | **Inconsistency** | **Indirectness** | **Imprecision** | **Publication bias** | **SMD with 95% CI** |  |
| **Conventional vs Minimal Invasive** | | | | | | | | |
| **15** | Cohort Prospective, Cohort Retrospective | Serious | Serious | Not serious | Not serious | Undetected | 2.73 [ 1.95;  3.50] | Low |
| **Conventional vs Robotic** | | | | | | | | |
| **15** | Cohort Prospective, Cohort Retrospective | Serious | Serious | Not serious | Not serious | Undetected | 2.62 [ 1.85;  3.40] | Low |
| **Minimal Invasive vs Robotic** | | | | | | | | |
| **6** | Cohort Prospective, Cohort Retrospective | Serious | Not serious | Not serious | Not serious | Undetected | 1.77 [ 0.62;  2.93] | Moderate |
| **Conventional vs Minimal Invasive (Indirect)** | | | | | | | | |
| **15** | Cohort Prospective, Cohort Retrospective | Serious | Serious | Not serious | Not serious | Undetected | 2.34 [ 1.65;  3.02] | Low |
| **Conventional vs Robotic (Indirect)** | | | | | | | | |
| **15** | Cohort Prospective, Cohort Retrospective | Serious | Serious | Not serious | Not serious | Undetected | 2.97 [ 2.29;  3.66] | Low |
| **Minimal Invasive vs Robotic (Indirect)** | | | | | | | | |
| **6** | Cohort Prospective, Cohort Retrospective | Serious | Not serious | Not serious | Not serious | Undetected | 0.63 [-0.17;  1.44] | Moderate |
