# Supplementary material for: Clinical outcomes in various coronary artery bypass grafting techniques: A systematic review and network meta-analysis
Source: PLoS One. 2026 Nov 5;21(11):e0360757. doi: 10.1371/journal.pone.0360757 (PMC13739143; doi:10.1371/journal.pone.0360757)
Supplement: Supplementary file 3 [file pone.0360757.s003.zip › Supplementary Table 8.docx]

# Supp. Table 8. The evidence findings for nine sub-techniques in length of stay

| **Certainty assessment** | | | | | | | **Effect** | **Certainty** | |
| --- | --- | --- | --- | --- | --- | --- | --- | --- | --- |
| **№ of studies** | **Study design** | **Risk of bias** | **Inconsistency** | **Indirectness** | **Imprecision** | **Publication bias** | **SMD with 95% CI** |  |  |
| **BH-ONCAB vs C-ONCAB** | | | | | | | | |  |
| **11** | Cohort Prospective, Cohort Retrospective ,RCT | Serious | Not serious | Not serious | Not serious | Undetected | -0.53 [ -1.28;  0.22] | Moderate | |
| **BH-ONCAB vs OPCAB** | | | | | | | | |  |
| **10** | Cohort Prospective, Cohort Retrospective ,RCT | Serious | Not serious | Not serious | Not serious | Undetected | 0.93 [  0.25;  1.61] | Moderate | |
| **C-ONCAB vs MIDCAB** | | | | | | | | |  |
| **3** | Cohort Retrospective | Serious | Very serious | Not serious | Not serious | Undetected | 1.98 [  0.65;  3.31] | Very low | |
| **C-ONCAB vs OPCAB** | | | | | | | | |  |
| **32** | Cohort Prospective, Cohort Retrospective,RCT | Serious | Not serious | Not serious | Not serious | Undetected | 1.14 [  0.76;  1.52] | Moderate | |
| **C-ONCAB vs PA-CABG** | | | | | | | | |  |
| **1** | Cohort Retrospective | Serious | Not serious | Not serious | Not serious | Undetected | 8.60 [  6.77; 10.43] | Moderate | |
| **C-ONCAB vs RADCAB** | | | | | | | | |  |
| **2** | Cohort Prospective, Cohort Retrospective | Serious | Very serious | Not serious | Not serious | Undetected | 1.64 [  0.32;  2.95] | Very low | |
| **C-ONCAB vs TECAB** | | | | | | | | |  |
| **2** | Cohort Prospective, Cohort Retrospective | Serious | Serious | Not serious | Not serious | Undetected | 5.01 [  3.46;  6.57] | Low | |
| **Endo-CABG vs MIDCAB** | | | | | | | | |  |
| **1** | Cohort Retrospective | Serious | Not serious | Not serious | Not serious | Undetected | 0.00 [ -1.78;  1.78] | Moderate | |
| **Endo-CABG vs RADCAB** | | | | | | | | |  |
| **1** | Cohort Retrospective | Serious | Serious | Not serious | Not serious | Undetected | -0.47 [ -1.07;   0.13] | Low | |
| **MICS vs OPCAB** | | | | | | | | |  |
| **3** | Cohort Retrospective | Serious | Not serious | Not serious | Not serious | Undetected | -1.54 [ -2.79; -0.30] | Moderate | |
| **MIDCAB vs OPCAB** | | | | | | | | |  |
| **8** | Cohort Prospective, Cohort Retrospective | Serious | Very serious | Not serious | Not serious | Undetected | -2.04 [ -2.76; -1.32] | Very low | |
| **MIDCAB vs PA-CABG** | | | | | | | | |  |
| **2** | Cohort Prospective, Cohort Retrospective | Serious | Not serious | Not serious | Not serious | Undetected | -0.70 [ -2.26;  0.86] | Moderate | |
| **MIDCAB vs RADCAB** | | | | | | | | |  |
| **4** | Cohort Retrospective | Serious | Not serious | Not serious | Not serious | Undetected | 1.05 [  0.16;  1.93] | Moderate | |
| **MIDCAB vs TECAB** | | | | | | | | |  |
| **2** | Cohort Prospective, Cohort Retrospective | Serious | Not serious | Not serious | Not serious | Undetected | 2.99 [  1.46;  4.51] | Moderate | |
| **OPCAB vs PA-CABG** | | | | | | | | |  |
| **1** | Cohort Prospective, | Serious | Serious | Not serious | Not serious | Undetected | 0.00 [ -2.52;  2.52] | Low | |
| **OPCAB vs RADCAB** | | | | | | | | |  |
| **4** | Cohort Prospective, Cohort Retrospective | Serious | Very serious | Not serious | Not serious | Undetected | 1.57 [  0.53;  2.61] | Very low | |
| **PA-CABG vs TECAB** | | | | | | | | |  |
| **1** | Cohort Retrospective | Serious | Serious | Not serious | Not serious | Undetected | 1.50 [ -0.51;  3.51] | Low | |
| **RADCAB vs TECAB** | | | | | | | | |  |
| **1** | Cohort Retrospective | Serious | Not serious | Not serious | Not serious | Undetected | -0.50 [ -2.28;  1.28] | Moderate | |
