# Supplementary material for: Clinical outcomes in various coronary artery bypass grafting techniques: A systematic review and network meta-analysis
Source: PLoS One. 2026 Nov 5;21(11):e0360757. doi: 10.1371/journal.pone.0360757 (PMC13739143; doi:10.1371/journal.pone.0360757)
Supplement: Supplementary file 3 [file pone.0360757.s003.zip › Supplementary Table 9.docx]

# Supp. Table 9. The evidence findings for three main techniques in mortality

| **Certainty assessment** | | | | | | | **Effect** | **Certainty** |
| --- | --- | --- | --- | --- | --- | --- | --- | --- |
| **№ of studies** | **Study design** | **Risk of bias** | **Inconsistency** | **Indirectness** | **Imprecision** | **Publication bias** | **SMD with 95% CI** |  |
| **Conventional vs Minimal Invasive** | | | | | | | | |
| **19** | Cohort Prospective, Cohort Retrospective | Serious | Not serious | Not serious | Not serious | Undetected | 1.58 [0.81; 3.06] | Moderate |
| **Conventional vs Robotic** | | | | | | | | |
| **12** | Cohort Prospective, Cohort Retrospective | Serious | Not serious | Not serious | Not serious | Undetected | 2.87 [2.14; 3.85] | Moderate |
| **Minimal Invasive vs Robotic** | | | | | | | | |
| **5** | Cohort Prospective, Cohort Retrospective | Serious | Not serious | Not serious | Not serious | Undetected | 0.71 [0.29; 1.70] | Moderate |
| **Conventional vs Minimal Invasive (Indirect)** | | | | | | | | |
| **19** | Cohort Prospective, Cohort Retrospective | Serious | Not serious | Not serious | Not serious | Undetected | 2.18 [1.27; 3.73] | Moderate |
| **Conventional vs Robotic (Indirect)** | | | | | | | | |
| **12** | Cohort Prospective, Cohort Retrospective | Serious | Not serious | Not serious | Not serious | Undetected | 2.70 [2.03; 3.58] | Moderate |
| **Minimal Invasive vs Robotic (Indirect)** | | | | | | | | |
| **5** | Cohort Prospective, Cohort Retrospective | Serious | Not serious | Not serious | Not serious | Undetected | 1.24 [0.71; 2.17] | Moderate |
