# Supplementary material for: Clinical outcomes in various coronary artery bypass grafting techniques: A systematic review and network meta-analysis
Source: PLoS One. 2026 Nov 5;21(11):e0360757. doi: 10.1371/journal.pone.0360757 (PMC13739143; doi:10.1371/journal.pone.0360757)
Supplement: Supplementary file 5 [file pone.0360757.s006.docx]

Dear Associate Editor and Reviewer,

We sincerely appreciate the valuable feedback that we have received from the associate editor and the reviewerr regarding our manuscript, PONE-D-25-53785R1. We have marked the changes we made in the manuscript with the track changes feature. We would also like to give our response to each piece of feedback and suggestion we have received.

**Associate Editor**

- **Comment 1:** Please ensure that your manuscript meets PLOS ONE's style requirements, including those for file naming. The PLOS ONE style templates can be found at https://journals.plos.org/plosone/s/file?id=wjVg/PLOSOne_formatting_sample_main_body.pdf and https://journals.plos.org/plosone/s/file?id=ba62/PLOSOne_formatting_sample_title_authors_affiliations.pdf
  - **Answer 1:** We have followed the format that was provided in the template.
- **Comment 2 :** We note that you have indicated that there are restrictions to data sharing for this study. For studies involving human research participant data or other sensitive data, we encourage authors to share de-identified or anonymized data. However, when data cannot be publicly shared for ethical reasons, we allow authors to make their data sets available upon request. For information on unacceptable data access restrictions, please see <http://journals.plos.org/plosone/s/data-availability#loc-unacceptable-data-access-restrictions>.
  - **Answer 2:** We have written a data availability statement in our manuscript. All of our data are available in the manuscript and supplementary files.
- **Comment 3 : Before we proceed with your manuscript, please address the following prompts:**
  - If there are ethical or legal restrictions on sharing a de-identified data set, please explain them in detail (e.g., data contain potentially identifying or sensitive patient information, data are owned by a third-party organization, etc.) and who has imposed them (e.g., a Research Ethics Committee or Institutional Review Board, etc.). Please also provide contact information for a data access committee, ethics committee, or other institutional body to which data requests may be sent.
  - If there are no restrictions, please upload the minimal anonymized data set necessary to replicate your study findings to a stable, public repository and provide us with the relevant URLs, DOIs, or accession numbers. Please see http://www.bmj.com/content/340/bmj.c181.long for guidelines on how to de-identify and prepare clinical data for publication. For a list of recommended repositories, please see https://journals.plos.org/plosone/s/recommended-repositories. You also have the option of uploading the data as Supporting Information files, but we would recommend depositing data directly to a data repository if possible.
    - **Answer 3:** This is not applicable, but our search strategy has been registered in PROSPERO with the ID CRD42023480325. We have also written a data availability statement in our manuscript. All of our data are available in the manuscript and supplementary files.
- **Comment 4 :** Please include a separate caption for each figure in your manuscript.
  - **Answer 4:** It has been added in the figure legend section. We do apologize for our mistakes, Figure 3-5 were supposed to become Table 1,4, and 5.
- **Comment 5 :** Please include a copy of Table 3 which you refer to in your text on pages 8 and 9.
  - **Answer 5:** We have misnumbered the table 3 previously. It has been revised.
- **Comment 6 :** Please include captions for your Supporting Information files at the end of your manuscript, and update any in-text citations to match accordingly. Please see our Supporting Information guidelines for more information: <http://journals.plos.org/plosone/s/supporting-information>
  - **Answer 6:** We have included the captions for each supporting file in the “Supporting Files Caption” in the manuscript. We have also provided the captions in each supporting file separately.
- **Comment 7 :** Is the manuscript technically sound, and do the data support the conclusions?
  - **Answer 7:** We appreciate the editor's assessment. We would like to clarify that our data have supported our conclusion, but there are some limitations to consider in interpreting the results. We have refined the conclusion to better reflect the balance between the supportive data and the inherent limitations of current literature, emphasizing the exploratory nature of the findings.

**Reviewer 1**

- **Comment 1 :** Inherent Limitations of Included Evidence: The most significant limitation, correctly identified by the authors, is the scarcity of RCTs (only 8 out of 132 studies). The vast majority of the data comes from observational studies, which are highly susceptible to confounding by indication. The observed advantages of R-CABG could be partly explained by its selective use in younger, healthier patients with less complex disease, as suggested by the baseline characteristics.
  - **Answer 1:** We sincerely thank the reviewer for the review and this insightful comment, which highlights a critical nuance in our findings. We fully agree that the predominance of observational studies in our network meta-analysis introduces a risk of confounding by indication, and have mentioned this in our discussion and limitations section.
- **Comment 2 :**  Handling of Heterogeneity: While the authors acknowledge high heterogeneity for some outcomes (e.g., operative time, ICU stay) and attribute it to the variety of techniques, this remains a concern. The statement that heterogeneity was "equal in all interventions" for length of stay does not fully resolve the issue, as it complicates the interpretation of the pooled estimates.
  - **Answer 2:** We have revised this sentence to provide more clarity. We intended to say that the heterogeneity was likely to be caused by the wide variety of techniques analyzed.
- **Comment 3 :**
  - The abstract states mortality and complete revascularization are primary outcomes, but the results for complete revascularization are presented very briefly. The finding that all techniques had "comparable revascularization rates" is a crucial result that deserves more prominence in the abstract and discussion.
  - The manuscript states "the rest will be secondary outcomes." This should be rephrased to the past tense, e.g., "were classified as secondar**y** outcomes."
    - **Answer 3:** We have added more details regarding complete revascularization in the abstract and discussion sections. The use of the past tense has been applied in the manuscript.
- **Comment 4 :**
  - **Presentation and Typos:**
    - The tables and figures referenced in the text (e.g., Table 1, Figure 5) are included as images from the statistical software and are often blurry, poorly formatted, and difficult to read. They need to be recreated as clear, professional figures.
      - **Answer 4.1:** Thank you for highlighting this concern. We understand that clarity is essential for interprating our data. We have revised the presentation of our results by resizing them to ensure better readability. We have verified that these new versions remain clear and legible across different viewing platforms. The readability will also enhanced greatly if the reviewer download the files (tables or figures) directly from the manuscript link available from the publisher.
    - There are numerous typographical errors in the text and tables that need careful proofreading. For example:
      - Page 13, Line 231: "BH-ONCAB(119 patients)), but there is 2 studies" -> "are 2 studies"
      - Page 17, Line 437: "observationalstudies" -> "observational studies"
      - Page 37, Table 2: "Indo-CABG" is likely a typo for "Endo-CABG".
      - The forest plots in the results tables (e.g., Pages 39, 42, 51) contain placeholder text and are not interpretable in their current state
        - **Answer 4.2:** All of them have been revised.
- **Comment 5:**
  - Abstract:
    - Briefly mention the key limitation (predominance of observational data) in the conclusion to immediately contextualize the findings for the reader.
      - **Answer 5.1:** We have revised it accordance to your suggestion.
    - Expand slightly on the "comparable revascularization rates" finding, as this is a primary outcome.
      - **Answer 5.2:** We have added more details regarding this outcome in the abstract.
- **Comment 6:**
  - Introduction & Discussion:
    - Strengthen the Discussion on Confounding: While acknowledged, the discussion of how baseline differences (LVEF, number of vessels) might bias the results in favor of less invasive techniques should be more substantive. Discuss the potential impact of unmeasured confounders (e.g., surgeon volume, institutional expertise) more explicitly.
      - **Answer 6.1:** We have written more about this in our discussion section.
    - Discuss Complete Revascularization: Elaborate on the clinical importance of finding comparable complete revascularization rates across techniques. This is a critical point that reassures clinicians that less invasive approaches may not compromise this key surgical goal.
      - **Answer 6.2:** We have elaborated more on the clinical importance of complete revascularization in our discussion section.
    - Refine the "Techniques Challenges" Section: The paragraph on the learning curve (lines 416-425) is somewhat repetitive. Streamline this and consider citing a specific study that quantifies the learning curve for R-CABG.
      - **Answer 6.3:** We have revised this section in accordance to your suggestion.
- **Comment 7:**
  - Methods:
  - Outcome Definitions: Consider providing brief definitions for key outcomes (e.g., what constituted "complete revascularization," how "renal failure" was defined across studies) to help readers understand potential clinical variation.
    - **Answer 7:** We have added the definition of our outcomes.
- **Comment 8:**
  - Results:
  - Improve Data Presentation: This is the highest priority. All tables and figures must be recreated for clarity. Forest plots should be legible, with clear labels for treatments and effect estimates. The current images are not publication-ready.
    - **Answer 8.1:** We sincerely appreciate the reviewer’s feedback regarding the visual presentation. We have resized all figures and forest plots to a much larger scale to ensure they are fully legible. We have also verified that all labels and effect estimates are clearly readable in the final version of the manuscript. The readability will also enhanced greatly if the reviewer download the files (tables or figures) directly from the manuscript link available from the publisher.
  - Clarify Sub-technique Results: In the results for the nine sub-techniques, it is often stated that outcomes were "comparable." For the reader's benefit, consider adding a summary statement or table highlighting which specific sub-technique comparisons were statistically significant, as these are the most actionable findings.
    - **Answer 8.2:** Thank you for highlighting this aspect. If the outcomes were statistically significant, we have written them in the results section, and the ranking table also gave the ranking number and mark of significance as provided in tables 2 and 3.
- **Comment 9:**
  - **Copyediting:**
  - A thorough proofread is essential to correct grammatical errors, typos, and inconsistent formatting (e.g., "C-CABG" vs. "C-CABG," "node-splitting" vs. "node splitting").
    - **Answer 9.1:** We have rechecked our manuscript and revised all the terms, as well as made the terms uniform.
  - Priority of Revisions
    - Critical: Recreate all tables and figures to a professional, readable standard.
    - High: Perform a thorough copyedit to fix all grammatical and typographical errors.
    - High: Expand the discussion on confounding and the limitations of observational data.
    - Medium: Enhance the discussion of the primary outcome of complete revascularization.
      - **Answer 9.2:** We have revised all of these, in accordance to your suggestions.
