# Supplementary material for: Clinical outcomes in various coronary artery bypass grafting techniques: A systematic review and network meta-analysis
Source: PLoS One. 2026 Nov 5;21(11):e0360757. doi: 10.1371/journal.pone.0360757 (PMC13739143; doi:10.1371/journal.pone.0360757)
Supplement: Supplementary file 6 [file pone.0360757.s007.docx]

**Response to Reviewers**

**Manuscript:** Clinical outcomes in various coronary artery bypass grafting techniques: a systematic review and network meta-analysis

Dear Editor and Reviewers,

We thank you for the opportunity to revise our manuscript, and we thank the reviewers for their careful reading and constructive comments. Below we respond to each point in turn. Reviewer comments are shown in bold italics, followed by our response. All changes are tracked in the accompanying "Revised Manuscript with Track Changes" file.

## **Reviewer #2**

**Comment 1:** Despite claiming a full proofread, the manuscript body contains clear typographical errors.

- **Response:** We apologize for these oversights and have now conducted a further, more thorough proofread of the entire manuscript body. We identified and corrected more than two dozen typographical and grammatical errors throughout the Introduction, Methods, Results, Discussion/Limitations, and supporting-file legends, including (among others):
  - "learning cruve" → "learning curve"; "aimsed" → "aimed" (Introduction)
  - "unvailable" → "unavailable"; "exceededing" → "exceeding"; subject–verb agreement fixes such as "The primary outcome were" → "The primary outcomes were" and "These principle were applied" → "These principles were applied" (Methods)
  - "insignifcant" → "insignificant"; "similiar" → "similar"; "exhibitied" → "exhibited"; a duplicated sentence in the GRADE evidence paragraph removed; a stray parenthesis/period corrected (Results)
  - "LFEF" → "LVEF"; "cofounders" → "confounders"; "instituional" → "institutional" (Limitations)
  - "competing intention for completing this review" → "no competing interests related to this review"; "Data avalability" → "Data availability"; "Quality assesment" → "Quality assessment"; "non sinus rhytm" → "non-sinus rhythm" (Declarations / supporting-file legends)
- All corrections are marked as tracked changes in the revised manuscript.

**Comment 2:** Reference [17], cited for the Begg-Mazumdar publication bias test, appears to be an unrelated clubfoot paper; Reference [23], cited for the GRADE approach, appears to be a workplace sedentary-time trial. Both need to be corrected to the canonical citations.

- **Response:** We thank the reviewer for catching this reference-management error. Both citations have been corrected to the canonical sources:
- Reference [17] is now: Begg CB, Mazumdar M. Operating characteristics of a rank correlation test for publication bias. Biometrics 1994;50:1088–101.
- Reference [23] is now: Guyatt GH, Oxman AD, Akl EA, Kunz R, Vist G, Brozek J, et al. GRADE guidelines: 1. Introduction—GRADE evidence profiles and summary of findings tables. J Clin Epidemiol 2011;64:383–94.
- We also re-checked every remaining reference against its corresponding in-text citation to confirm there were no further mismatches.

## **Reviewer #3**

**Comment 1:** Substantial differences in baseline characteristics, particularly LVEF and the number of diseased vessels, may affect transitivity and introduce confounding in the network comparisons. Although this limitation is acknowledged, its potential impact on the treatment estimates and rankings requires further consideration.

- **Response:** We agree this deserved fuller discussion. We have expanded the Limitations section (Section 4.2) to explain explicitly how imbalance in LVEF and diseased-vessel count could violate the transitivity assumption underlying the network comparisons, and we now state that where a technique was preferentially used in patients with more favorable baseline characteristics, its apparent advantage in the treatment estimates and P-score ranking may partly reflect this case-mix imbalance rather than a true treatment effect. We have added a sentence explicitly cautioning that the rankings should be interpreted as hypothesis-generating rather than definitive evidence of superiority for this reason.

**Comment 2:** Several outcomes show very high heterogeneity (I² >90%). The potential sources and implications of this heterogeneity should be discussed more clearly, particularly when interpreting the pooled estimates and treatment rankings.

- **Response:** We have added text to the Limitations section identifying the likely sources of the very high heterogeneity observed for several outcomes (I² >90%), principally differences across studies in patient case-mix, surgical technique and operator experience, follow-up duration, and outcome ascertainment, and we now explicitly note that such high heterogeneity widens the uncertainty around the pooled estimates and reduces confidence in the corresponding treatment rankings, so these particular outcomes should be interpreted with additional caution and viewed as exploratory.

**Comment 3:** Given the predominance of observational studies, the authors should be more cautious in interpreting P-scores and rankings as evidence of treatment superiority. The potential impact of combining ITT and as-treated analyses should also be clarified.

- **Response:** We have added two clarifications to the Limitations section addressing both parts of this comment. First, on combining analysis principles, we now explain that as-treated analyses can favor the received treatment by excluding crossovers or non-adherent patients, whereas intention-to-treat analyses preserve randomization but may dilute effect estimates, so pooling both approaches could bias effect estimates in either direction and adds a further source of between-study heterogeneity not fully captured by I² alone. Second, we have added an explicit caution, given that the network was dominated by observational studies subject to confounding by indication, that P-scores and the resulting treatment rankings should not be read as evidence of one technique's superiority over another; rather, they should be regarded as a summary of the relative ordering of effect estimates within this evidence base, to be confirmed by future randomized trials.

We believe these revisions fully address the reviewers' comments and have improved the clarity, accuracy, and rigor of the manuscript. We thank the editor and reviewers again for their time and constructive feedback.
