# Supplementary material for: Clinical outcomes in various coronary artery bypass grafting techniques: A systematic review and network meta-analysis
Source: PLoS One. 2026 Nov 5;21(11):e0360757. doi: 10.1371/journal.pone.0360757 (PMC13739143; doi:10.1371/journal.pone.0360757)
Supplement: Peer review history

# Peer review: Decision Letter 0

**DOI:** 10.1371/journal.pone.0360757.r001

**Authors:** Irfan Ullah

26 Dec 2025

Dear Dr. hanafy,

Thank you for submitting your manuscript to PLOS ONE. After careful consideration, we feel that it has merit but does not fully meet PLOS ONE’s publication criteria as it currently stands. Therefore, we invite you to submit a revised version of the manuscript that addresses the points raised during the review process.

Please submit your revised manuscript by Feb 09 2026 11:59PM. If you will need more time than this to complete your revisions, please reply to this message or contact the journal office at plosone@plos.org. When you're ready to submit your revision, log on to https://www.editorialmanager.com/pone/ and select the 'Submissions Needing Revision' folder to locate your manuscript file.

- A letter that responds to each point raised by the academic editor and reviewer(s). You should upload this letter as a separate file labeled 'Response to Reviewers'.
- A marked-up copy of your manuscript that highlights changes made to the original version. You should upload this as a separate file labeled 'Revised Manuscript with Track Changes'.
- An unmarked version of your revised paper without tracked changes. You should upload this as a separate file labeled 'Manuscript'.

If you would like to make changes to your financial disclosure, please include your updated statement in your cover letter. Guidelines for resubmitting your figure files are available below the reviewer comments at the end of this letter.

If applicable, we recommend that you deposit your laboratory protocols in protocols.io to enhance the reproducibility of your results. Protocols.io assigns your protocol its own identifier (DOI) so that it can be cited independently in the future. For instructions see: https://journals.plos.org/plosone/s/submission-guidelines#loc-laboratory-protocols. Additionally, PLOS ONE offers an option for publishing peer-reviewed Lab Protocol articles, which describe protocols hosted on protocols.io. Read more information on sharing protocols at https://plos.org/protocols?utm_medium=editorial-email&utm_source=authorletters&utm_campaign=protocols.

We look forward to receiving your revised manuscript.

Kind regards,

Irfan Ullah, MBBS, MPH

Academic Editor

PLOS One

Journal Requirements:

When submitting your revision, we need you to address these additional requirements.

1. Please ensure that your manuscript meets PLOS ONE’s style requirements, including those for file naming. The PLOS ONE style templates can be found at

https://journals.plos.org/plosone/s/file?id=wjVg/PLOSOne_formatting_sample_main_body.pdf and https://journals.plos.org/plosone/s/file?id=ba62/PLOSOne_formatting_sample_title_authors_affiliations.pdf

2. We note that you have indicated that there are restrictions to data sharing for this study. For studies involving human research participant data or other sensitive data, we encourage authors to share de-identified or anonymized data. However, when data cannot be publicly shared for ethical reasons, we allow authors to make their data sets available upon request. For information on unacceptable data access restrictions, please see http://journals.plos.org/plosone/s/data-availability#loc-unacceptable-data-access-restrictions.

Before we proceed with your manuscript, please address the following prompts:

a) If there are ethical or legal restrictions on sharing a de-identified data set, please explain them in detail (e.g., data contain potentially identifying or sensitive patient information, data are owned by a third-party organization, etc.) and who has imposed them (e.g., a Research Ethics Committee or Institutional Review Board, etc.). Please also provide contact information for a data access committee, ethics committee, or other institutional body to which data requests may be sent.

b) If there are no restrictions, please upload the minimal anonymized data set necessary to replicate your study findings to a stable, public repository and provide us with the relevant URLs, DOIs, or accession numbers. Please see http://www.bmj.com/content/340/bmj.c181.long for guidelines on how to de-identify and prepare clinical data for publication. For a list of recommended repositories, please see https://journals.plos.org/plosone/s/recommended-repositories. You also have the option of uploading the data as Supporting Information files, but we would recommend depositing data directly to a data repository if possible.

Please update your Data Availability statement in the submission form accordingly.

3. Please include a separate caption for each figure in your manuscript.

4. Please include a copy of Table 3 which you refer to in your text on pages 8 and 9.

5. Please include captions for your Supporting Information files at the end of your manuscript, and update any in-text citations to match accordingly. Please see our Supporting Information guidelines for more information: http://journals.plos.org/plosone/s/supporting-information.

If the reviewer comments include a recommendation to cite specific previously published works, please review and evaluate these publications to determine whether they are relevant and should be cited. There is no requirement to cite these works unless the editor has indicated otherwise.

[Note: HTML markup is below. Please do not edit.]

Reviewers' comments:

Reviewer’s Responses to Questions

**Comments to the Author**

1. Is the manuscript technically sound, and do the data support the conclusions?

Reviewer #1: Yes

**********

2. Has the statistical analysis been performed appropriately and rigorously?

Reviewer #1: Yes

**********

3. Have the authors made all data underlying the findings in their manuscript fully available?

The PLOS Data policy

Reviewer #1: Yes

**********

4. Is the manuscript presented in an intelligible fashion and written in standard English?

Reviewer #1: No

**********

Reviewer #1: The authors reported their work named “Clinical outcomes in various coronary artery bypass grafting techniques: a systematic review and network meta-analysis”. This is a timely and extensive systematic review and network meta-analysis (NMA) that synthesizes a large body of evidence (132 studies, ~780,000 patients) comparing conventional, minimally invasive, and robotic CABG techniques. The topic is of significant clinical interest as surgical approaches evolve. The study is generally well-designed, follows PRISMA-NMA guidelines, and employs rigorous statistical methodology. I have the following comments:

Major Strengths

1. Comprehensive Scope: The analysis goes beyond the three main techniques (C-CABG, MI-CABG, R-CABG) to include nine sub-techniques, providing a much more granular and clinically relevant comparison.

2. Rigorous Methodology: The use of frequentist NMA, assessment of transitivity, evaluation of consistency via node-splitting, and comprehensive assessment of heterogeneity and publication bias are major strengths. The pre-registration of the protocol adds to its credibility.

3. Transparent Reporting: The authors are transparent about the limitations, particularly the reliance on observational studies, the significant differences in baseline patient characteristics (e.g., ejection fraction, number of diseased vessels), and the potential influence of a single large study (Cavallaro et al., 2015).

4. GRADE Assessment: Applying the GRADE framework to evaluate the certainty of evidence is a critical and commendable step that helps contextualize the findings for clinicians and policymakers.

Major Weaknesses and Points for Clarification

1. Inherent Limitations of Included Evidence: The most significant limitation, correctly identified by the authors, is the scarcity of RCTs (only 8 out of 132 studies). The vast majority of the data comes from observational studies, which are highly susceptible to confounding by indication. The observed advantages of R-CABG could be partly explained by its selective use in younger, healthier patients with less complex disease, as suggested by the baseline characteristics.

2. Handling of Heterogeneity: While the authors acknowledge high heterogeneity for some outcomes (e.g., operative time, ICU stay) and attribute it to the variety of techniques, this remains a concern. The statement that heterogeneity was "equal in all interventions" for length of stay does not fully resolve the issue, as it complicates the interpretation of the pooled estimates.

3. Clarity on Primary vs. Secondary Outcomes:

o The abstract states mortality and complete revascularization are primary outcomes, but the results for complete revascularization are presented very briefly. The finding that all techniques had "comparable revascularization rates" is a crucial result that deserves more prominence in the abstract and discussion.

o The manuscript states "the rest will be secondary outcomes." This should be rephrased to the past tense, e.g., "were classified as secondary outcomes."

4. Presentation and Typos:

o The tables and figures referenced in the text (e.g., Table 1, Figure 5) are included as images from the statistical software and are often blurry, poorly formatted, and difficult to read. They need to be recreated as clear, professional figures.

o There are numerous typographical errors in the text and tables that need careful proofreading. For example:

� Page 13, Line 231: "BH-ONCAB(119 patients)), but there is 2 studies" -> "are 2 studies"

� Page 17, Line 437: "observationalstudies" -> "observational studies"

� Page 37, Table 2: "Indo-CABG" is likely a typo for "Endo-CABG".

� The forest plots in the results tables (e.g., Pages 39, 42, 51) contain placeholder text and are not interpretable in their current state.

Specific Recommendations for Revision

1. Abstract:

o Briefly mention the key limitation (predominance of observational data) in the conclusion to immediately contextualize the findings for the reader.

o Expand slightly on the "comparable revascularization rates" finding, as this is a primary outcome.

2. Introduction & Discussion:

o Strengthen the Discussion on Confounding: While acknowledged, the discussion of how baseline differences (LVEF, number of vessels) might bias the results in favor of less invasive techniques should be more substantive. Discuss the potential impact of unmeasured confounders (e.g., surgeon volume, institutional expertise) more explicitly.

o Discuss Complete Revascularization: Elaborate on the clinical importance of finding comparable complete revascularization rates across techniques. This is a critical point that reassures clinicians that less invasive approaches may not compromise this key surgical goal.

o Refine the "Techniques Challenges" Section: The paragraph on the learning curve (lines 416-425) is somewhat repetitive. Streamline this and consider citing a specific study that quantifies the learning curve for R-CABG.

3. Methods:

o Outcome Definitions: Consider providing brief definitions for key outcomes (e.g., what constituted "complete revascularization," how "renal failure" was defined across studies) to help readers understand potential clinical variation.

4. Results:

o Improve Data Presentation: This is the highest priority. All tables and figures must be recreated for clarity. Forest plots should be legible, with clear labels for treatments and effect estimates. The current images are not publication-ready.

o Clarify Sub-technique Results: In the results for the nine sub-techniques, it is often stated that outcomes were "comparable." For the reader’s benefit, consider adding a summary statement or table highlighting which specific sub-technique comparisons were statistically significant, as these are the most actionable findings.

5. Copyediting:

o A thorough proofread is essential to correct grammatical errors, typos, and inconsistent formatting (e.g., "C-CABG" vs. "C-CABG," "node-splitting" vs. "node splitting").

Priority of Revisions

• Critical: Recreate all tables and figures to a professional, readable standard.

• High: Perform a thorough copyedit to fix all grammatical and typographical errors.

• High: Expand the discussion on confounding and the limitations of observational data.

• Medium: Enhance the discussion of the primary outcome of complete revascularization.

**********

what does this mean?). If published, this will include your full peer review and any attached files.

If you choose “no”, your identity will remain anonymous but your review may still be made public.

**Do you want your identity to be public for this peer review?**  For information about this choice, including consent withdrawal, please see our Privacy Policy

Reviewer #1: **Yes:**  Mohamed Rahouma, MD PhD MS MSc FAHA

**********

[NOTE: If reviewer comments were submitted as an attachment file, they will be attached to this email and accessible via the submission site. Please log into your account, locate the manuscript record, and check for the action link "View Attachments". If this link does not appear, there are no attachment files.]

To ensure your figures meet our technical requirements, please review our figure guidelines: https://journals.plos.org/plosone/s/figures

You may also use PLOS’s free figure tool, NAAS, to help you prepare publication quality figures: https://journals.plos.org/plosone/s/figures#loc-tools-for-figure-preparation.

NAAS will assess whether your figures meet our technical requirements by comparing each figure against our figure specifications.

# Author comment: Author response to Decision Letter 1

**DOI:** 10.1371/journal.pone.0360757.r002

23 Mar 2026

Dear Associate Editor and Reviewer,

We sincerely appreciate the valuable feedback that we have received from the associate editor and the reviewerr regarding our manuscript, PONE-D-25-53785R1. We have marked the changes we made in the manuscript with the track changes feature. We would also like to give our response to each piece of feedback and suggestion we have received.

Associate Editor

Comment 1: Please ensure that your manuscript meets PLOS ONE’s style requirements, including those for file naming. The PLOS ONE style templates can be found at https://journals.plos.org/plosone/s/file?id=wjVg/PLOSOne_formatting_sample_main_body.pdf and https://journals.plos.org/plosone/s/file?id=ba62/PLOSOne_formatting_sample_title_authors_affiliations.pdf

Answer 1: We have followed the format that was provided in the template.

Comment 2 : We note that you have indicated that there are restrictions to data sharing for this study. For studies involving human research participant data or other sensitive data, we encourage authors to share de-identified or anonymized data. However, when data cannot be publicly shared for ethical reasons, we allow authors to make their data sets available upon request. For information on unacceptable data access restrictions, please see http://journals.plos.org/plosone/s/data-availability#loc-unacceptable-data-access-restrictions.

Answer 2: We have written a data availability statement in our manuscript. All of our data are available in the manuscript and supplementary files.

Comment 3 : Before we proceed with your manuscript, please address the following prompts:

If there are ethical or legal restrictions on sharing a de-identified data set, please explain them in detail (e.g., data contain potentially identifying or sensitive patient information, data are owned by a third-party organization, etc.) and who has imposed them (e.g., a Research Ethics Committee or Institutional Review Board, etc.). Please also provide contact information for a data access committee, ethics committee, or other institutional body to which data requests may be sent.

If there are no restrictions, please upload the minimal anonymized data set necessary to replicate your study findings to a stable, public repository and provide us with the relevant URLs, DOIs, or accession numbers. Please see http://www.bmj.com/content/340/bmj.c181.long for guidelines on how to de-identify and prepare clinical data for publication. For a list of recommended repositories, please see https://journals.plos.org/plosone/s/recommended-repositories. You also have the option of uploading the data as Supporting Information files, but we would recommend depositing data directly to a data repository if possible.

Answer 3: This is not applicable, but our search strategy has been registered in PROSPERO with the ID CRD42023480325. We have also written a data availability statement in our manuscript. All of our data are available in the manuscript and supplementary files.

Comment 4 : Please include a separate caption for each figure in your manuscript.

Answer 4: It has been added in the figure legend section. We do apologize for our mistakes, Figure 3-5 were supposed to become Table 1,4, and 5.

Comment 5 : Please include a copy of Table 3 which you refer to in your text on pages 8 and 9.

Answer 5: We have misnumbered the table 3 previously. It has been revised.

Comment 6 : Please include captions for your Supporting Information files at the end of your manuscript, and update any in-text citations to match accordingly. Please see our Supporting Information guidelines for more information: http://journals.plos.org/plosone/s/supporting-information

Answer 6: We have included the captions for each supporting file in the “Supporting Files Caption” in the manuscript. We have also provided the captions in each supporting file separately.

Comment 7 : Is the manuscript technically sound, and do the data support the conclusions?

Answer 7: We appreciate the editor’s assessment. We would like to clarify that our data have supported our conclusion, but there are some limitations to consider in interpreting the results. We have refined the conclusion to better reflect the balance between the supportive data and the inherent limitations of current literature, emphasizing the exploratory nature of the findings.

Reviewer 1

Comment 1 : Inherent Limitations of Included Evidence: The most significant limitation, correctly identified by the authors, is the scarcity of RCTs (only 8 out of 132 studies). The vast majority of the data comes from observational studies, which are highly susceptible to confounding by indication. The observed advantages of R-CABG could be partly explained by its selective use in younger, healthier patients with less complex disease, as suggested by the baseline characteristics.

Answer 1: We sincerely thank the reviewer for the review and this insightful comment, which highlights a critical nuance in our findings. We fully agree that the predominance of observational studies in our network meta-analysis introduces a risk of confounding by indication, and have mentioned this in our discussion and limitations section.

Comment 2 : Handling of Heterogeneity: While the authors acknowledge high heterogeneity for some outcomes (e.g., operative time, ICU stay) and attribute it to the variety of techniques, this remains a concern. The statement that heterogeneity was "equal in all interventions" for length of stay does not fully resolve the issue, as it complicates the interpretation of the pooled estimates.

Answer 2: We have revised this sentence to provide more clarity. We intended to say that the heterogeneity was likely to be caused by the wide variety of techniques analyzed.

Comment 3 :

The abstract states mortality and complete revascularization are primary outcomes, but the results for complete revascularization are presented very briefly. The finding that all techniques had "comparable revascularization rates" is a crucial result that deserves more prominence in the abstract and discussion.

The manuscript states "the rest will be secondary outcomes." This should be rephrased to the past tense, e.g., "were classified as secondary outcomes."

Answer 3: We have added more details regarding complete revascularization in the abstract and discussion sections. The use of the past tense has been applied in the manuscript.

Comment 4 :

Presentation and Typos:

The tables and figures referenced in the text (e.g., Table 1, Figure 5) are included as images from the statistical software and are often blurry, poorly formatted, and difficult to read. They need to be recreated as clear, professional figures.

Answer 4.1: Thank you for highlighting this concern. We understand that clarity is essential for interprating our data. We have revised the presentation of our results by resizing them to ensure better readability. We have verified that these new versions remain clear and legible across different viewing platforms. The readability will also enhanced greatly if the reviewer download the files (tables or figures) directly from the manuscript link available from the publisher.

There are numerous typographical errors in the text and tables that need careful proofreading. For example:

Page 13, Line 231: "BH-ONCAB(119 patients)), but there is 2 studies" -> "are 2 studies"

Page 17, Line 437: "observationalstudies" -> "observational studies"

Page 37, Table 2: "Indo-CABG" is likely a typo for "Endo-CABG".

The forest plots in the results tables (e.g., Pages 39, 42, 51) contain placeholder text and are not interpretable in their current state

Answer 4.2: All of them have been revised.

Comment 5:

Abstract:

Briefly mention the key limitation (predominance of observational data) in the conclusion to immediately contextualize the findings for the reader.

Answer 5.1: We have revised it accordance to your suggestion.

Expand slightly on the "comparable revascularization rates" finding, as this is a primary outcome.

Answer 5.2: We have added more details regarding this outcome in the abstract.

Comment 6:

Introduction & Discussion:

Strengthen the Discussion on Confounding: While acknowledged, the discussion of how baseline differences (LVEF, number of vessels) might bias the results in favor of less invasive techniques should be more substantive. Discuss the potential impact of unmeasured confounders (e.g., surgeon volume, institutional expertise) more explicitly.

Answer 6.1: We have written more about this in our discussion section.

Discuss Complete Revascularization: Elaborate on the clinical importance of finding comparable complete revascularization rates across techniques. This is a critical point that reassures clinicians that less invasive approaches may not compromise this key surgical goal.

Answer 6.2: We have elaborated more on the clinical importance of complete revascularization in our discussion section.

Refine the "Techniques Challenges" Section: The paragraph on the learning curve (lines 416-425) is somewhat repetitive. Streamline this and consider citing a specific study that quantifies the learning curve for R-CABG.

Answer 6.3: We have revised this section in accordance to your suggestion.

Comment 7:

Methods:

Outcome Definitions: Consider providing brief definitions for key outcomes (e.g., what constituted "complete revascularization," how "renal failure" was defined across studies) to help readers understand potential clinical variation.

Answer 7: We have added the definition of our outcomes.

Comment 8:

Results:

Improve Data Presentation: This is the highest priority. All tables and figures must be recreated for clarity. Forest plots should be legible, with clear labels for treatments and effect estimates. The current images are not publication-ready.

Answer 8.1: We sincerely appreciate the reviewer’s feedback regarding the visual presentation. We have resized all figures and forest plots to a much larger scale to ensure they are fully legible. We have also verified that all labels and effect estimates are clearly readable in the final version of the manuscript. The readability will also enhanced greatly if the reviewer download the files (tables or figures) directly from the manuscript link available from the publisher.

Clarify Sub-technique Results: In the results for the nine sub-techniques, it is often stated that outcomes were "comparable." For the reader’s benefit, consider adding a summary statement or table highlighting which specific sub-technique comparisons were statistically significant, as these are the most actionable findings.

Answer 8.2: Thank you for highlighting this aspect. If the outcomes were statistically significant, we have written them in the results section, and the ranking table also gave the ranking number and mark of significance as provided in tables 2 and 3.

Comment 9:

Copyediting:

A thorough proofread is essential to correct grammatical errors, typos, and inconsistent formatting (e.g., "C-CABG" vs. "C-CABG," "node-splitting" vs. "node splitting").

Answer 9.1: We have rechecked our manuscript and revised all the terms, as well as made the terms uniform.

Priority of Revisions

Critical: Recreate all tables and figures to a professional, readable standard.

High: Perform a thorough copyedit to fix all grammatical and typographical errors.

High: Expand the discussion on confounding and the limitations of observational data.

Medium: Enhance the discussion of the primary outcome of complete revascularization.

Answer 9.2: We have revised all of these, in accordance to your suggestions.

Attachment Submitted filename: Responses to Reviewers.docx

# Peer review: Decision Letter 1

**DOI:** 10.1371/journal.pone.0360757.r003

**Authors:** Chiara Lazzeri

25 Sep 2026

Dear Dr. hanafy,

Thank you for submitting your manuscript to PLOS One. After careful consideration, we feel that it has merit but does not fully meet PLOS One’s publication criteria as it currently stands. Therefore, we invite you to submit a revised version of the manuscript that addresses the points raised during the review process.

Please submit your revised manuscript by Nov 09 2026 11:59PM. If you will need more time than this to complete your revisions, please reply to this message or contact the journal office at plosone@plos.org. When you're ready to submit your revision, log on to https://www.editorialmanager.com/pone/ and select the 'Submissions Needing Revision' folder to locate your manuscript file.

- A letter that responds to each point raised by the academic editor and reviewer(s). You should upload this letter as a separate file labeled 'Response to Reviewers'.
- A marked-up copy of your manuscript that highlights changes made to the original version. You should upload this as a separate file labeled 'Revised Manuscript with Track Changes'.
- An unmarked version of your revised paper without tracked changes. You should upload this as a separate file labeled 'Manuscript'.

If you would like to make changes to your financial disclosure, please include your updated statement in your cover letter. Guidelines for resubmitting your figure files are available below the reviewer comments at the end of this letter.

If applicable, we recommend that you deposit your laboratory protocols in protocols.io to enhance the reproducibility of your results. Protocols.io assigns your protocol its own identifier (DOI) so that it can be cited independently in the future. For instructions see: https://journals.plos.org/plosone/s/submission-guidelines#loc-laboratory-protocols. Additionally, PLOS One offers an option for publishing peer-reviewed Lab Protocol articles, which describe protocols hosted on protocols.io. Read more information on sharing protocols at https://plos.org/protocols?utm_medium=editorial-email&utm_source=authorletters&utm_campaign=protocols.

As the corresponding author, your ORCID iD is verified in the submission system and will appear in the published article. PLOS supports the use of ORCID, and we encourage all coauthors to register for an ORCID iD and use it as well. Please encourage your coauthors to verify their ORCID iD within the submission system before final acceptance, as unverified ORCID iDs will not appear in the published article. *Only*  the individual author can complete the verification step; PLOS staff *cannot*  verify ORCID iDs on behalf of authors.

We look forward to receiving your revised manuscript.

Kind regards,

Chiara Lazzeri

Academic Editor

PLOS One

Journal Requirements:

If the reviewer comments include a recommendation to cite specific previously published works, please review and evaluate these publications to determine whether they are relevant and should be cited. There is no requirement to cite these works unless the editor has indicated otherwise.

Please review your reference list to ensure that it is complete and correct. If you have cited papers that have been retracted, please include the rationale for doing so in the manuscript text, or remove these references and replace them with relevant current references. Any changes to the reference list should be mentioned in the rebuttal letter that accompanies your revised manuscript. If you need to cite a retracted article, indicate the article’s retracted status in the References list and also include a citation and full reference for the retraction notice.

[Note: HTML markup is below. Please do not edit.]

Reviewers' comments:

Reviewer’s Responses to Questions

**Comments to the Author**

Reviewer #2: All comments have been addressed

Reviewer #3: (No Response)

**********

2. Is the manuscript technically sound, and do the data support the conclusions?

Reviewer #2: Partly

Reviewer #3: No

**********

3. Has the statistical analysis been performed appropriately and rigorously?

Reviewer #2: Yes

Reviewer #3: No

**********

4. Have the authors made all data underlying the findings in their manuscript fully available?

The PLOS Data policy

Reviewer #2: Yes

Reviewer #3: No

**********

5. Is the manuscript presented in an intelligible fashion and written in standard English?

Reviewer #2: Yes

Reviewer #3: Yes

**********

Reviewer #2: Despite claiming a full proofread, the manuscript body contains clear typographical errors.

Reference [17], cited for the Begg-Mazumdar publication bias test, appears to be an unrelated clubfoot paper; Reference [23], cited for the GRADE approach, appears to be a workplace sedentary-time trial. Both need to be corrected to the canonical citations.

Reviewer #3: The revised manuscript has been improved and addresses several of the concerns raised previously. However, some important methodological issues remain.

First, substantial differences in baseline characteristics, particularly LVEF and the number of diseased vessels, may affect transitivity and introduce confounding in the network comparisons. Although this limitation is acknowledged, its potential impact on the treatment estimates and rankings requires further consideration.

Second, several outcomes show very high heterogeneity (I² >90%). The potential sources and implications of this heterogeneity should be discussed more clearly, particularly when interpreting the pooled estimates and treatment rankings.

Finally, given the predominance of observational studies, the authors should be more cautious in interpreting P-scores and rankings as evidence of treatment superiority. The potential impact of combining ITT and as-treated analyses should also be clarified.

Overall, the manuscript is potentially valuable, but these issues should be addressed before publication.

**********

what does this mean?). If published, this will include your full peer review and any attached files.

If you choose “no”, your identity will remain anonymous but your review may still be made public.

**Do you want your identity to be public for this peer review?**  For information about this choice, including consent withdrawal, please see our Privacy Policy

Reviewer #2: **Yes:**  Amr Arafat

Reviewer #3: No

**********

[NOTE: If reviewer comments were submitted as an attachment file, they will be attached to this email and accessible via the submission site. Please log into your account, locate the manuscript record, and check for the action link "View Attachments". If this link does not appear, there are no attachment files.]

To ensure your figures meet our technical requirements, please review our figure guidelines: https://journals.plos.org/plosone/s/figures

You may also use PLOS’s free figure tool, NAAS, to help you prepare publication quality figures: https://journals.plos.org/plosone/s/figures#loc-tools-for-figure-preparation.

NAAS will assess whether your figures meet our technical requirements by comparing each figure against our figure specifications.

# Author comment: Author response to Decision Letter 2

**DOI:** 10.1371/journal.pone.0360757.r004

27 Sep 2026

Dear Editor and Reviewers,

We thank you for the opportunity to revise our manuscript, and we thank the reviewers for their careful reading and constructive comments. Below we respond to each point in turn. Reviewer comments are shown in bold italics, followed by our response. All changes are tracked in the accompanying "Revised Manuscript with Track Changes" file.

Reviewer #2

Comment 1: Despite claiming a full proofread, the manuscript body contains clear typographical errors.

Response: We apologize for these oversights and have now conducted a further, more thorough proofread of the entire manuscript body. We identified and corrected more than two dozen typographical and grammatical errors throughout the Introduction, Methods, Results, Discussion/Limitations, and supporting-file legends, including (among others):

"learning cruve" → "learning curve"; "aimsed" → "aimed" (Introduction)

"unvailable" → "unavailable"; "exceededing" → "exceeding"; subject–verb agreement fixes such as "The primary outcome were" → "The primary outcomes were" and "These principle were applied" → "These principles were applied" (Methods)

"insignifcant" → "insignificant"; "similiar" → "similar"; "exhibitied" → "exhibited"; a duplicated sentence in the GRADE evidence paragraph removed; a stray parenthesis/period corrected (Results)

"LFEF" → "LVEF"; "cofounders" → "confounders"; "instituional" → "institutional" (Limitations)

"competing intention for completing this review" → "no competing interests related to this review"; "Data avalability" → "Data availability"; "Quality assesment" → "Quality assessment"; "non sinus rhytm" → "non-sinus rhythm" (Declarations / supporting-file legends)

All corrections are marked as tracked changes in the revised manuscript.

Comment 2: Reference [17], cited for the Begg-Mazumdar publication bias test, appears to be an unrelated clubfoot paper; Reference [23], cited for the GRADE approach, appears to be a workplace sedentary-time trial. Both need to be corrected to the canonical citations.

Response: We thank the reviewer for catching this reference-management error. Both citations have been corrected to the canonical sources:

Reference [17] is now: Begg CB, Mazumdar M. Operating characteristics of a rank correlation test for publication bias. Biometrics 1994;50:1088–101.

Reference [23] is now: Guyatt GH, Oxman AD, Akl EA, Kunz R, Vist G, Brozek J, et al. GRADE guidelines: 1. Introduction—GRADE evidence profiles and summary of findings tables. J Clin Epidemiol 2011;64:383–94.

We also re-checked every remaining reference against its corresponding in-text citation to confirm there were no further mismatches.

Reviewer #3

Comment 1: Substantial differences in baseline characteristics, particularly LVEF and the number of diseased vessels, may affect transitivity and introduce confounding in the network comparisons. Although this limitation is acknowledged, its potential impact on the treatment estimates and rankings requires further consideration.

Response: We agree this deserved fuller discussion. We have expanded the Limitations section (Section 4.2) to explain explicitly how imbalance in LVEF and diseased-vessel count could violate the transitivity assumption underlying the network comparisons, and we now state that where a technique was preferentially used in patients with more favorable baseline characteristics, its apparent advantage in the treatment estimates and P-score ranking may partly reflect this case-mix imbalance rather than a true treatment effect. We have added a sentence explicitly cautioning that the rankings should be interpreted as hypothesis-generating rather than definitive evidence of superiority for this reason.

Comment 2: Several outcomes show very high heterogeneity (I² >90%). The potential sources and implications of this heterogeneity should be discussed more clearly, particularly when interpreting the pooled estimates and treatment rankings.

Response: We have added text to the Limitations section identifying the likely sources of the very high heterogeneity observed for several outcomes (I² >90%), principally differences across studies in patient case-mix, surgical technique and operator experience, follow-up duration, and outcome ascertainment, and we now explicitly note that such high heterogeneity widens the uncertainty around the pooled estimates and reduces confidence in the corresponding treatment rankings, so these particular outcomes should be interpreted with additional caution and viewed as exploratory.

Comment 3: Given the predominance of observational studies, the authors should be more cautious in interpreting P-scores and rankings as evidence of treatment superiority. The potential impact of combining ITT and as-treated analyses should also be clarified.

Response: We have added two clarifications to the Limitations section addressing both parts of this comment. First, on combining analysis principles, we now explain that as-treated analyses can favor the received treatment by excluding crossovers or non-adherent patients, whereas intention-to-treat analyses preserve randomization but may dilute effect estimates, so pooling both approaches could bias effect estimates in either direction and adds a further source of between-study heterogeneity not fully captured by I² alone. Second, we have added an explicit caution, given that the network was dominated by observational studies subject to confounding by indication, that P-scores and the resulting treatment rankings should not be read as evidence of one technique’s superiority over another; rather, they should be regarded as a summary of the relative ordering of effect estimates within this evidence base, to be confirmed by future randomized trials.

We believe these revisions fully address the reviewers' comments and have improved the clarity, accuracy, and rigor of the manuscript. We thank the editor and reviewers again for their time and constructive feedback.

Attachment Submitted filename: Response to Reviewers.docx

# Editor report: Decision Letter 2

**DOI:** 10.1371/journal.pone.0360757.r005

**Authors:** Chiara Lazzeri

30 Sep 2026

Clinical outcomes in various coronary artery bypass grafting techniques: a systematic review and network meta-analysis

PONE-D-25-53785R2

Dear Dr. hanafy,

We’re pleased to inform you that your manuscript has been judged scientifically suitable for publication and will be formally accepted for publication once it meets all outstanding technical requirements.

Within one week, you’ll receive an e-mail detailing the required amendments. When these have been addressed, you’ll receive a formal acceptance letter and your manuscript will be scheduled for publication.

An invoice will be generated when your article is formally accepted. Please note, if your institution has a publishing partnership with PLOS and your article meets the relevant criteria, all or part of your publication costs will be covered. Please make sure your user information is up-to-date by logging into Editorial Manager at Editorial Manager® and clicking the ‘Update My Information' link at the top of the page. For questions related to billing, please contact billing support.

If your institution or institutions have a press office, please notify them about your upcoming paper to help maximize its impact. If they’ll be preparing press materials, please inform our press team as soon as possible -- no later than 48 hours after receiving the formal acceptance. Your manuscript will remain under strict press embargo until 2 pm Eastern Time on the date of publication. For more information, please contact onepress@plos.org.

Kind regards,

Chiara Lazzeri

Academic Editor

PLOS One

Additional Editor Comments (optional):

Reviewers' comments:

# Editor report: Acceptance letter

**DOI:** 10.1371/journal.pone.0360757.r006

**Authors:** Chiara Lazzeri

PONE-D-25-53785R2

PLOS One

Dear Dr. hanafy,

I'm pleased to inform you that your manuscript has been deemed suitable for publication in PLOS One. Congratulations! Your manuscript is now being handed over to our production team.

At this stage, our production department will prepare your paper for publication. This includes ensuring the following:

* All references, tables, and figures are properly cited

* All relevant supporting information is included in the manuscript submission,

* There are no issues that prevent the paper from being properly typeset

You will receive further instructions from the production team, including instructions on how to review your proof when it is ready. Please keep in mind that we are working through a large volume of accepted articles, so please give us a few days to review your paper and let you know the next and final steps.

Lastly, if your institution or institutions have a press office, please let them know about your upcoming paper now to help maximize its impact. If they'll be preparing press materials, please inform our press team within the next 48 hours. Your manuscript will remain under strict press embargo until 2 pm Eastern Time on the date of publication. For more information, please contact onepress@plos.org.

You will receive an invoice from PLOS for your publication fee after your manuscript has reached the completed accept phase. If you receive an email requesting payment before acceptance or for any other service, this may be a phishing scheme. Learn how to identify phishing emails and protect your accounts at https://explore.plos.org/phishing.

If we can help with anything else, please email us at customercare@plos.org.

Thank you for submitting your work to PLOS One and supporting open access.

Kind regards,

PLOS One Editorial Office Staff

on behalf of

Dr. Chiara Lazzeri

Academic Editor

PLOS One
